# Native Semisynthesis of Isopeptide-Linked Substrates for Specificity Analysis of Deubiquitinases and Ubl Proteases

**DOI:** 10.1021/jacs.3c04062

**Published:** 2023-09-15

**Authors:** Zhou Zhao, Rachel O’Dea, Kim Wendrich, Nafizul Kazi, Malte Gersch

**Affiliations:** † Chemical Genomics Centre, Max Planck Institute of Molecular Physiology, Otto-Hahn-Str. 15, 44227 Dortmund, Germany; ‡ Department of Chemistry and Chemical Biology, TU Dortmund University, Otto-Hahn-Str. 15, 44227 Dortmund, Germany

## Abstract

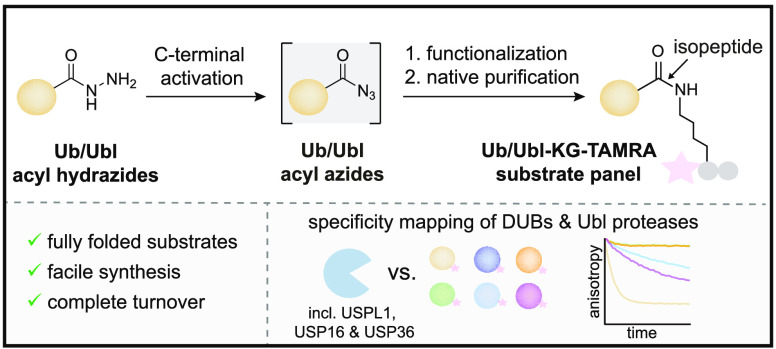

Post-translational modifications with ubiquitin (Ub) and ubiquitin-like proteins (Ubls) are regulated by isopeptidases termed deubiquitinases (DUBs) and Ubl proteases. Here, we describe a mild chemical method for the preparation of fluorescence polarization substrates for these enzymes that is based on the activation of C-terminal Ub/Ubl hydrazides to acyl azides and their subsequent functionalization to isopeptides. The procedure is complemented by native purification routes and thus circumvents the previous need for desulfurization and refolding. Its broad applicability was demonstrated by the generation of fully cleavable substrates for Ub, SUMO1, SUMO2, NEDD8, ISG15, and Fubi. We employed these reagents for the investigation of substrate specificities of human UCHL3, USPL1, USP2, USP7, USP16, USP18, and USP36. Pronounced selectivity of USPL1 for SUMO2/3 over SUMO1 was observed, which we rationalize with crystal structures and biochemical assays, revealing a SUMO paralogue specificity mechanism distinct from SENP family deSUMOylases. Moreover, we investigated the recently identified Fubi proteases USP16 and USP36 and found both to act as bona fide deFubiylases, harboring catalytic activity against isopeptide-linked Fubi. Surprisingly, we also noticed the activity of both enzymes toward ISG15, previously not identified in chemoproteomics, which makes USP16 and USP36 the first human DUBs with specific isopeptidase activity toward three distinct modifiers. The methods described here for the preparation of isopeptide-linked, fully folded substrates will aid in the characterization of further DUBs/Ubl proteases. More broadly, our findings highlight possible limitations associated with fluorogenic substrates and Ubl activity-based probes and stress the importance of isopeptide-containing reagents for validating isopeptidase activities and quantifying substrate specificities.

## Introduction

**Figure 1:**
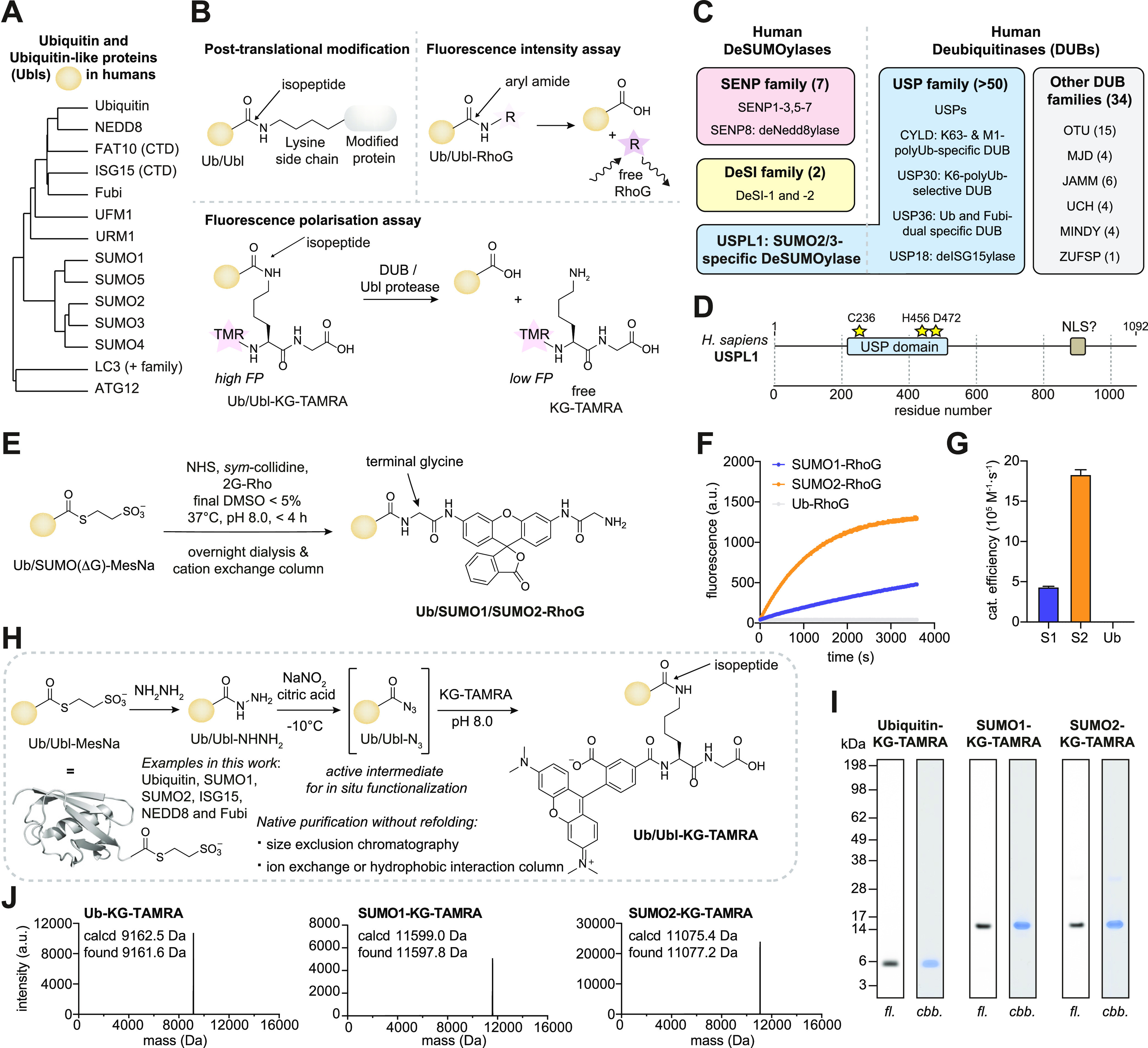
Substrate syntheses for activity measurements of deubiquitinases and deSUMOylases. (A) Average distance clustering of sequences of ubiquitin and ubiquitin-like (Ubl) modifier proteins. CTD, C-terminal domain. (B) Schematic of the isopeptide bond observed in post-translational Ubl modifications. Schematic of the fluorogenic Ub/Ubl-RhoG cleavage assay (top) and the Ub/Ubl-KG-TAMRA fluorescence polarization assay (bottom). (C) Human deSUMOylase and deubiquitinase enzyme families. The number of active family members is given in parentheses and members with specialized activities are given. USPL1 belongs to the Ubiquitin-specific protease (USP) family yet has SUMO-paralogue-specific deSUMOylase activity. (D) Domain architecture of human USPL1. The catalytic USP domain and the nuclear localization sequence (NLS) are shown as boxes, and residues of the catalytic triad as stars. (E) Synthesis and purification route for fluorogenic Ubiquitin/SUMO1/SUMO2-RhoG substrates. (F) Representative fluorescence over time trace ([USPL1] = 0.4 nM, [Ub/Ubl-RhoG] = 50 nM). (G) Catalytic efficiencies of USPL1 determined for indicated substrates as mean ± standard error. S1, SUMO1; S2, SUMO2; Ub, Ubiquitin. (H) General native synthesis and purification route for Ub/Ubl-KG-TAMRA substrates via acyl azide intermediates (this work, see Figure S1A for a comparison to previous work). (I) Gel-based analysis of indicated substrates; fl, fluorescence; cbb, Coomassie brilliant blue-stained. (J) Deconvoluted intact protein mass spectra of substrates shown in (I).

Ubiquitination acts as a highly versatile post-translational modification and regulates protein abundance, localization, and intracellular signaling in eukaryotic cells.^1−3^ Conjugation of the small protein Ubiquitin (Ub) through an isopeptide bond between the carboxylate of its C-terminal glycine and lysine side chains of substrate proteins is facilitated by an enzymatic cascade of E1, E2, and E3 enzymes, which can also catalyze the formation of polyubiquitin chains with different topologies as well as nonisopeptide-based ubiquitination.^1,2,4^ This system acts in parallel to various ubiquitin-like modifiers (Ubls) which share the ubiquitin fold but feature diverse sequences and thus mediate distinct processes (Figure 1A,B).^5^ Important examples for Ubls include NEDD8, whose attachment to Cullin Ring E3 ligases regulates their activity, ISG15, which mediates intracellular antiviral immunity, and various SUMO paralogues, which are involved in a plethora of cellular processes.^6^

Ub/Ubl protein conjugation can be reversed by specialized isopeptidases termed deubiquitinases (DUBs) or Ubl proteases, which antagonize Ub/Ubl-mediated post-translational modifications.^7,8^ Various members are currently being explored as therapeutic targets owing to their ability to stabilize proteins and as their inhibition amplifies Ub/Ubl-dependent signaling.^9^ There are approximately 100 DUBs known in humans which can be grouped into 7 different classes of which the ubiquitin-specific proteases (USPs) are the largest and most heterogeneous family (Figure 1C).^7^ While the bulk of USP DUBs are considered to be indeed ubiquitin-specific yet rather promiscuous with regards to the ubiquitinated substrates, some members notably feature preferences for distinct ubiquitin chains (CYLD^10^ and USP30^11^), display Ub/Ubl cross-reactivity (USP16 and USP36 for Ub and the Ubl Fubi;^12,13^ USP2, USP5, USP14 and USP21 for Ubiquitin and ISG15^14−16^), or are specific for a Ubl without ubiquitin activity (USP18 for ISG15,^17^ USPL1 for SUMO,^18^Figure 1D). Ub/Ubl cross-reactivity has also been observed in other DUB families (e.g., UCHL3 with activity for Ub and NEDD8^15^) and is particularly prevalent in viral and bacterial effector DUBs.^19−21^ Moreover, additional examples exist where a member of a particular Ubl protease fold has evolutionarily been co-opted to provide cleavage activity for a distinct Ubl. A notable case is NEDP1/SENP8,^22^ which features exclusive NEDD8 activity yet structurally belongs to the SENP family of deSUMOylases.^23^

There exist five SUMO Ubls in humans of which SUMO1 and SUMO2 are the most distantly related and best-studied paralogues.^24^ With only 45% sequence identity, these two Ubls share a lower sequence identity than, e.g., ubiquitin and NEDD8 (57%), and consequently nonredundant, paralogue-specific cellular roles have been described for SUMO1 and SUMO2.^24−26^ However, many mechanisms for paralogue specificity have remained poorly understood on the molecular level.

The ability to quantitatively assess SUMO paralogue specificity as well as Ub/Ubl cross-reactivity is important for the characterization of recombinant DUBs and Ubl proteases and thus their implication in biological pathways. Moreover, such activity assays can be used for the identification of inhibitors through high-throughput screening. Suitable substrates for in vitro assays comprise fluorogenic reagents (either as small peptides or the entire Ubl with a quenched fluorophore at their C-terminus^3,27^) as well as unlabeled isopeptide-linked Ubl protein substrates for gel-based assays which have both been used to characterize SENP deSUMOylases.^28^ Moreover, isopeptide-linked substrates (Ub/Ubl-KG-TAMRA, Figure 1B) for fluorescence polarization experiments have been reported, which are accessed through native chemical ligation with a δ-mercaptolysine-containing peptide, radical desulfurization, HPLC purification in organic solvents, lyophilization, and subsequent refolding in aqueous buffer (Figure S1A).^29^ While the latter typically works well for ubiquitin, non-ubiquitin Ubls are not only more difficult to synthesize^30^ but also refold less efficiently. This is apparent from assay data where the polarization signal upon complete conversion does not reach the level of free KG-TAMRA but stalls above, at times at about half the expected value, indicating that a significant portion of the substrate is not competent for enzymatic conversion (this is particularly pronounced for NEDD8 and SUMO substrates).^17,19,29^ These reagents have been invaluable for the enzymatic characterization of Ubl proteases,^29^ including PLpro of coronaviruses,^31^ and small-molecule inhibitor evaluation,^32^ yet comparisons across different Ubls and more widespread use are hampered by substrate heterogeneity. Moreover, their synthesis requires δ-mercaptolysine, which is not commercially available, and desulfurization can lead to cysteine to alanine mutations depending on the solvent accessibility of the thiol group.

To address these shortcomings, we developed individual synthesis and purification protocols for the generation of fully folded isopeptide-linked fluorescence polarization substrates based on Ubl C-terminal acyl azides. We confirm the complete conversion of a suite of Ub/Ubl-KG-TAMRA reagents in enzymatic assays and apply it to the quantification of Ub/Ubl cross-reactivity of various DUBs including the recently identified Fubi proteases USP16 and USP36,^12,13^ which we show to be the first trispecific Ubl isopeptidase with previously overlooked deISGylation activity. Moreover, we focused on recently structurally characterized USPL1^33^ (Figure 1D), which is the only deSUMOylase with a USP fold but for which its mechanism for SUMO paralogue specificity had remained unclear. Subsequent enzymatic, structural, and biochemical data reveal its mechanism for substrate specificity and also provide a rationale for the evolutionary adaptation of the USP fold into a deSUMOylase.

## Results

### Native Synthesis and Purification of Fluorogenic and Isopeptide-Linked Fluorescent Ub/SUMO Substrates

A preference for SUMO2-AMC over SUMO1-AMC was reported for USPL1 upon its discovery,^18^ but quantification of this paralogue specificity had not been performed. Due to the higher quantum yield of rhodamine dyes compared to coumarins, we synthesized fluorogenic Ub-, SUMO1-, and SUMO2-RhoG substrates starting from bacterially expressed and intein-mediated C-terminal Ub/Ubl thioesters and conversion through NHS-catalyzed aminolysis with bis-glycyl-rhodamine (Figures 1E and S2A, see Supporting Scheme 1 for rhodamine synthesis). Following straightforward purification by ion exchange due to the charge difference of product and starting material, these reagents were obtained in pure form (Figure S2B) and reported on a 4-fold higher catalytic efficiency in the catalytic domain of USPL1 for SUMO2-RhoG over SUMO1-RhoG (Figure 1F,G) in agreement with previous data with AMC substrates.^18^ Synthesis of SUMO fluorogenic substrates was previously reported using a fully Boc-protected protein in DMSO and with HPLC purification of intermediates.^34^ Our results indicate that fluorogenic SUMO reagents can also be directly obtained in pure form from SUMO C-terminal thioesters in aqueous reaction conditions in analogy to Ubiquitin-RhoG.^27^ Moreover, they demonstrate that their native state can be retained during purification by ion exchange^35^ instead of the previously employed reversed-phase chromatography, which circumvents organic solvents and refolding.

However, the chemical nature of the C-terminal aryl amide bond in these reagents is different from the physiologically relevant isopeptide in particular due to higher electrophilicity (Figure 1B). In order to determine enzymatic specificity with substrates of physiologically relevant linkage, we transitioned to SUMO-KG-TAMRA reagents,^29^ yet the aforementioned sample heterogeneity complicated quantitative analysis. Because aminolysis of C-terminal thioesters as used for the fluorogenic substrates would require concentrations of free KG-TAMRA peptide beyond its solubility limit in aqueous buffer, we surveyed the literature for milder protein chemical procedures for C-terminal protein functionalization.^36^ The in situ generation of acyl azides from C-terminal hydrazides with nitrous acid and the subsequent conversion into amides has been used for protein ligations and for the generation of probes for the Ubiquitin-activating enzyme^37,38^ and DUBs,^39^ yet had not been explored for substrates through Ubl functionalization. Adapting these chemical approaches, we converted C-terminal MesNa thioesters of Ub, SUMO1, and SUMO2 into the respective hydrazides, which proceeded quantitatively and without the necessity of purification (Figure S3A). We next subjected highly concentrated (0.5–6.0 mM) solutions of C-terminal hydrazides to nitrous acid at −10 °C for 2–10 min, which furnished hydrolysis-prone acyl azides. Subsequent incubation with 10–20 equiv of free KG-TAMRA at higher pH led to conversion into the respective substrates within 30 min (Figure 1H, see Supporting Schemes 2 and 3 for KG-TAMRA synthesis). For SUMO1, we observed partial nitrosylation, likely on its cysteine, which could be fully reversed through incubation with TCEP. Following optimization of conditions, this procedure featured the desired products in 20–40% yield (comparable to the native chemical ligation method), with various side reactions leading to complex protein mixtures including Ubl dimers, Ubl lactams, and Ubls with a carboxylate C-terminus. Owing to highly similar charge distributions of these species, yet very different sequence compositions and properties of the Ubls, individual purification procedures were scouted and optimized (Figure S1B). Ub-KG-TAMRA was obtained through cation exchange at pH 6 followed by cation exchange at pH 4.5. Owing to poor solubility at low pH, SUMO1-KG-TAMRA was purified through size exclusion chromatography followed by high-resolution anion exchange. SUMO2-KG-TAMRA was obtained through cation exchange and size exclusion chromatography. All reagents were obtained in pure form, as demonstrated by protein gels and intact protein mass spectrometry (Figure 1I,J, see the Supporting Information for mass spectrometry raw data). Traces of dimeric SUMO2-KG-TAMRA could be completely separated by an additional round of size exclusion chromatography (Figure S3B). Complete folding of substrates was assessed by circular dichroism (CD) spectroscopy (Figure S3C), which is in line with the standard use of low pH and high salt conditions during the purification and crystallization of folded Ubiquitin and Ubl species.^40^

### Revised USPL1-SUMO Paralogue Specificity in Isopeptidase Assays

**Figure 2:**
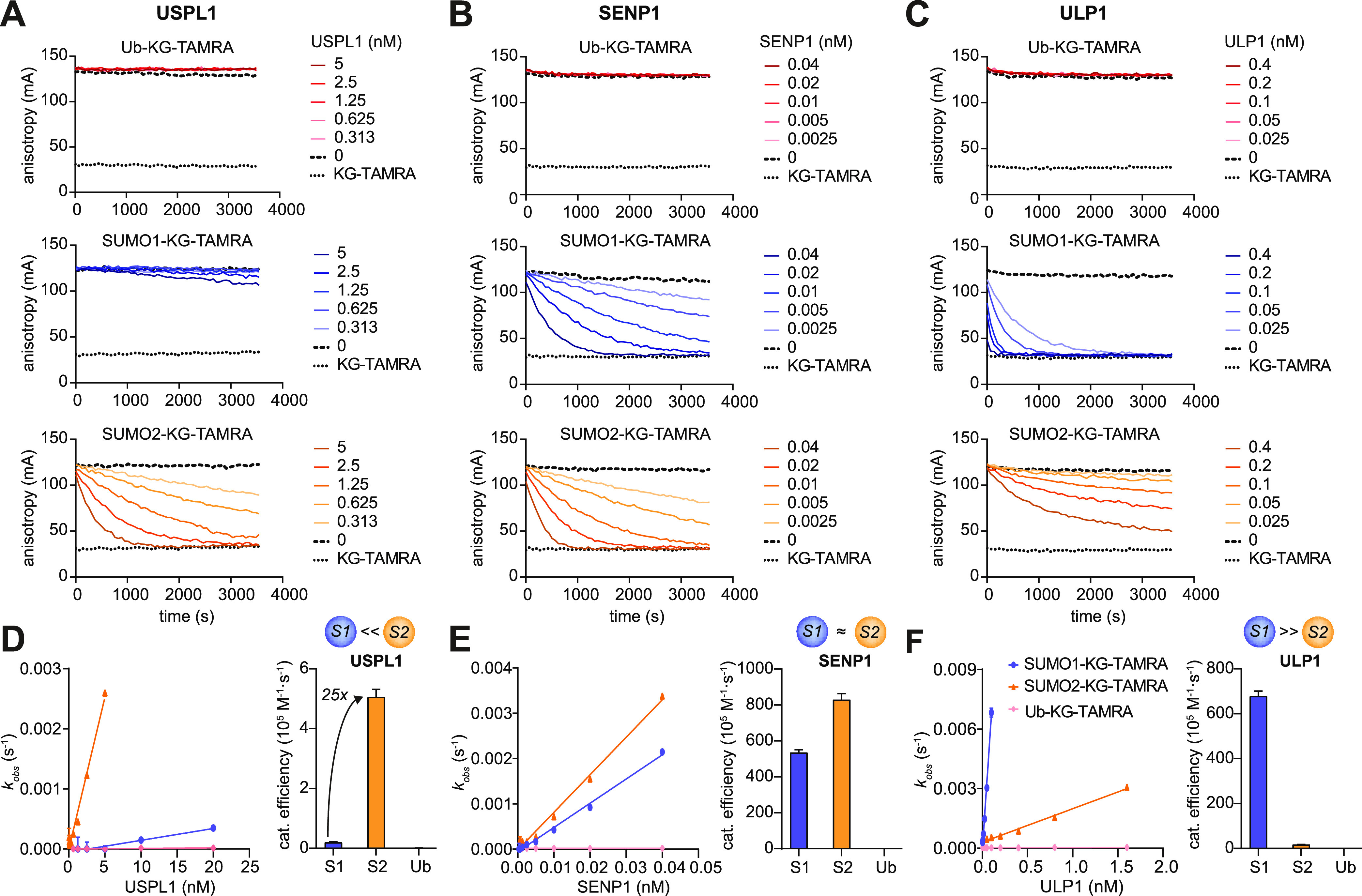
Assessment of SUMO paralogue specificity. (A–C) Fluorescence-polarization-based cleavage assays for indicated substrates and human USPL1 (A), human SENP1 (B), and yeast ULP1 (C). Averages of technical triplicates are shown, which are representative of three independent experiments. (D–F) Plots of observed rate constants over enzyme concentrations determined from assays shown in (A), (B) and (C) as well as Figure S2 (left). Catalytic efficiencies determined as slopes of *k*_obs_/[enzyme] plots are shown as bar graphs as the mean ± standard error (right).

We next normalized concentrations through fluorescence intensity measurements against free KG-TAMRA as a standard and tested the substrates in enzymatic assays with the DUB USP2 and the human deSUMOylase SENP1. We observed enzyme-concentration-dependent and complete cleavage of all substrates as demonstrated by the anisotropy levels of reactions reaching the same value as of free KG-TAMRA (Figures 2 and S3D,E). These data establish that all substrates were fully folded, as expected from the native preparation procedures, and that quantitative comparisons across Ubls are justified. We then profiled USPL1 which showed high activity against SUMO2-KG-TAMRA, but surprisingly much reduced conversion of the respective SUMO1 substrate (Figure 2A). This behavior is contrasted by the yeast deSUMOylase ULP1, which conversely displayed high activity against SUMO1, but not SUMO2 (Figure 2C). Measurements at broader concentration ranges allowed the determination of observed rate constants and thus catalytic efficiencies for all enzymes (Figures 2D–F and S3F–H). Since enzymes in the cellular environment typically operate under conditions of limiting substrates (i.e., [substrate] < *K*_M_), catalytic efficiencies (*k*_cat_/*K*_M_) are a viable metric for cellular enzymatic activity and the ratio of these efficiencies for substrate specificity. USPL1 featured an approximately 25-fold higher activity for SUMO2 over SUMO1 (Figure 2D), which suggests a much more pronounced paralogue specificity as previously assumed from fluorogenic reagents.^18^ Moreover, these data demonstrate the advantage of profiling substrate specificities of Ubl proteases with reagents containing physiologically relevant chemical linkage. The validity of the reagents is further supported by the observation that the only yeast SUMO protein SMT3 features a higher similarity to human SUMO1 than SUMO2, which is in line with the observed specificity of ULP1 (Figure 2C,F).

### Structural Basis for SUMO Paralogue Specificity in USPL1

**Figure 3:**
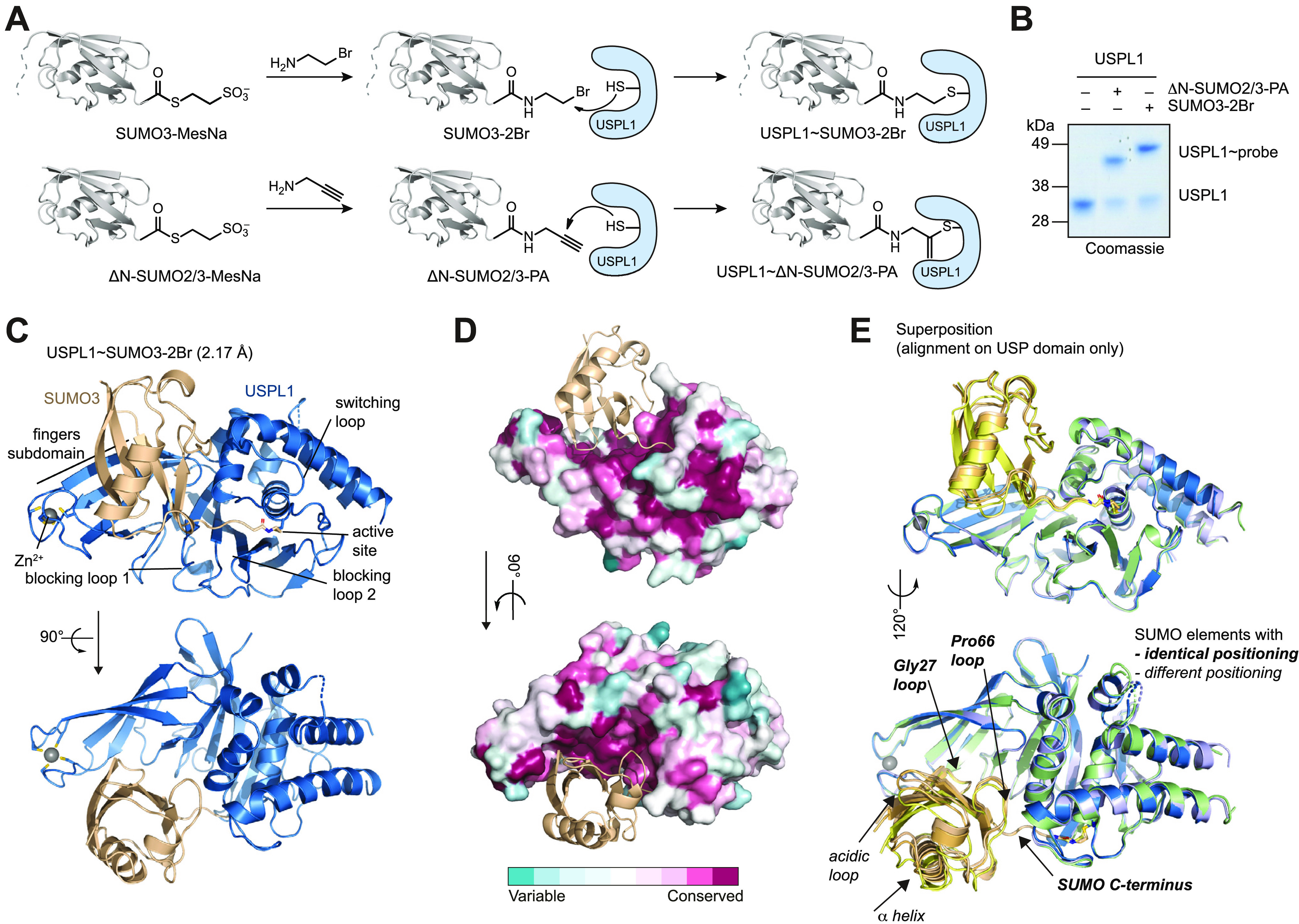
Recognition of SUMO by USPL1 relies on conserved interfaces with consistent relative positioning of SUMO across crystal forms. (A) Generation of substrate-trapped USPL1 complexes. The unstructured N-terminal sequence of SUMO is indicated by a dashed line. See Figure S4A for characterization data. (B) Coomassie-stained SDS-PAGE gel of the USPL1 catalytic domain reacting with indicated probes. (C) Cartoon representation of the crystal structure of USPL1 in complex with SUMO3–2Br. See Figure S5 for the asymmetric unit and density plots. (D) Sequence conservation was calculated from USPL1 orthologues as annotated by the Ensembl database and mapped as a colored surface on the structure shown in (C). (E) Superposition of the independent geometries of USPL1-SUMO complexes (see Table S1, colored as in Figure S5) with alignment on the USP domain. Regions of SUMO that contact USPL1 and whose relative positioning toward USPL1 is consistent across crystal forms are highlighted in bold.

To understand mechanistically how USPL1 discriminates between SUMO paralogues, we sought to structurally characterize its substrate recognition. Taking inspiration from Ubiquitin- and ISG15-processing USP-substrate complexes, we semisynthetically obtained SUMO activity-based probes that contain a reactive warhead at the protein’s C-terminus. SUMO2 and SUMO3 feature identical Ubl folds and differ only in their unstructured N-terminal extensions. We prepared a SUMO3–2Br probe with a 2-bromoethyl warhead as well as a ΔN-SUMO2/3-PA probe equipped with a propargylamine warhead^41^ (Figures 3A and S4A). Both probes reacted covalently with USPL1 as evident from a shift in molecular weight (Figure 3B) and led to pronounced protein stabilization as assessed by protein melting temperature analysis (Figure S4B,C), indicative of the specific recognition of SUMO by USPL1. In contrast to a recently reported SUMO-dehydroalanine probe^33^ which reacted with USPL1 but not a SENP deSUMOylase, the ΔN-SUMO2/3-PA probe reacted with both USPL1 and SENP1, but not the DUB USP2 (Figure S4D). Reactivity toward these probes thus paralleled enzyme activity and is in agreement with probe versions obtained through solid-phase-based chemical synthesis.^30^

We solved crystal structures of USPL1 in covalent complex with ΔN-SUMO2/3-PA to 2.4 Å resolution, as well as of USPL1 in complex with SUMO3–2Br to 2.17 Å resolution in a different crystal form (Table S1). The latter structure featured two almost identical copies in the asymmetric unit and was used for single-wavelength anomalous dispersion measurements with a selenomethionine-containing SUMO3–2Br probe (Figure S5A–C, Table S1). Both structures yielded well-defined electron density of all regions and allow unambiguous interpretation of the geometric arrangement (Figures 3C and S5C–G). Alignment of the catalytic triad Cys236, His456, and Asp472 in the SUMO3–2Br probe-bound structure was observed, and the importance of the respective residues for enzymatic activity as well as probe reactivity was confirmed by mutation (Figure S5H–J). The relative positioning of SUMO and USPL1 as well as unique features of the USPL1 fold in comparison to other Ubiquitin-processing USP family members are in full agreement with the recently published structure^33^ of USPL1 by the Reverter lab, which also explained why USPL1 does not process Ubiquitin. We confirm the importance of several residues around the palm subdomain, fingers subdomain, and coordination of the SUMO C-terminus, which are distinct from other USP family members (Figure S6A,B), for SUMO binding (Figure S6C) as well as substrate turnover (Figure S6D,E).

However, how USPL1 achieves its pronounced SUMO paralogue specificity (Figure 2A) is still unknown. In addition, why a USP DUB fold was evolved into a SUMO-processing enzyme distinct from the canonical SENP deSUMOylases has remained unclear. To address these questions, we first established whether USPL1 in addition to catalysis also binds to SUMO2 preferentially. To this end, we utilized the Ubl-KG-TAMRA reagents in a fluorescence polarization binding assay with catalytic cysteine-mutated protein version. USPL1^C236A^ specifically bound SUMO2-KG-TAMRA as is evident from a concentration-dependent increase in fluorescence anisotropy but did not bind SUMO1- or Ub-KG-TAMRA (Figure S6F). This behavior was contrasted by USP21^C221A^, which bound only the Ubiquitin reagent, thus proving the validity of the reagents prepared in the native fold also for substrate binding assays. Moreover, these data suggest that SUMO paralogue specificity of USPL1 in catalysis is based on specific recognition of SUMO2 over SUMO1. We therefore examined the interaction of SUMO and USPL1 in more detail.

A curated sequence alignment of USPL1 sequences of 183 organisms suggests that recognition of SUMO by USPL1 is based on a highly conserved patch in the finger subdomain, while other surface areas, with the exception of the surroundings of the catalytic center, are much more variable (Figure 3D). Alignment of three independently obtained relative arrangements of the USPL1 and SUMO folds revealed near-identical positioning of SUMO in these highly conserved areas including the SUMO C-terminus, the Pro66 loop, as well as the Gly27 loop in the fingers (Figure 3E). Recognition of the SUMO fold through the Gly27 loop is in striking contrast to Ubiquitin and ISG15 recognition by other USP enzymes which use a hydrophobic residue (Phe4 in Ub) as well as a large, complementarily shaped surface for many water-mediated interactions.^42^ SUMO regions not involved in direct USPL1 contacts such as the acidic loop and the α helix showed a different relative positioning (Figure 3E).

**Figure 4:**
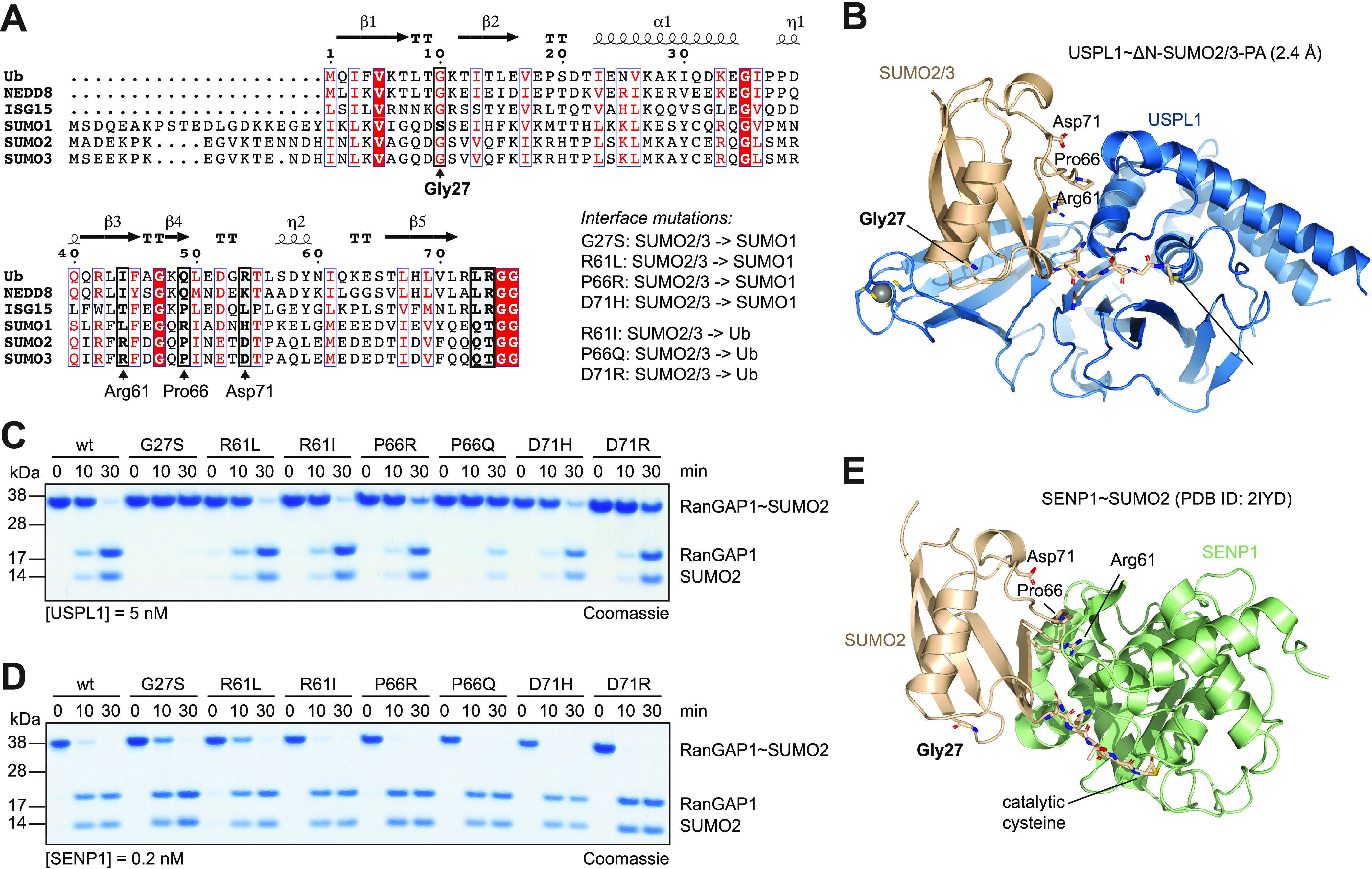
Structural basis for SUMO2/3 paralogue specificity in USPL1. (A) Alignment of the human Ub/Ubl sequences. Key residues of SUMO2/3 at the interface with USPL1 were selected for mutations into the corresponding amino acids in Ub or SUMO1 derived as shown. SUMO2 and SUMO3 differ in the unstructured N-terminal region; folded parts are identical yet differ by one in amino acid numbering. Residues are numbered according to the SUMO2 sequence throughout this work. (B) Key residues of SUMO2/3 involved in the interaction with USPL1 are highlighted in sticks. (C) Coomassie-stained SDS-PAGE gel of the isopeptide-linked RanGAP1∼SUMO2 cleavage assay with USPL1 for indicated time points. RanGAP1∼SUMO2 was assembled with mutations on SUMO2 with the rationale shown in (A). (D) RanGAP1∼SUMO2 cleavage assay with human SENP1 as in (C). (E) Structure of the SENP1∼SUMO2 covalent complex (PDB ID: 2IYD). Key residues interacting with USPL1 (see (B)) are highlighted in sticks.

We next compared SUMO paralogue sequences and designed mutations of residues in the USPL1 interface that converted SUMO2/3 residues to the respective SUMO1 or Ubiquitin residues (Figure 4A,B). To test their influence on substrate discrimination in USPL1, we assembled these mutated SUMO2 proteins as isopeptide-linked conjugates with RanGAP1, a commonly used SUMOylated protein (Figure S6G).^18^ We observed concentration-dependent turnover of the SUMO2 wild-type substrate by USPL1 (Figure S6H) and assessed all substrates at two time points (one for partial turnover and one for almost complete turnover). We observed that Pro66 (equivalent to Pro65 in SUMO3) and Asp71 play important roles in USPL1’s ability to discriminate against Ubiquitin as the P66Q and the D71R mutations were cleaved with a much reduced velocity. However, the equivalent residues did not discriminate against SUMO1 as substrates featuring the equivalent SUMO1 residues at these positions (P66R and D71H) were cleaved at similar rates as the wild-type substrate (Figure 4C). We next turned to Gly10 in Ubiquitin (Gly27 in SUMO2/Gly26 in SUMO3), where SUMO1 is the only Ubl featuring a serine at this position. Strikingly, mutation of Gly27 into serine abolished USPL1 activity completely (Figure 4C), in line with Gly27’s central structural role in anchoring of the SUMO fold into the USPL1 fingers (Figures 4B and S6A). In contrast, SENP1 cleaved all substrates completely (Figure 4D), including the G27S mutant, which is not contacted by the SENP fold (Figure 4E). These data demonstrate that the pronounced SUMO paralogue specificity of USPL1 toward SUMO2/3 over SUMO1 is based on USPL1’s ability to specifically recognize the Gly27 loop, as a serine at this position as in SUMO1 would disrupt the water-mediated interactions with USPL1 (Figure S6A). Moreover, this finding also provides a rationale for the evolution of a USP-fold enzyme into a deSUMOylase, as USPL1 can contact surface regions of the SUMO Ubl which are inaccessible through the SENP fold (Figure 4).

### Panel of Fully Folded Isopeptide-Linked Ub/Ubl-KG-TAMRA Substrates

**Figure 5:**
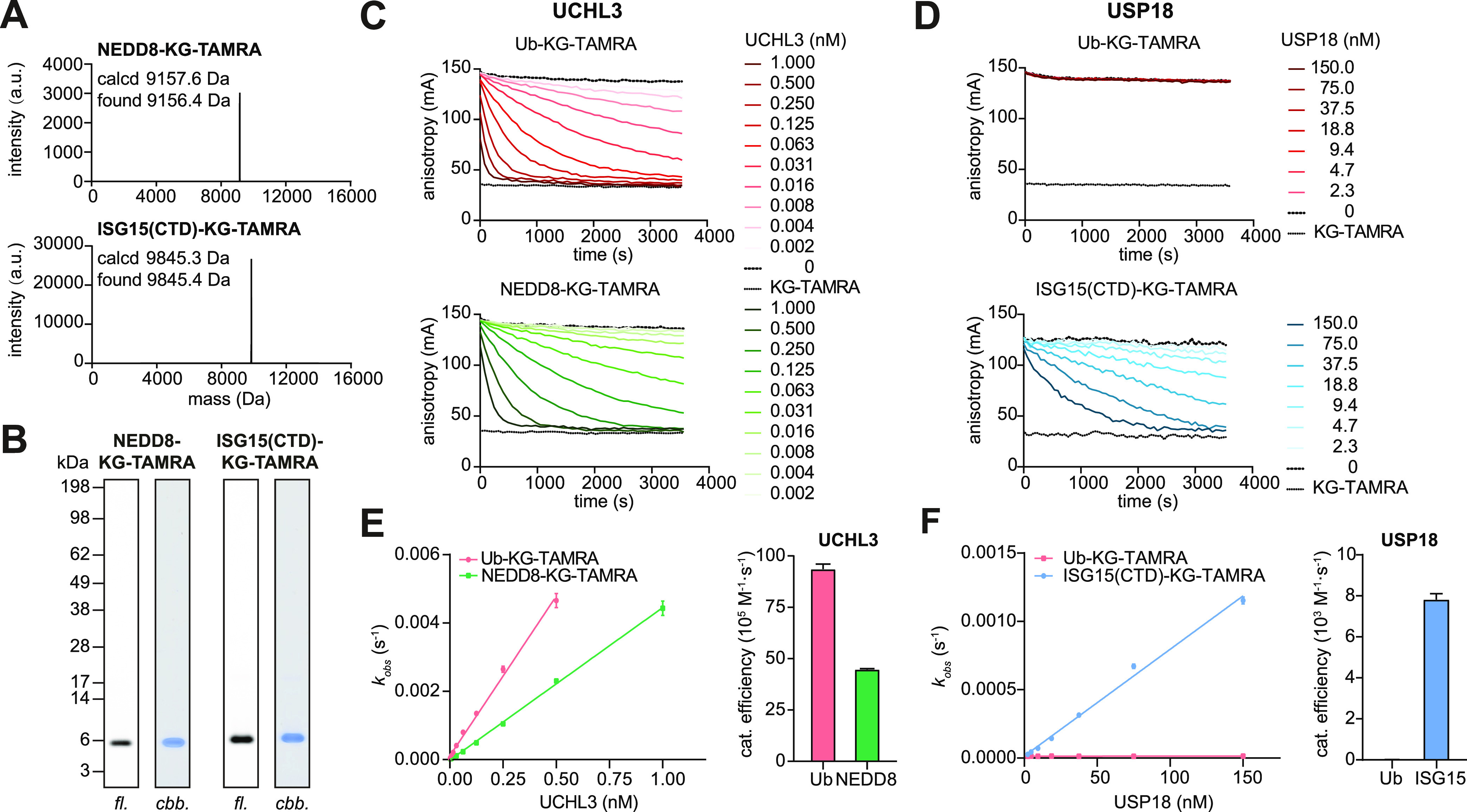
Preparation and cleavage of NEDD8 and ISG15 isopeptide substrates. (A) Intact protein mass spectra of NEDD8- and ISG15(CTD)-KG-TAMRA substrates which were prepared according to Figure 1H and purified according to Figure S7A. (B) Gel-based analysis of indicated substrates; fl, fluorescence; cbb, Coomassie brilliant blue-stained. (C, D) Fluorescence-polarization-based cleavage assays for indicated substrates and human UCHL3 (C) and human USP18 (D), shown as averages of technical triplicates representative of three independent experiments. (E, F) Plots of observed rate constants over enzyme concentrations determined from assays are shown in (C) and (D). Corresponding catalytic efficiencies are shown as mean ± standard error.

We were intrigued to see that a substrate preference observed from the semisynthetically obtained reagents could be structurally rationalized in the case of USPL1. In order to assess substrate specificity with isopeptide-linked reagents more broadly in Ubl proteases, we also prepared fluorescence polarization substrates for NEDD8 and the C-terminal domain of human ISG15 through the aminolysis of the respective Ubl C-terminal acyl azides. Both reagents were obtained in pure form (Figure 5A,B) following purification by size exclusion chromatography and cation exchange in the case of NEDD8-KG-TAMRA and hydrophobic interaction chromatography in the case of ISG15-KG-TAMRA (Figure S7A,C). We used the ISG15-specific protease USP18 (albeit the human enzyme in contrast to previously studied murine USP18)^17^ as well as the ubiquitin and NEDD8 cross-reactive DUB UCHL3^43^ for validation of the substrates (Figure 5C–F) and USPL1 as control (Figure S8A). We observed complete turnover of both substrates demonstrating their homogeneous folding (Figure 5C,E). Concentration-dependent measurement allowed the determination of catalytic efficiencies (Figure 5D,F). USP18 regulates interferon-mediated signaling through broadly antagonizing protein ISG15ylation,^44^ and its exquisite ISG15 specificity has been structurally characterized.^17^ Consistent with previous measurements of murine USP18 and murine ISG15 (63% sequence identity with human ISG15), we observed the turnover of only the ISG15 substrate by human USP18. The cross-reactivity of UCHL3 was previously investigated with fluorogenic Ub/NEDD8-AMC, which revealed a preference for ubiquitin over NEDD8 by 3 orders of magnitude.^43^ We confirm the preference for ubiquitin over NEDD8; however, we found the activity to isopeptide-linked ubiquitin and NEDD8 substrate in roughly the same range with drastically elevated NEDD8 activity compared to a partially convertible fluorescence polarization substrate.^29^ Analyzing the interaction of ubiquitin and UCHL3,^45^ we note that the majority of surface-exposed changes in NEDD8, which have been implicated in NEDD8-specific functions,^46^ do not contact UCHL3. The only difference is Arg72Ala, which would be consistent with the similar activity that we identify. These data show that a quantitative assessment of cross-reactivity of DUBs toward isopeptide-linked substrates can also lead to opposite effects than shown for USPL1, i.e., higher than previously recorded relative activity with fluorogenic reagents.

**Figure 6:**
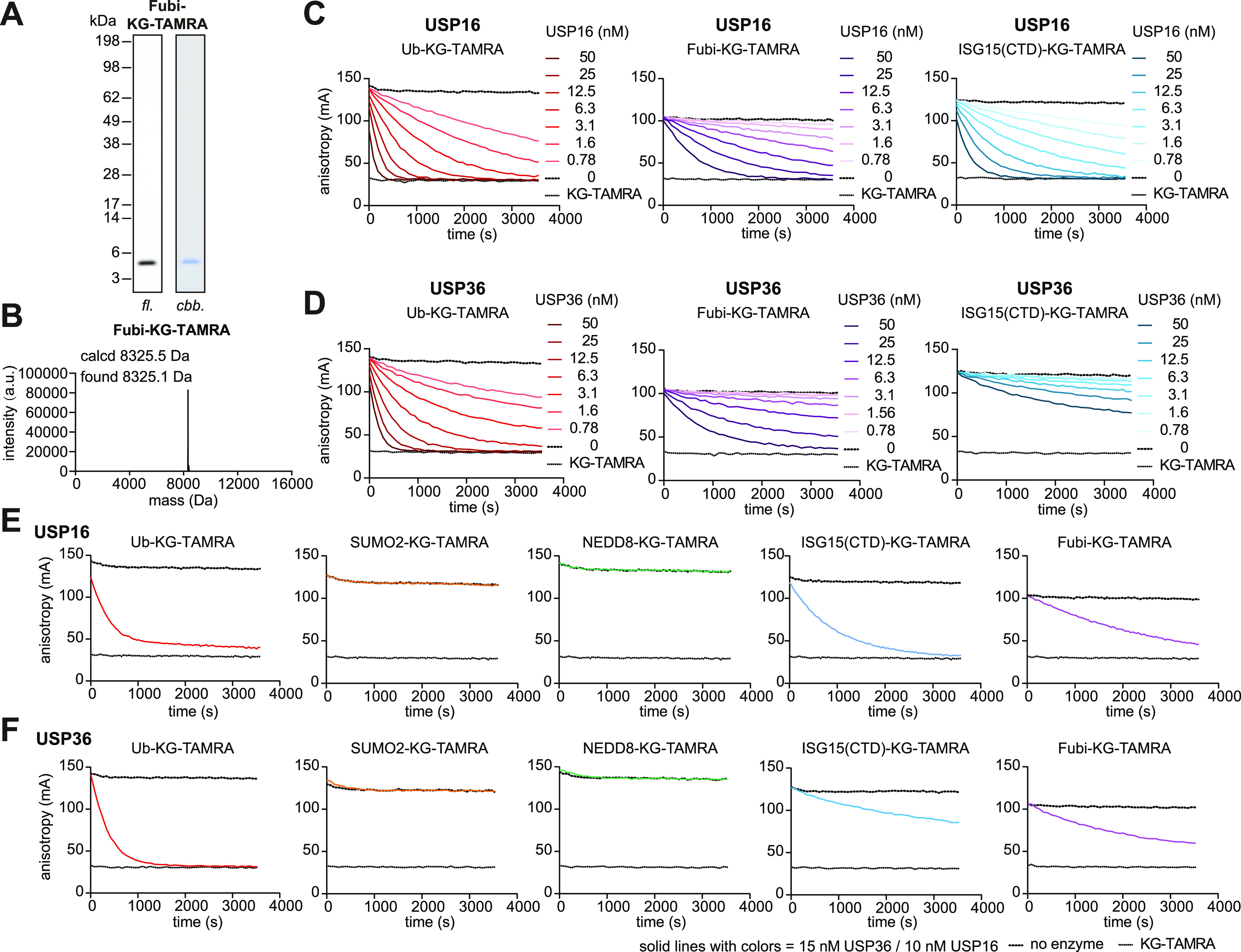
Isopeptidase cross-reactivity in USP16 and USP36 toward Ub, Fubi, and ISG15. (A) Gel-based analysis of Fubi-KG-TAMRA which was prepared according to Figure 1H and purified according to Figure S7B. fl, fluorescence; cbb, Coomassie brilliant blue-stained. (B) Intact protein mass spectrum of Fubi-KG-TAMRA. (C, D) Fluorescence-polarization-based cleavage assays for indicated substrates and human USP16 (C) and USP36 (D), shown as averages of technical triplicates representative of three independent experiments. (E, F) Fluorescence-polarization-based cleavage assays of the panel of Ub/Ubl-KG-TAMRA substrates and USP16 (E) and USP36 (F). See Figure S8 for USP2 and USP7 as controls as well as the catalytic efficiencies.

Next, we sought to extend the available substrate toolbox to investigate catalytic activity toward Ubls for which isopeptide-linked substrates had previously not been synthesized. We therefore turned to the Ubl Fubi which like ubiquitin is synthesized as an N-terminal fusion to a ribosomal protein (Fubi-S30) and cleaved by the nucleolar DUB USP36 to release free Fubi and S30.^12^ In addition to USP36, the mainly cytosolic DUB USP16 has Fubi protease activity, which may give rise to a two-tier system of Fubi-S30 maturation.^13^ In immune cells, Fubi conveys immunosuppressive signaling, including suppression of the maternal immune system upon embryo implantation, and isopeptide-linked conjugates of Fubi have been observed for selected proteins (termed Fubiylation in analogy to Ubiquitylation).^47,48^ However, which enzymes can act as deFubiylases with the ability to specifically antagonize these isopeptide-linked Fubi conjugates is not known. We employed the method reported here and synthesized Fubi-KG-TAMRA through a Fubi C-terminal acyl azide. The substrate was purified by gel-filtration and high-resolution anion exchange chromatography (Figure S7B) and was obtained in pure form (Figures 6A,B and S7D). Its conversion by USP16 and USP36 led to a decrease in the bulk fluorescence polarization signal consistent with complete turnover (Figure 6C,D). Concentration-dependent measurements allowed the determination of catalytic efficiencies with activities of both USP16 and USP36 toward isopeptide-linked Fubi approximately 6- to 8-fold lower than ubiquitin, yet within the same order of magnitude (Figure S8B,C). This experiment demonstrated that both DUBs have the catalytic ability to cleave isopeptide-linked Fubiylation.

**Figure 7:**
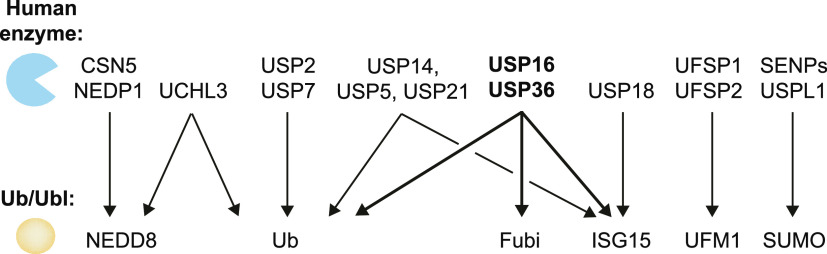
Cross-reactivity among human DUBs and Ubl proteases. Cross-reactivity is indicated with arrows. The here-identified trispecific isopeptidases USP16 and USP36 are highlighted in bold. Proteins studied in this work with a panel of isopeptide-linked reagents include USP2, USP7, USP16, and USP36. UCHL3, USP18, SENP1, and USPL1 were investigated with subsets of isopeptide-linked reagents.

Finally, we evaluated the ability of a selection of DUBs to cleave the full panel of fluorescence polarization substrates. To this end, we included both newly identified deFubiylases USP16 and USP36, the ISG15 cross-reactive DUB USP2 and the widely studied DUB USP7. Cross-reactivity of various DUBs toward ISG15 has previously been observed;^14−16,49^ however, this was mainly based on reactivity toward ISG15 activity-based probes and fluorogenic substrates. Our data show that recombinant USP2, while reacting with an ISG15 probe,^16^ cleaves the ubiquitin substrate selectively (Figure S8D). USP7 also exclusively cleaved the ubiquitin substrate (Figure S8E). However, to our surprise, we discovered that both USP16 and USP36 have, in addition to their ubiquitin and Fubi isopeptidase activities, also pronounced catalytic activity against ISG15-KG-TAMRA (Figure 6E,F). While USP36 showed only partial substrate turnover, USP16 displayed a complete turnover of ISG15-KG-TAMRA with a catalytic efficiency twice that of Fubi (Figures 6C,D and S8B,C). This strongly suggests that USP16 possesses previously unidentified roles in antagonizing both protein ISGylation and protein Fubiylation in the cytosol. Given its distinct localization (USP36 is the only active DUB localized to the nucleolus^50^), it is conceivable that USP36 antagonizes post-translational modifications of Ubiquitin, Fubi, and ISG15 in this organelle. Further work with separation-of-activity mutations will need to work out the cellular roles of the activities described here. Collectively, we identify USP16 and USP36 as the first human DUBs/Ubl proteases with activity toward three distinct Ub/Ubl substrates (Figure 7). These data highlight a surprising degree of substrate plasticity embedded in human DUBs and call for the validation of catalytic activities, which were discovered from Ubl activity-based probes, with isopeptide-linked substrates.

## Discussion

We here describe the facile semisynthesis and subsequent native purification of a panel of fully folded isopeptide-based fluorescence polarization substrates for DUBs and Ubl proteases. These substrates adopt a previously established design based on an isopeptide KG-TAMRA conjugate^17,19,29^ and were generated from easily accessible Ub/Ubl acyl azides and KG-TAMRA without the necessity of radical desulfurization, refolding, and commercially unavailable δ-mercaptolysine. We anticipate that a substrate panel obtained through this procedure will facilitate the quantitative characterization of further Ub/Ubl isopeptidase activities. Moreover, due to the complete enzymatic turnover and the thus increased change in bulk anisotropy, it will likely accelerate small-molecule inhibitor discovery through high-throughput screening. To the best of our knowledge, the use of substrates with endogenous linkage has not yet been reported in this context. Thus, the presented methodology provides a robust assay platform for DUB/Ubl protease-centered functional studies as well as drug development.

By comparing the catalytic efficiencies of the deSUMOylase USPL1 on fluorogenic substrates to those on isopeptide-linked variants, we discovered a much larger than previously assumed SUMO paralogue specificity. We structurally and biochemically explain this substrate selection, as USPL1 uses the USP fingers subdomain to contact the Gly27/Gly26 loop of SUMO2/3 to discriminate against SUMO1. This analysis not only defines a mechanism for SUMO paralogue specificity in USPL1 but also provides a rationale for the evolution of a USP-fold enzyme into a deSUMOylase, as this loop is not bound by the fold of SENP family deSUMOylases. Moreover, it cautions against the use of arylamide-containing substrates for inferring isopeptidase specificities.

Our findings of isopeptidase activities of USP16 and USP36 against the three distinct Ub/Ubl modifiers ubiquitin, Fubi, and ISG15 suggest that also other DUBs and Ubl proteases may have previously overlooked cross-reactivities toward isopeptide-linked post-translational modifications. We report that USP16 and USP36 are bone fide deFubiylases with catalytic activity against isopeptide-linked Fubi conjugates and that USP16 features an in vitro ISG15 isopeptidase activity at a similar if not higher level than USP18. It is foreseeable that the substrate panel can be expanded to further Ubls to examine the specificity of DUBs and Ubl proteases more broadly. While this work was being reviewed, a study was made available on a preprint server which identified ISG15 cross-reactivity of USP16, but not of USP36, through chemoproteomics.^51^ In a complementary manner, this manuscript extends the in vitro isopeptidase activity of USP16 toward full-length ISG15 and importantly demonstrates biological ramifications for the interferon-induced stimulation of protein ISGylation.^51^

We find it particularly noteworthy that an enzyme’s reactivity toward a Ubl activity-based probe does not necessarily translate into isopeptidase catalytic activity toward this Ubl. This can be seen from USP2, which reacts with an ISG15 probe but does not show catalytic turnover of the isopeptide-linked substrate. A similar observation was recently reported for USP5,^51^ which has been reported in several studies as ISG15 cross-reactive.^14−16^ We anticipate that understanding these discrepancies will give rise to improved workflows for the discovery of enzymatic activities. Collectively, these findings argue for the use of substrates with an endogenous linkage in the characterization of enzymatic activity and specificity.

The here-described bioconjugation procedure complements recently reported methodologies for the synthesis of Ub/Ubl isopeptide conjugates through native chemical ligation,^29^ photocatalyzed thiol–ene additions,^52^ as well as chemoenzymatic approaches relying on Ubc9,^53^ sortase,^54^ and asparaginyl endopeptidase.^55^ We expect that the C-terminal functionalization of ubiquitin and Ubls based on acyl azides coupled with native purification methods may also be extended toward the assembly of more complex substrates, activity-based probes, and nonisopeptide-linked substrates. This general chemical method is also complementary to a recently reported chemoenzymatic procedure which uses the viral Lb^Pro^ enzyme for the assembly of fluorogenic, but not yet isopeptide-containing substrates for ISG15, ubiquitin, and NEDD8 as per Lb^Pro^’s substrate spectrum.^14^ This method employs DMSO/water mixtures for fluorophore solubilization and subsequent protein refolding. While all methods have their unique strengths and limitations, together they will continue to expand the rich reagent toolbox, which is critical to comprehensively investigate DUBs and Ubl proteases, to enable therapeutic innovations, and to unravel important Ub- and Ubl-dependent biological processes.

## Supporting Information Available

Reaction schemes, purification procedures, intact protein mass spectra, kinetic data, biochemical assays, and protein structures (Figures S1–S8); data collection, phasing, and refinement statistics (Table S1); experimental procedures (Schemes 1–3); analytical data for all compounds including NMR spectra, uncropped gels, and supporting references (PDF)
Raw data of all intact protein mass spectra in measured and in deconvoluted forms (XLSX)
