# Supplementary material for: Native Semisynthesis of Isopeptide-Linked Substrates for Specificity Analysis of Deubiquitinases and Ubl Proteases
Source: J Am Chem Soc. 2023 Sep 15;145(38):20801–12. doi: 10.1021/jacs.3c04062 (PMC10540217; doi:10.1021/jacs.3c04062)
Supplement: Supplementary file 1 — ja3c04062_si_001.pdf [file ja3c04062_si_001.pdf]

Supporting Information

Native semi-synthesis of isopeptide-linked substrates for specificity analysis of deubiquitinases and Ubl proteases

Zhou Zhao[#,§], Rachel O'Dea[#,§], Kim Wendrich[#,§], Nafizul Kazi[#,§], and Malte Gersch[#,§,*]

[#] Max Planck Institute of Molecular Physiology, Chemical Genomics Centre,
Otto-Hahn-Str. 15, 44227 Dortmund, Germany

[§] TU Dortmund University, Department of Chemistry and Chemical Biology,
Otto-Hahn-Str. 15, 44227 Dortmund, Germany

* Correspondence: malte.gersch@mpi-dortmund.mpg.de

Contents:

1. Supporting Figures .... p. 2
2. Supporting Table .... p. 11
3. Experimental procedures .... p. 12
4. NMR spectra .... p. 29
5. Uncropped gels .... p. 37
6. Intact protein mass spectra .... p. 38
7. Supporting References .... p. 41

1. Supporting Figures

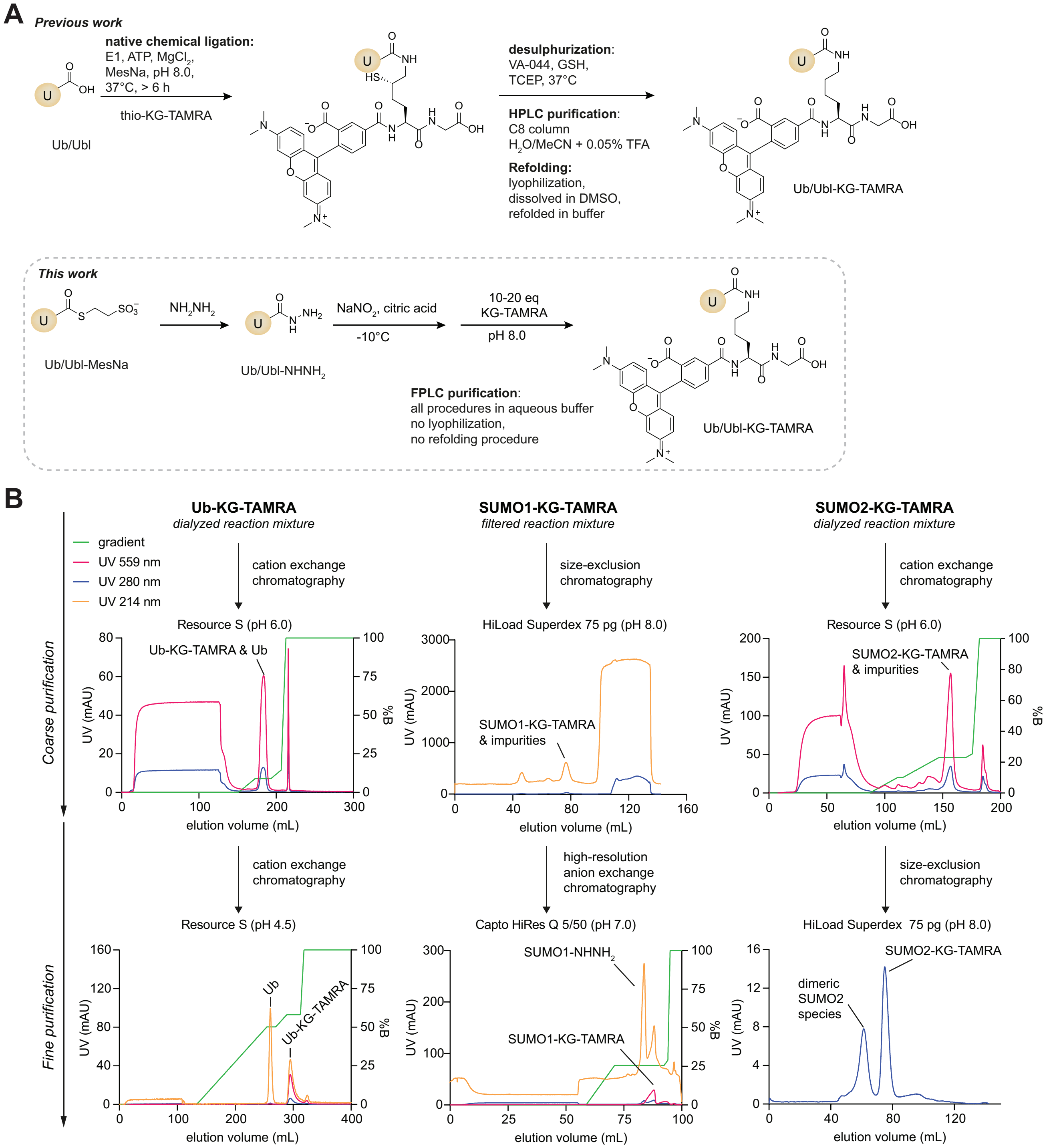

Figure S1. **Synthesis and purification of isopeptide-linked Ub/Ubl-KG-TAMRA substrates. A.** Comparison of previously[1] and here reported procedures for the preparation of fluorescence-polarization Ub/Ubl substrates. Key differences in reaction and purification conditions are highlighted. **B**. Native purification routes of Ub/SUMO1/SUMO2-KG-TAMRA reagents using protein chromatography methods in aqueous buffers. See the methods section for details. Species identified by intact protein mass spectrometry are indicated. The Ub/Ubl-KG-TAMRA substrates can be followed by their 559 nm UV absorbance.

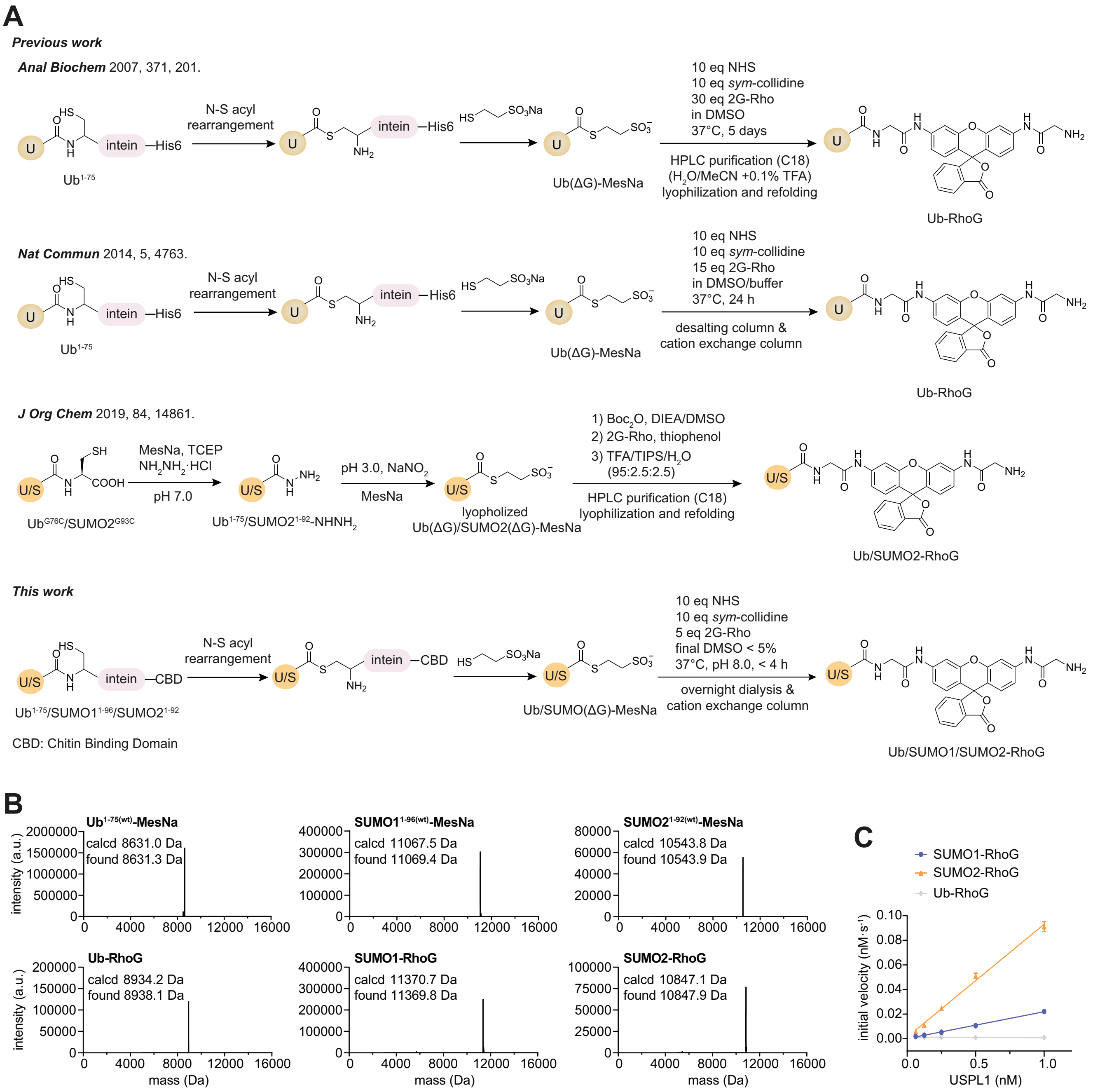

Figure S2. **Preparation of fluorogenic Ub/Ubl-RhoG substrates. A.** Comparison of previously[2-4] and here reported procedures for the preparation of Ub/SUMO1/SUMO2-RhoG substrates. Key differences in reaction and purification conditions are highlighted. **B.** Intact protein mass spectra of indicated Ub/Ubl thioesters and final fluorogenic RhoG substrates. **C.** Plot of initial velocities of indicated substrates (50 nM) and varying concentrations of USPL1. Slopes of this plot divided by the substrate concentration are plotted as catalytic efficiencies in Figure 1G.

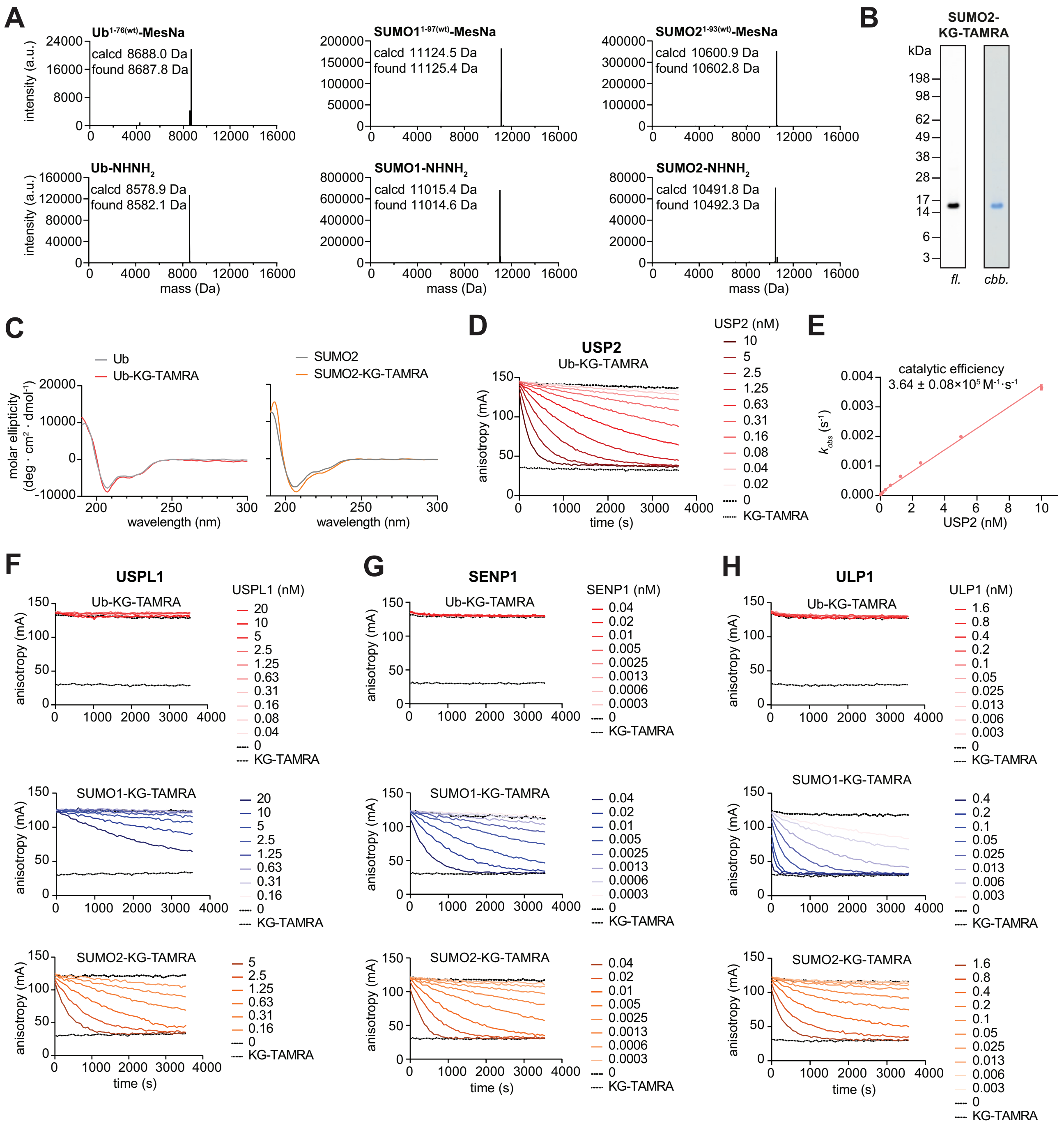

Figure S3. **Preparation and turnover of Ub/Ubl-KG-TAMRA substrates. A.** Intact protein mass spectra of indicated Ub/Ubl C-terminal thioesters and hydrazides. **B.** Gel-based analysis of SUMO2-KG-TAMRA after an additional round of gel filtration. fl, fluorescence. cbb, Coomassie brilliant blue-stained. **C.** Circular dichroism (CD) spectra of free Ubiquitin and Ubiquitin-KG-TAMRA (*left*) as well as free SUMO2 and SUMO2-KG-TAMRA (*right*), demonstrating complete folding of both substrates. **D.** Fluorescence-polarization Ub-KG-TAMRA cleavage assay of human USP2. Averages of technical triplicates are shown, representative of three independent experiments. **E.** Plot of observed rate constants over USP2 concentrations determined from the assay shown in D. The catalytic efficiency is given as mean ± standard error. **F.**,**G.**,**H.** Full concentration range of fluorescence polarization-based cleavage assays for indicated substrates and human USPL1 (F), human SENP1 (G) and yeast ULP1 (H) of Figure 2D-F.

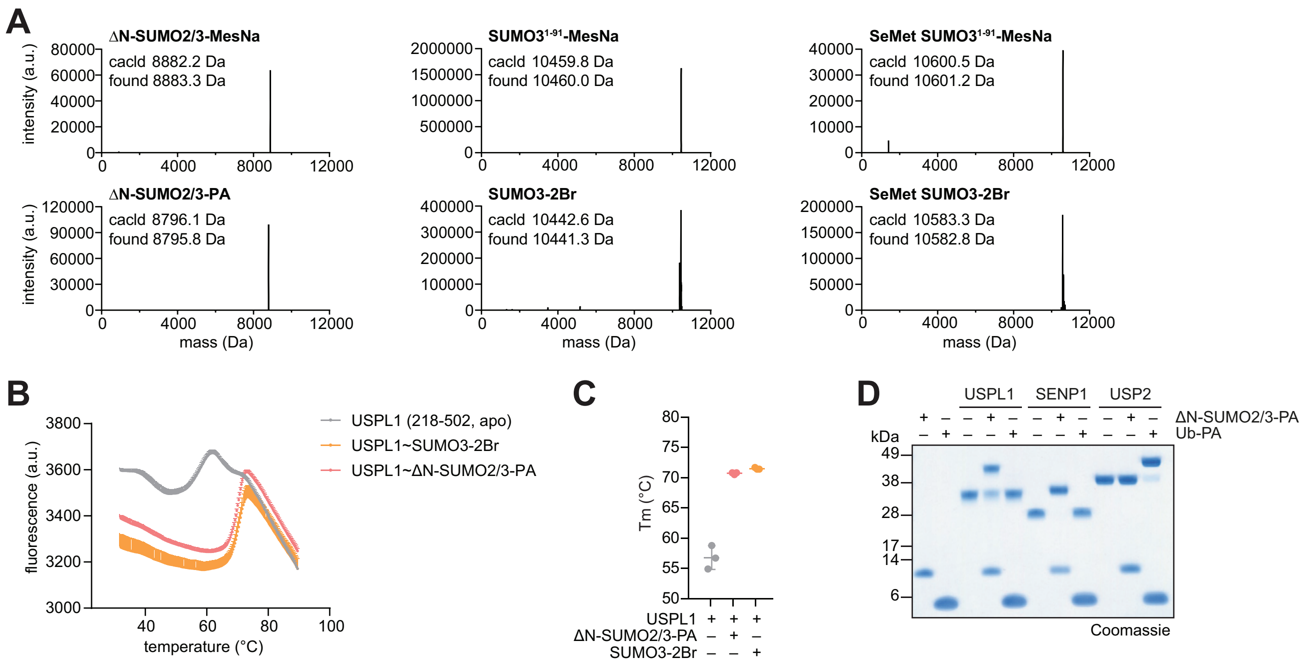

Figure S4. **Preparation and application of SUMO-containing activity-based probes. A.** Intact protein mass spectra of indicated SUMO C-terminal thioesters and activity-based probes. PA, propargylamine; 2Br, 2-bromoethylamine; SeMet, selenomethionine-containing protein. 2Br-functionalized probes are prone to hydrolysis as obvious from the also observed 2-hydroxyethyl species in all samples which are however not reactive towards enzymes. **B.** Representative curves of thermal shift assays from samples shown in Figure 3B. Protein melting temperatures were determined from inflection points, indicating large stabilization of USPL1 upon substrate engagement. **C.** Melting temperatures of indicated protein samples from three technical replicates. **D.** Ub/Ubl protease probe binding assay. Indicated proteins were reacted and samples were analyzed via Coomassie-stained SDS-PAGE.

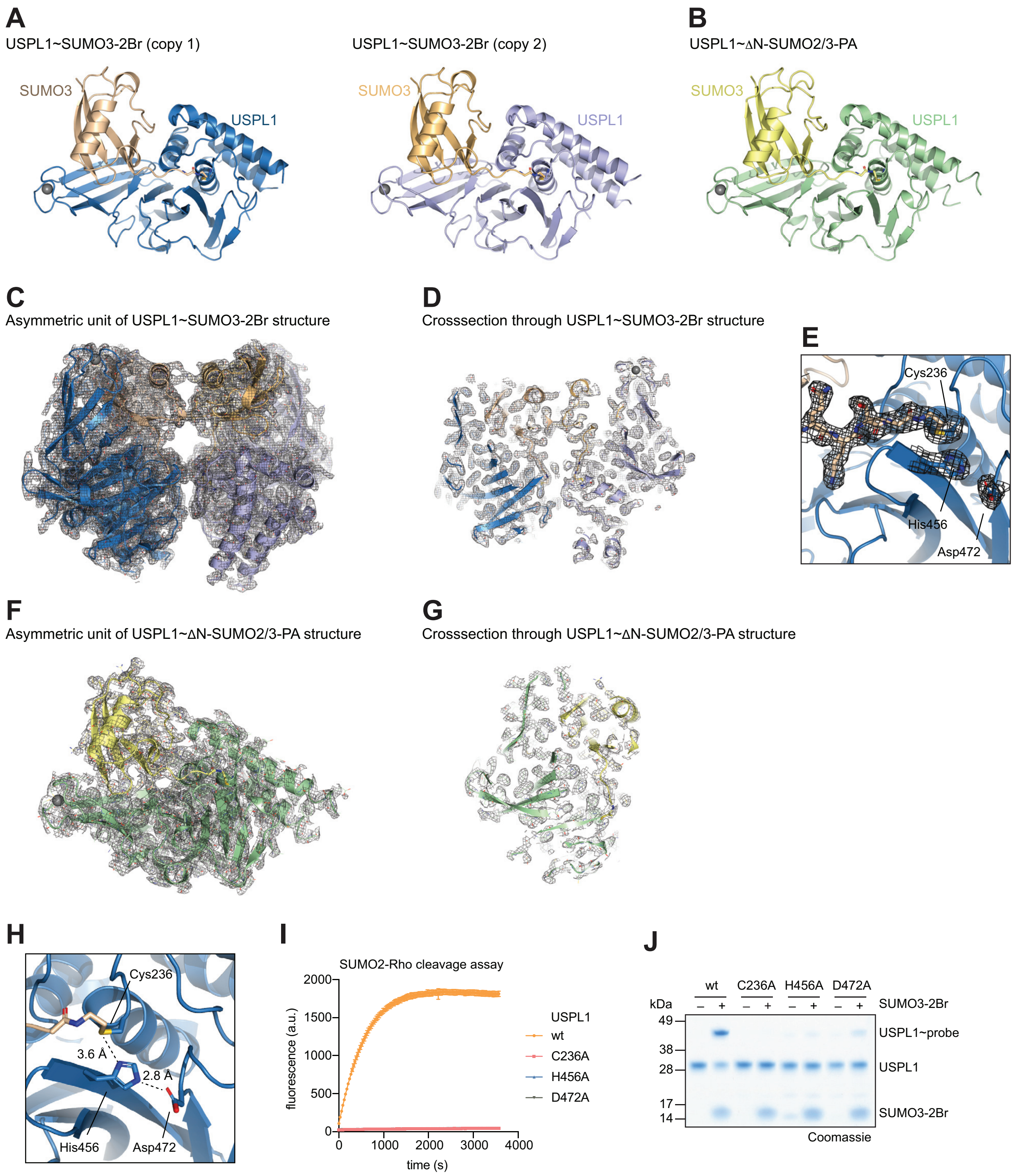

Figure S5. **Crystal structures of USPL1 in covalent complexes with SUMO2/3. A.** Cartoon representation of both copies in the asymmetric units of the USPL1~SUMO3-2Br structure. Zinc atoms are shown as grey spheres. **B.** Cartoon representation of the asymmetric unit of the USPL1~ΔN-SUMO2/3-PA structure. **C.** Electron density map of the asymmetric unit of the USPL1~SUMO3-2Br structure shown as the weighted $2|F_O|-|F_C|$ density contoured at $\sigma = 1.0$. **D.** Cross section through the density shown in C, centered on the SUMO C-terminal residues. **E.** Close-up view of the active site and the four most C-terminal residues of SUMO3-Br. Density for selected

residues as in C. **F.** Electron density map of the asymmetric unit of the USPL1~ΔN-SUMO2/3-PA structure shown as in C. **G.** Cross section through the density shown in F, centered on the SUMO C-terminal residues. **H.** Close-up view of the active site, showing alignment of the three residues of the catalytic triad. Hydrogen bonds are indicated with dashed lines, and atomic distances are given. **I**. SUMO2-RhoG cleavage assay with wild-type USPL1 or indicated mutants of the catalytic triad. An average of three technical replicates is shown ([USPL1] = 0.25 nM, [Ubl-RhoG] = 50 nM). **J.** Coomassie-stained SDS-PAGE gel of indicated USPL1 proteins, pre-incubated with SUMO3-2Br probe where shown.

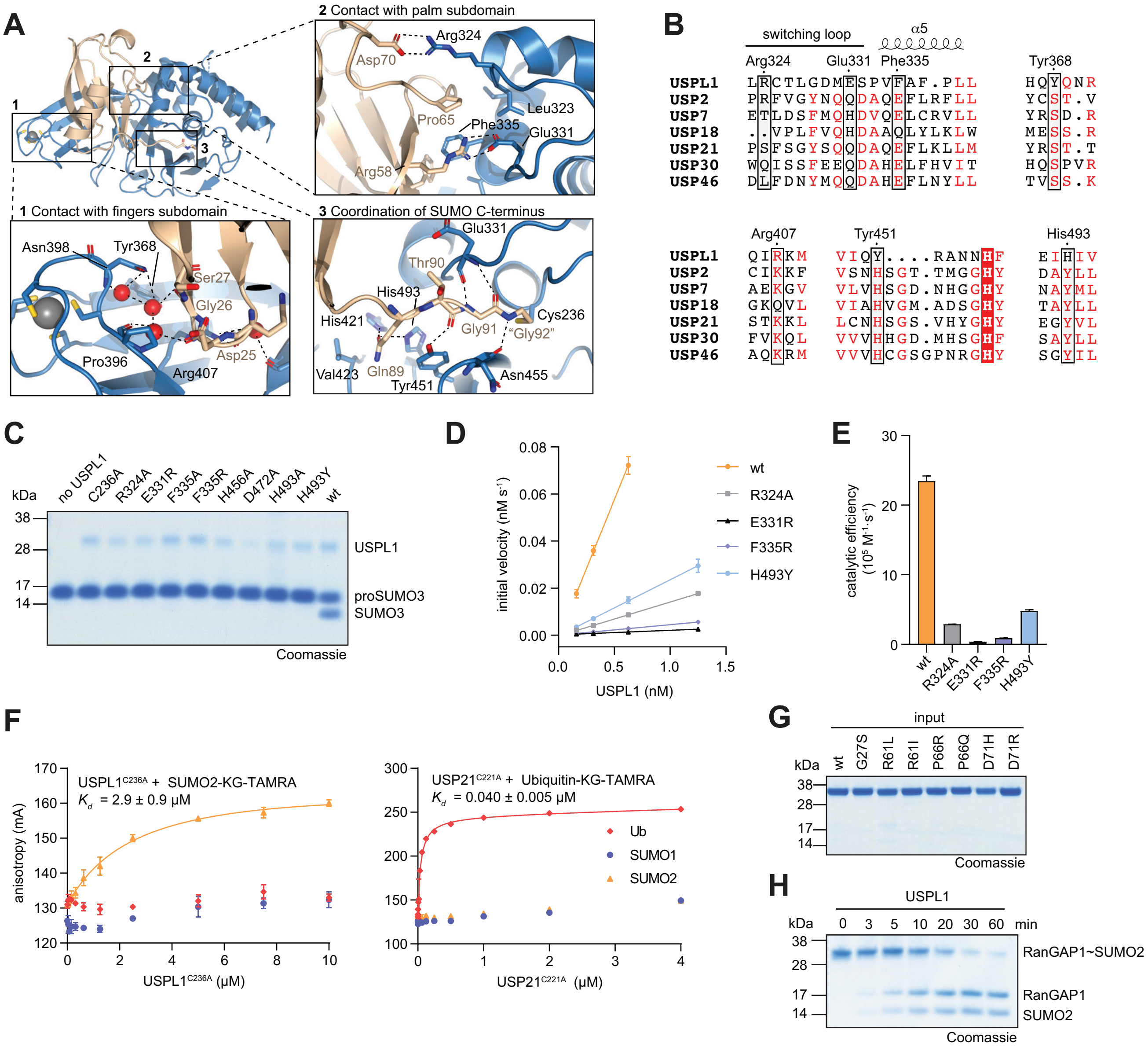

Figure S6. **Structural basis for SUMO2/3 recognition by USPL1. A.** Close-up view on three areas mediating SUMO2/3 recognition by USPL1, numbered according to small inserts, of the USPL1~SUMO3-2Br structure. Residues (sticks) and water molecules (red spheres) involved in the interaction between USPL1 and SUMO3 are highlighted. Hydrogen bonds are shown as dashed lines. **B.** Protein sequence alignment of USPL1 with other representative human USP DUBs. Key residues from areas important for SUMO interaction are highlighted in grey boxes. **C.** Coomassie-stained SDS-PAGE gel of proSUMO3 incubated with wild-type USPL1 or indicated mutants. **D.** SUMO2-RhoG cleavage assay using wild-type USPL1 or indicated mutants with different concentrations (N=3). **E.** Catalytic efficiencies calculated from D are shown as mean ± standard error. **F.** Fluorescence polarization binding experiment with catalytic cysteine mutated USPL1 (*left*) and USP21(*right*) as control and indicated Ub/SUMO1/SUMO2-KG-TAMRA tracers. **G.** Coomassie-stained SDS-PAGE of assembled RanGAP1~SUMO2 conjugates with mutations in SUMO2 as indicated. **H.** Coomassie-stained SDS-PAGE of a RanGAP1~SUMO2 cleavage assay with wild-type USPL1 and indicated time points.

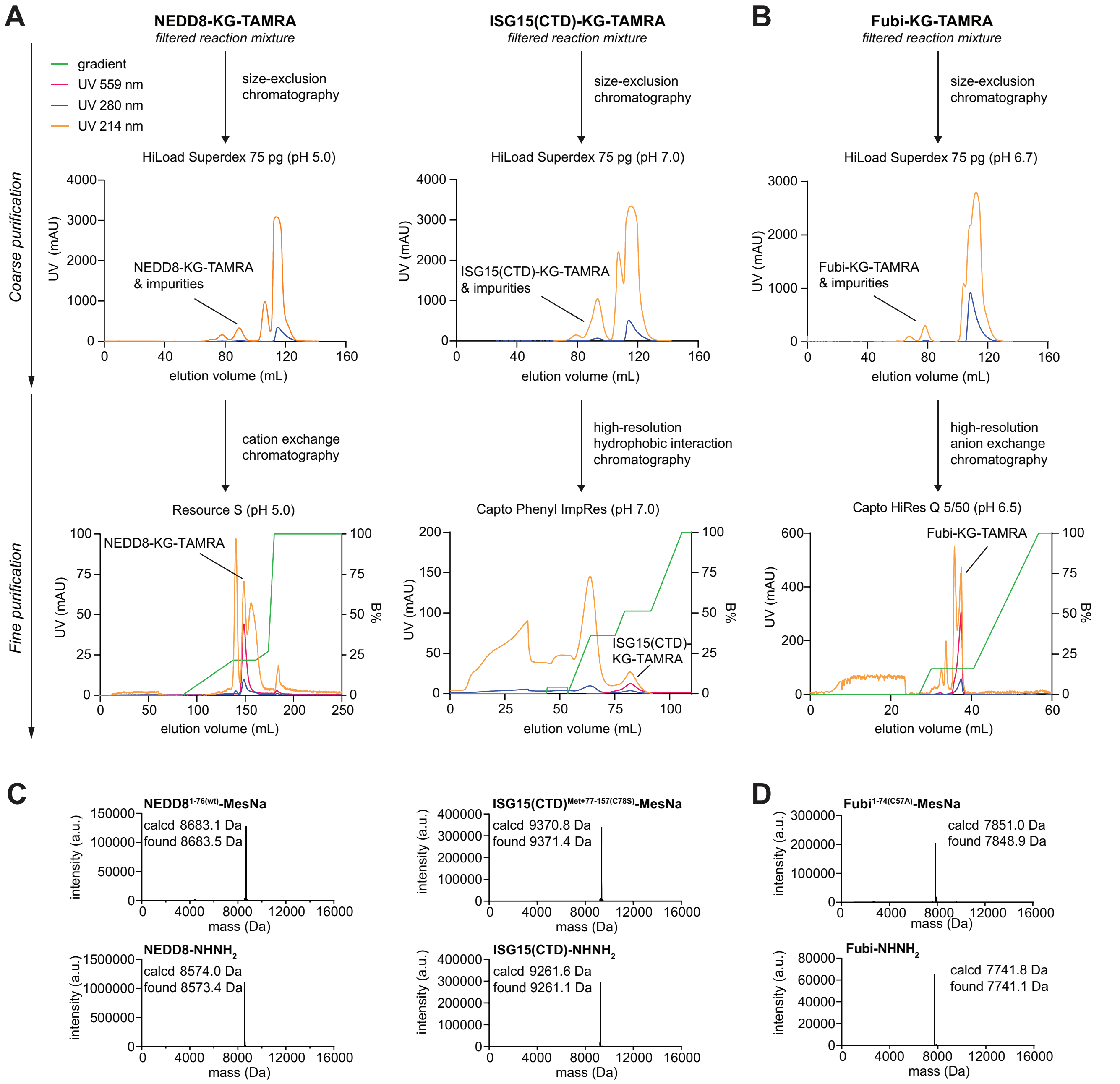

Figure S7. **Native purification of isopeptide-containing NEDD8, ISG15, and Fubi fluorescence polarization substrates. A.**,**B.** Purification routes for NEDD8- and ISG15(CTD)-KG-TAMRA (A) and Fubi-KG-TAMRA (B) using protein chromatography methods in aqueous buffers. See the methods section for details. Product-containing fractions are indicated and were confirmed by protein mass spectrometry. The Ub/Ubl-KG-TAMRA substrates can be followed by their 559 nm UV absorbance. **C.**,**D.** Intact protein mass spectra of C-terminal thioesters and hydrazides of NEDD8 and ISG15 (C) and Fubi (D).

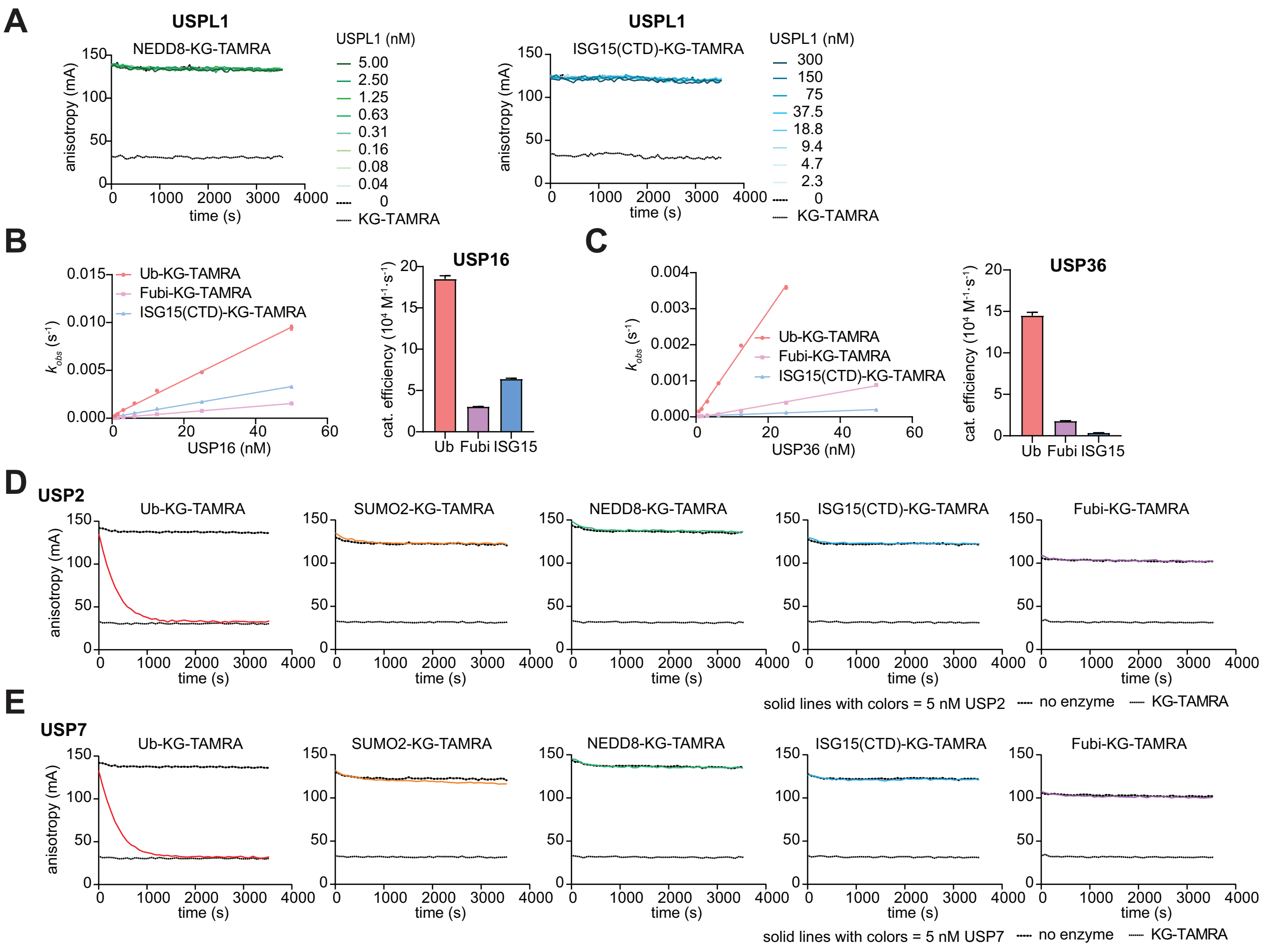

Figure S8. **Controls and cross-reactivity of human DUBs and Ubl proteases towards Ub/Ubls. A.** Fluorescence-polarization-based control cleavage assays for indicated substrates and human USPL1, shown as averages of technical triplicates representative of three independent experiments. **B.,C.** Plot of observed rate constants over USP16 (B) and USP36 (C) concentrations determined from assays shown in Figures 6C and 6D (*left*). Corresponding catalytic efficiencies are shown as mean ± standard error (*right*). **D.,E.** Fluorescence polarization-based cleavage assay of Ub/Ubl-KG-TAMRA substrate panel for USP2 (D) and USP7 (E), shown as in Figure 6E and 6F.

2. Supporting Tables

Table S1. Data collection, phasing and refinement statistics.

	USPL1~SUMO3-2Br (SeMet)	USPL1~SUMO3-2Br (PDB code: 7ZJU)	USPL1~ΔN-SUMO2-PA (PDB code: 7ZJV)
**Data collection**			
Beamline	SLS – PX3	SLS – PX2	SLS – PX2
Wavelength	0.9792 Å	1.000 Å	1.000 Å
Space group	$P\,2_1$	$P\,2_1$	$P\,4_1\,2_1\,2$
Cell dimensions			
a, *b*, *c* (Å)	66.84, 84.70, 71.58	66.06, 84.83, 71.11	95.71, 95.71, 82.88
α, β, γ (°)	90.0, 92.6, 90.0	90.0, 92.3, 90.0	90, 90, 90
Observed reflections	654,392	237,428	141,889
Unique reflections	24,690	41,570	15,670
Resolution (Å)	47.72 – 2.60 (2.72 – 2.60)	54.47 – 2.17 (2.25 – 2.17)	42.80 – 2.40 (2.49 – 2.40)
R_{merge}	0.221 (2.095)	0.112 (0.640)	0.053 (0.858)
R_{meas}	0.226 (2.149)	0.123 (0.711)	0.057 (0.933)
$I/\sigma(I)$	15.8 (1.7)	8.5 (2.4)	18.2 (1.8)
$CC_{1/2}$	0.999 (0.764)	0.996 (0.887)	0.999 (0.718)
Completeness (%)	100 (100)	99.9 (99.6)	100 (99.9)
Redundancy	26.5 (20.3)	5.7 (5.0)	9.1 (6.9)
Wilson *B* (Å^2)	42.3	33.8	79.3
**Phasing**			
Method	SAD	MR	MR
Resolution	2.6 Å		
Anom. completeness (%)	99.9 (99.8)		
Anom. multiplicity	13.5 (10.2)		
<FOM>	0.216		
**Refinement**			
Resolution		2.17 Å	2.40 Å
No. reflections		41,549	15,583
R_{work} / R_{free} (%)		19.2 / 23.8	23.0 / 25.8
No. atoms		5,527	2,659
Protein		5,166	2,621
Ligands		30	10
Water		331	28
B factors (Å^2)		45.3	110
Protein (Å^2)		45.0	110
Ligands (Å^2)		55.1	112
Water (Å^2)		49.5	103
R.m.s.d.			
Bond lengths (Å)		0.003	0.005
Bond angles (°)		0.64	1.13
Ramachandran (favored / allowed / outlier) (%)		97.2 / 2.8 / 0	97.0 / 3.0 / 0
Clashscore		3.5	6.0
Rotamer outliers (%)		0.6	1.5
Copies / a.s.u.		2	1

Each dataset was collected from a single crystal. Values in parentheses are for highest-resolution shells. Anom, anomalous; a.s.u., asymmetric unit; FOM, figure of merit; MR, molecular replacement; r.m.s.d., root mean square deviation; SAD, single-wavelength anomalous dispersion.

3. Experimental procedures

General notes on synthetic methods

Reagents and solvents were purchased from commercial sources and were used without further purification. LC-MS was measured on an Agilent 1260 UPLC coupled to an MSD mass analyzer equipped with an electrospray ionization (ESI) source. ^{1}H and ^{13}C NMR spectra were measured on Bruker AV 500 Avance III HD, AV 600 Avance III HD, Avance NEO 600 or AV 700 Avance III HD spectrometers. The chemical shifts of all spectra are specified in ppm and were referenced to the respective solvent peaks where possible. Thin-layer chromatography was carried out using silica gel aluminum plates (silica gel 60 GF254, Merck). Column chromatography was performed on silica gel (200 – 300 mesh). Preparative RPLC purification was performed using an 1260/1290 Infinity II series system (Agilent, for high pressure separation) or a Pure C-850 system (Büchi, for medium pressure separation) equipped with a VP125/21 Nucleodur C18 Gravity column (5 µm, Macherey Nagel) and solvents as specified (A: H_2O + 0.1% TFA, B: MeCN + 0.1% TFA). High resolution mass spectrometry data were measured on an LTQ Orbitrap XL mass spectrometer (Thermo Fisher).

Overview

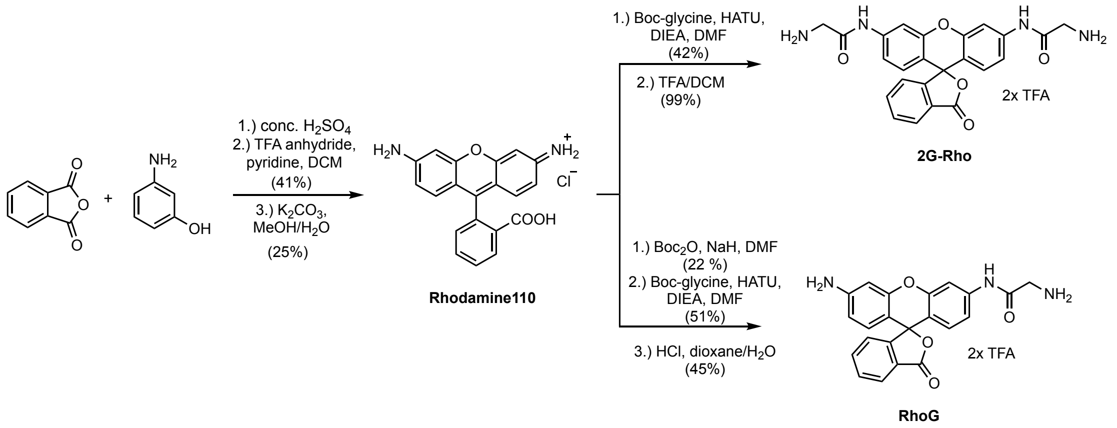

Supporting Scheme 1. Rhodamine110 was prepared as shown, and then converted into 2G-Rho for fluorogenic substrate generation (*upper path*) and into RhoG for signal normalization (*lower path*).

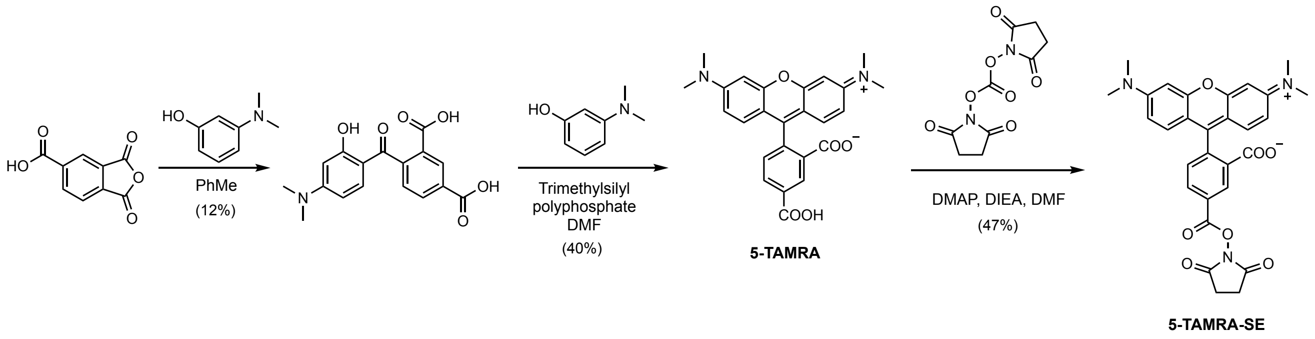

Supporting Scheme 2. Synthesis of 5-TAMRA and 5-TAMRA-SE.

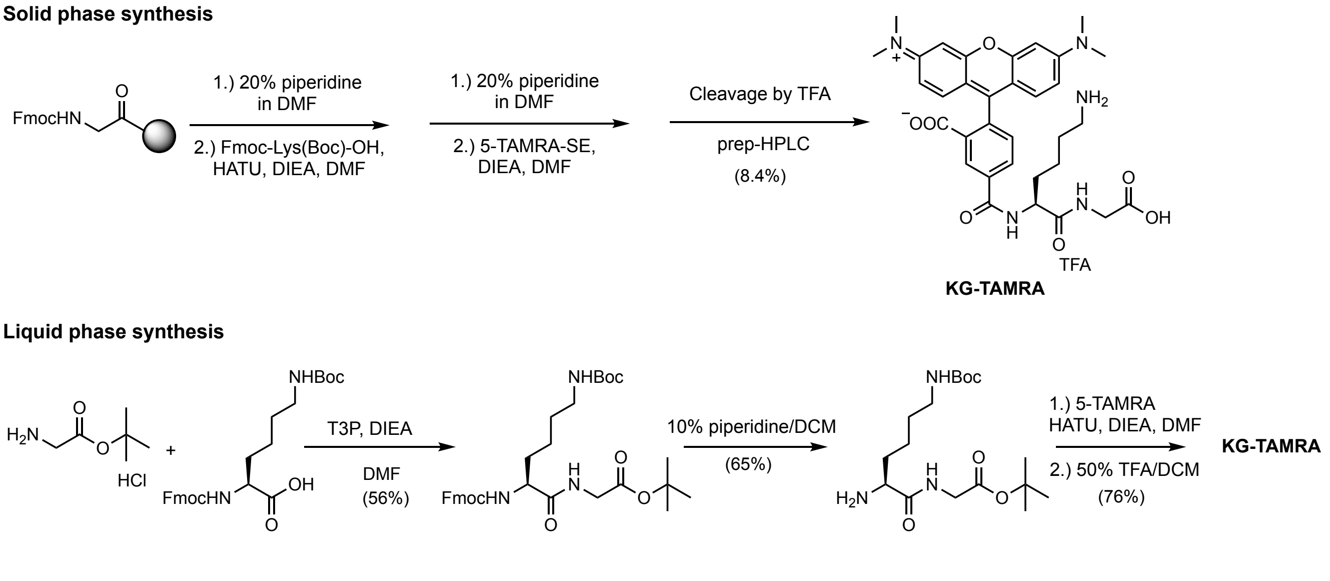

Supporting Scheme 3. Preparation of free KG-TAMRA for fluorescence polarization substrate generation. While the compound (referred to as TAMRA-Lys-Gly) has been referenced[1] and used widely[5-7], a procedure for its synthesis as well as characterization data to the best of our knowledge have not been reported. Two synthesis routes were explored: Through solid phase peptide synthesis in analogy to its 5*S*-methyldisulfanyl derivative[1] (*upper part*) as well as a solution phase route (*lower part*).

Rhodamine110

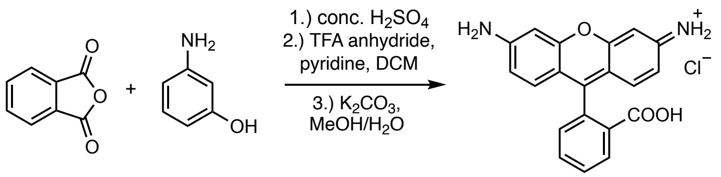

Synthesis of free rhodamine110 was performed according to a previously reported procedure[8] with some modifications kindly suggested by Luke Lavis (personal communication). In a round-bottom flask, phthalic anhydride (15.0 g, 101.2 mmol, 1.0 eq) and 3-aminophenol (18.8 g, 172.1 mmol, 1.7 eq) were dissolved in 75 mL concentrated sulfuric acid and heated at 180 °C for 5 h. Then the reaction mixture was poured into crushed ice, and subsequently diluted with water and concentrated ammonia solution. The mixture was kept at 4°C overnight and filtered. The filtrate was refluxed in 1 M HCl (400 mL) for 20 min and then hot filtered. The crude solution was allowed to stand at 4 °C for 40 h. The solid was filtered and dried in the oven to afford crude rhodamine110 as a black solid (27 g). To further purify rhodamine110, trifluoroacetic anhydride was used to protect the amines. To a solution of crude rhodamine110 (330 mg, 1.0 mmol, 1.0 eq) in dichloromethane (DCM), trifluoroacetic anhydride (0.524 mL, 3.8 mmol, 3.8 eq) and pyridine (0.3 mL) were added at rt, and it was stirred for 3 h. The reaction mixture was extracted with DCM, dried over $MgSO_4$, and purified by silica column using petroleum ether (PE) : ethyl acetate (EA) = 6:1 as eluant to give the product as an orange solid (200 mg, 41% yield). To a solution of TFA-protected rhodamine 110 (110.8 mg, 0.21 mmol, 1.0 eq) in methanol (MeOH), potassium carbonate (785.8 mg,

5.69 mmol, 27 eq) in 2 mL water was added and stirred overnight at rt. The reaction was then quenched with concentrated HCl and filtered. The filter cake was washed with cold water. The solid was dried at 60°C overnight to give a purple solid (20.8 mg, 25.4% yield). ESI-MS: *m/z* for $C_{20}H_{15}N_2O_3^+$ $[M+H]^+$ calcd 331.1, found 331.1; ^1H NMR (700 MHz, DMSO-*d*₆) δ 13.11 (s, 1H), 8.20 (d, *J* = 7.8 Hz, 1H), 8.06 (s, 4H), 7.85 (td, *J* = 7.5, 1.1 Hz, 1H), 7.78 (t, *J* = 8.2 Hz, 1H), 7.43 (d, *J* = 7.4 Hz, 1H), 7.04 – 6.91 (m, 2H), 6.90 – 6.73 (m, 4H).

Bis-BocGly-Rhodamine110

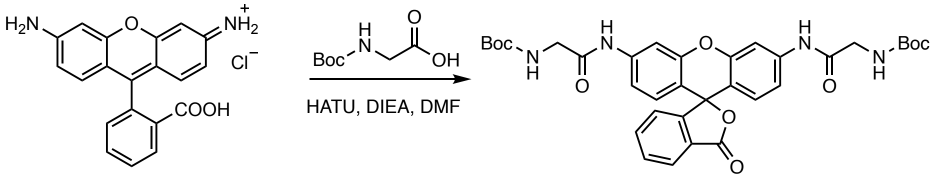

To a solution of Boc-glycine (524.4 mg, 3.0 mmol, 6.0 eq) were added HATU (1.14 g, 3.0 mmol, 6.0 eq) and DIEA (1.56 mL, 9.0 mmol, 9.0 eq) in DMF (5 mL), and it was stirred for 30 min. Then Rhodamine110 (183.0 mg, 0.5 mmol, 1.0 eq) was added and it was stirred overnight. The reaction mixture was diluted with 1 M HCl, extracted with EA and the combined organic phases were dried over $MgSO_4$. The filtrate was concentrated and purified on silica column (DCM:MeOH = 20:1) to give an orange solid (133.9 mg, 41.5% yield). ESI-MS: *m/z* for $C_{34}H_{37}N_4O_9^+$ $[M+H]^+$ calcd 645.3, found 645.2; ^1H NMR (500 MHz, DMSO-*d*₆) δ 10.26 (s, 2H), 8.03 (d, *J* = 7.6 Hz, 1H), 7.83 (d, *J* = 1.8 Hz, 2H), 7.82 – 7.77 (m, 1H), 7.73 (t, *J* = 7.4 Hz, 1H), 7.29 (d, *J* = 7.6 Hz, 1H), 7.17 (dd, *J* = 8.7, 2.0 Hz, 2H), 7.10 (t, *J* = 6.1 Hz, 2H), 6.75 (d, *J* = 8.7 Hz, 2H), 3.73 (d, *J* = 5.9 Hz, 4H), 1.39 (s, 18H).

Bis-Gly-Rhodamine110 (2G-Rho)

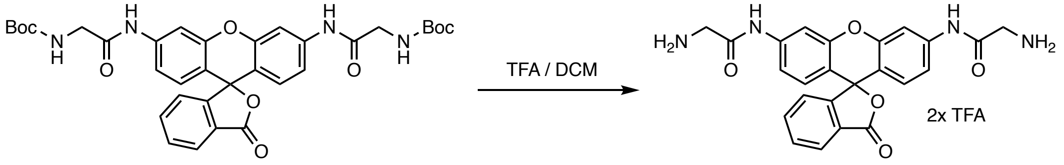

To a solution of Bis-BocGly-Rhodamine110 (506.5 mg, 0.79 mmol) in THF (10 mL), 50% TFA in DCM (15 mL) was added at 0°C. The reaction was moved to rt and stirred overnight. The solvent was evaporated to give the final product as an orange solid (660.5 mg, 99.2% yield). ESI-MS: *m/z* for $C_{24}H_{21}N_4O_5^+$ $[M+H]^+$ calcd 445.2, found 445.2; ^1H NMR (500 MHz, D_2O) δ 8.09 – 8.01 (m, 1H), 7.79 – 7.69 (m, 2H), 7.40 (d, *J* = 8.2 Hz, 2H), 7.17 (d, *J* = 6.5 Hz, 1H), 7.08 – 6.99 (m, 2H), 6.77 (dd, *J* = 10.4, 4.3 Hz, 2H), 3.94 (s, 4H).

mono Boc-protected Rhodamine110

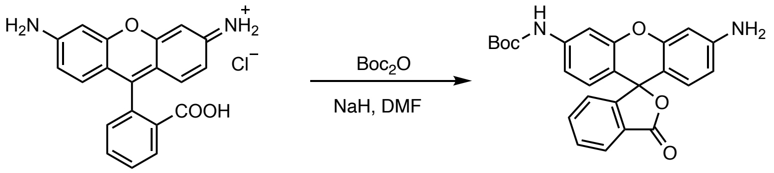

To a solution of Rhodamine110 (200 mg, 0.55 mmol, 1.0 eq) in anhydrous DMF (10 mL) was added NaH (60% in mineral oil, 43.6 mg, 1.1 mmol, 2.0 eq) slowly over a period of 5 min. The resulting mixture was stirred for 1 h, then di-*tert*-butyl dicarbonate (Boc_2O, 101.2 mg, 0.46 mmol, 0.84 eq) was added and the mixture stirred at ambient temperature for 2 days. The reaction was quenched with acetic acid (1 mL) and water (5 mL). The mixture was diluted with EA (50 mL) and washed with water and brine. The organic layer was dried over Na_2SO_4, filtered, and concentrated *in vacuo*. The crude product was purified by silica column (PE:DCM:EA = 4:4:2) to give an orange solid (52.7 mg, 22.3% yield). ESI-MS: *m/z* for $C_{25}H_{23}N_2O_5^+$ $[M+H]^+$ calcd 431.2, found 431.2; ^1H NMR (700 MHz, DMSO-d_6) δ 9.64 (s, 1H), 7.97 (d, *J* = 7.7 Hz, 1H), 7.78 (td, *J* = 7.5, 1.0 Hz, 1H), 7.73 – 7.68 (m, 1H), 7.51 (s, 1H), 7.23 (d, *J* = 7.7 Hz, 1H), 7.08 (dd, *J* = 8.7, 2.0 Hz, 1H), 6.58 (d, *J* = 8.7 Hz, 1H), 6.43 (d, *J* = 2.0 Hz, 1H), 6.36 (d, *J* = 8.6 Hz, 1H), 6.32 (dd, *J* = 8.6, 2.0 Hz, 1H), 5.65 (s, 2H), 1.48 (s, 9H).

Dual-Boc-protected Gly-Rhodamine110

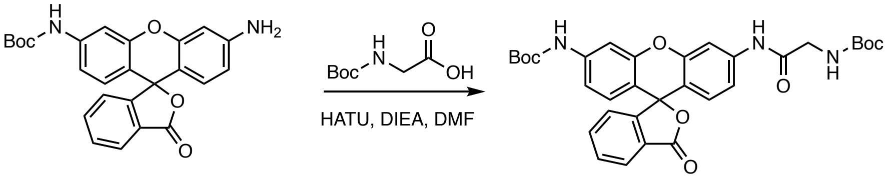

To a solution of Boc-glycine (36.6 mg, 0.21 mmol, 1.0 eq) in DMF were added HATU (106.0 mg, 0.28 mmol, 1.3 eq) and DIEA (0.49 mL, 0.28 mmol, 1.3 eq) and it was stirred for 30 min. Then mono Boc-protected Rhodamine110 (30.0 mg, 0.70 mmol, 3.3 eq) was added and stirred overnight. The reaction was extracted with EA and dried over $MgSO_4$. The filtrate was concentrated and purified on silica column (PE:EA = 1:1) to give an orange solid (21.0 mg, 51.2% yield). HRMS: *m/z* for $C_{32}H_{34}N_3O_8^+$ $[M+H]^+$ calcd 588.2340, found 588.2335; ^1H NMR (700 MHz, $CDCl_3$) δ 8.36 (s, 1H), 8.05 – 8.00 (m, 1H), 7.68 – 7.63 (m, 2H), 7.63 – 7.58 (m, 1H), 7.50 (s, 1H), 7.12 (d, *J* = 7.5 Hz, 1H), 7.04 (d, *J* = 8.4 Hz, 1H), 6.93 – 6.87 (m, 1H), 6.73 (d, *J* = 8.6 Hz, 1H), 6.69 (d, *J* = 8.6 Hz, 1H), 6.62 (s, 1H), 5.23 (t, *J* = 6.2 Hz, 1H), 3.93 (d, *J* = 6.1 Hz, 2H), 1.53 (s, 9H), 1.48 (s, 9H). ^{13}C NMR (176 MHz, $CDCl_3$) δ 169.68, 153.43, 152.49, 151.97, 151.89, 140.70, 135.27, 129.93, 128.80, 128.71, 126.47, 125.24, 124.05, 115.50, 114.35, 113.20, 108.00, 106.28, 82.69, 55.90, 29.85, 28.43.

Gly-Rhodamine110 (RhoG)

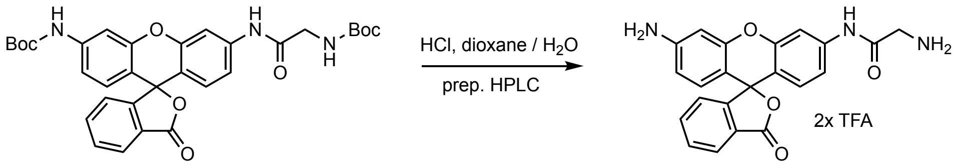

To a solution of dual-Boc-protected Gly-Rhodamine110 (17.0 mg, 0.29 mmol) in a round-bottom flask in 1,4-dioxane (1 mL), 3 mL HCl (4 M in dioxane/H_2O) was added and stirred overnight with protection from light. The solvent was evaporated *in vacuo*. The crude mixture was purified by reverse phase HPLC. Pure fractions were pooled and lyophilized to give RhoG as an orange solid (6.0 mg, 45.0% yield). HRMS: *m/z* for $C_{22}H_{18}N_3O_4^+$ [M+H]$^+$ calcd 388.1292, found 388.1296; ^{1}H NMR (600 MHz, D_2O) δ 8.19 – 8.15 (m, 1H), 8.15 – 8.10 (m, 1H), 7.82 – 7.78 (m, 2H), 7.45 (d, *J* = 8.9 Hz, 1H), 7.43 – 7.39 (m, 3H), 7.00 (dd, *J* = 9.3, 2.0 Hz, 1H), 6.92 (d, *J* = 1.9 Hz, 1H), 4.09 (s, 2H). ^{13}C NMR (151 MHz, D_2O) δ 172.33, 166.11, 163.12, 162.88, 161.25, 159.62, 154.65, 144.21, 133.21, 133.04, 131.54, 130.53, 130.44, 129.61, 129.57, 119.65, 118.06, 117.78, 117.28, 117.24, 115.35, 106.94, 97.82, 41.41.

4-(4-(Dimethylamino)-2-hydroxybenzoyl)isophthalic acid

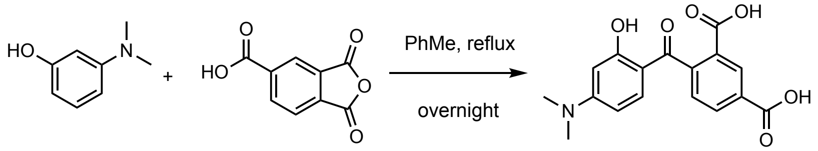

Synthesis of 5-TAMRA was based on previous literature[9]. To a solution of 3-dimethylaminophenol (6.86 g, 50.0 mmol, 1.0 eq) in toluene, 1,2,4-benzenetricarboxylic anhydride (11.53 g, 60.0 mmol, 1.2 eq) was added and refluxed overnight. Product formation was observed via TLC ($CHCl_3$:MeOH:TEA = 85:10:5, R_f = 0.36) consistent to literature[9]. The reaction was then cooled down and filtered. The filter cake was washed with toluene (50 mL×2), then dissolved in 200 mL MeOH and refluxed for 10 min. 50 mL acetic acid was added into the solution and evaporated under vacuum. The residue was dissolved in 100 mL MeOH and refluxed for 2 h and then moved to 4°C overnight. The reaction was filtered, and the filter cake was washed with cold MeOH to give the final product as a brown solid (1.94 g, 12% yield). The product was used without further purification. ESI-MS: *m/z* for $C_{17}H_{16}NO_6^+$ [M+H]$^+$ calcd 330.1, found 330.0. ^{1}H NMR (500 MHz, DMSO-*d*$_6$) δ 12.38 (s, 1H), 8.48 (d, *J* = 1.6 Hz, 1H), 8.22 – 8.14 (m, 1H), 7.50 (d, *J* = 7.8 Hz, 1H), 6.80 (d, *J* = 9.1 Hz, 1H), 6.21 (dd, *J* = 9.2, 2.5 Hz, 1H), 6.11 (d, *J* = 2.4 Hz, 1H), 3.01 (s, 6H).

5-TAMRA

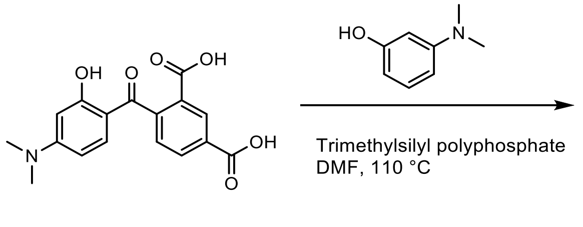

5-TAMRA

To a round-bottom flask, 4-(4-(dimethylamino)-2-hydroxybenzoyl)isophthalic acid (1.94 g, 5.9 mmol, 1.0 eq) and 3-dimethylaminophenol (1.05 g, 7.7 mmol, 1.3 eq) were dissolved in DMF (20 mL). Trimethylsilylpolyphosphate solution in chloroform (6 mL) was added and the mixture was refluxed for 3 h. Solvents were removed *in vacuo* and the residue was dissolved in 5% NaOH (20 mL) and stirred at rt overnight. The mixture was diluted with water (75 mL) and the product was precipitated with concentrated HCl (5 mL). The solid was filtered and washed with cold water (50 mL) and dried at 60°C to give the 5-TAMRA as a purple solid (1.0 g, 39.5% yield). The product was used without further purification. ESI-MS: *m/z* for $C_{25}H_{23}N_2O_5^+$ [M+H] $^+$ calcd 431.2, found 431.0. ^{1}H NMR (700 MHz, DMSO-d_6) δ 8.63 – 8.36 (m, 1H), 8.30 (d, J = 8.1 Hz, 1H), 7.40 (s, 1H), 6.61 (m, 6H), 3.01 (s, 12H).

5-TAMRA-SE

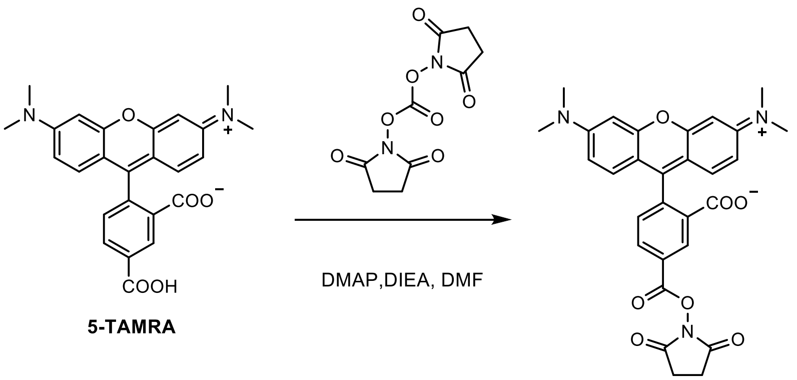

5-TAMRA **5-TAMRA-SE**

To a solution of 5-TAMRA (86 mg, 0.2 mmol, 1.0 eq), DIEA (66 µL, 0.4 mmol, 2.0 eq) and DMAP (49 mg, 0.4 mmol, 2.0 eq) in DMF (2 mL), disuccinimidyl carbonate (51 mg, 0.2 mmol, 1.0 eq) were added. The reaction was stirred for 6 h at rt and analyzed on TLC ($CHCl_3$:MeOH:TEA = 85:10:5, product: R_f = 0.3). Diethyl ether (20 mL) was added to precipitate the product. The solid was filtered, washed with EA, DCM, and a small volume of cold MeOH and dried at 80°C to give the 5-TAMRA-SE as dark purple solid (49 mg, 46.5% yield). 5-TAMRA-SE was used without further purification. ESI-MS: *m/z* for $C_{29}H_{26}N_3O_7^+$ [M+H] $^+$ calcd 528.2, found 528.2.

Solid phase synthesis of KG-TAMRA

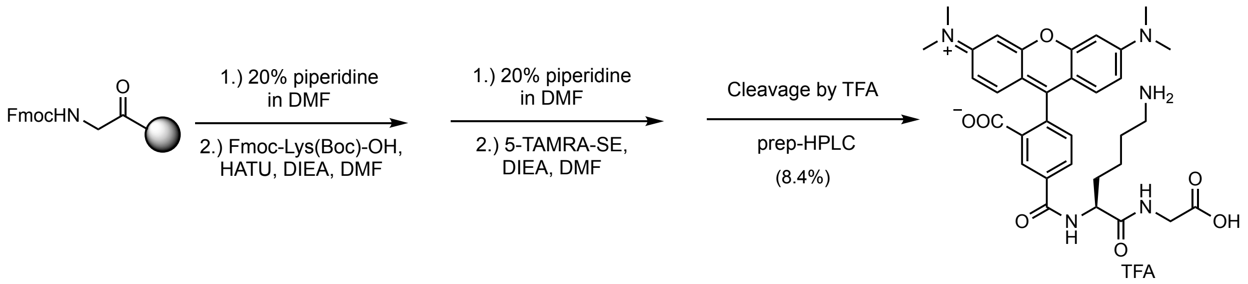

KG-TAMRA

Fmoc-Gly Wang resin (Novabiochem, 100-200 mesh, 36 mg, 0.7 mmol/g) in a fritted syringe was swollen with 5 mL DMF for 20 min. Then, 4 mL 20% piperidine in DMF was added and the resin was agitated for 20 min. The resin was then washed with DMF, MeOH, DCM and DMF (3x 5 mL of each) sequentially. A mixture of Fmoc-Lys(Boc)-OH (60 mg, 0.13 mol, 5.0 eq) and DIEA (30 µL, 0.26 mmol, 10.0 eq) in 3 mL DMF was added into the syringe. HATU (50 mg, 0.13 mmol, 5.0 eq) was added and the resin agitated for 1 h. The resin was then washed as above and Fmoc was cleaved with 20% piperidine in DMF. The resin was then mixed with 5-TAMRA-SE (39.8 mg, 0.08 mol, 3.0 eq) and DIEA (30 µL, 0.26 mmol, 10.0 eq) for 4 h in DMF. The resin was then washed with DMF until the flow-through was colorless. The product was cleaved off the resin with TFA:H_2O:TIPS (95:2.5:2.5) for 30 min. The product was finally purified on a preparative HPLC and lyophilized to give a dark-purple solid (1.5 mg, 8.4% yield). HRMS: *m/z* for $C_{33}H_{38}N_5O_7^+$ $[M+H]^+$ calcd 616.2766, found 616.2762. ^{1}H NMR (700 MHz, D_2O) δ 8.52 (d, *J* = 1.8 Hz, 1H), 8.19 (dd, *J* = 7.8, 1.8 Hz, 1H), 7.64 (d, *J* = 7.8 Hz, 1H), 7.21 (d, *J* = 9.5 Hz, 1H), 7.20 (d, *J* = 9.5 Hz, 1H), 6.88 (dd, *J* = 9.5, 2.3 Hz, 2H), 6.45 (s, 2H), 4.68 (dd, *J* = 8.5, 6.2 Hz, 1H), 4.12 – 4.04 (m, 2H), 3.14 (s, 12H), 3.07 (t, *J* = 6.54 Hz, 2H), 2.09 – 1.97 (m, 2H), 1.84 – 1.76 (m, 2H), 1.68 – 1.60 (m, 2H). ^{13}C NMR (176 MHz, D_2O) δ 174.36, 173.47, 170.58, 169.37, 163.08, 162.88, 157.26, 156.97, 156.92, 135.88, 135.05, 134.78, 130.79, 130.65, 130.28, 128.83, 117.14, 115.48, 113.98, 112.84, 96.05, 56.22, 54.42, 41.37, 39.95, 39.22, 30.51, 26.34, 22.21.

***Tert*-butyl *N*²-(((9*H*-fluoren-9-yl)methoxy)carbonyl)-*N*⁶-(*tert*-butoxycarbonyl)-*L*-lysylglycinate**

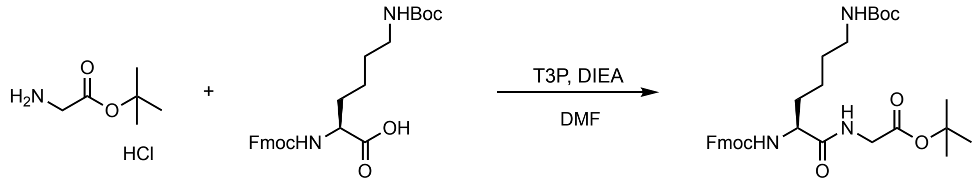

To a 100 mL round-bottom flask, Fmoc-Lys(Boc)-OH (1.406 g, 3.0 mmol, 1.0 eq), glycine *tert*-butyl ester HCl (528 mg, 3.15 mmol, 1.05 eq), T3P (50% in DMF, 2.75 mL, 3.9 mmol, 1.3 eq) and DIEA (1.05 mL, 6.0 mmol, 2.0 eq) were dissolved in anhydrous DMF (30 mL). The reaction was stirred at rt for 3 h and quenched by 20 mL H_2O. The reaction was extracted with EA (100 mL×3). The combined organic phase was washed with brine (100 mL×3), dried over $MgSO_4$, filtered, and concentrated *in vacuo*. The residue was further purified on silica column (DCM:MeOH = 30:1) to give the title compound as a white solid (970 mg, 55.6% yield). ESI-MS: *m/z* for $C_{32}H_{43}N_3O_7Na^+$ $[M+Na]^+$ calcd 604.3, found 604.2. ^1H NMR (600 MHz, $CDCl_3$) δ 7.76 (d, *J* = 7.5 Hz, 2H), 7.60 (d, *J* = 6.8 Hz, 2H), 7.42 – 7.37 (m, 2H), 7.34 – 7.29 (m, 2H), 6.47 (s, 1H), 5.47 (s, 1H), 4.64 (s, 1H), 4.48 – 4.34 (m, 2H), 4.27 – 4.14 (m, 2H), 4.00 – 3.85 (m, 2H), 3.19 – 3.01 (m, 2H), 1.96 – 1.48 (m, 4H), 1.46 (s, 9H), 1.43 (s, 9H), 1.41 – 1.23 (m, 2H). ^{13}C NMR (151 MHz, $CDCl_3$) δ 171.77, 168.77, 156.32, 143.96, 143.92, 141.45, 127.86, 127.24, 125.25, 120.12, 82.58, 79.33, 67.20, 54.88, 47.31, 42.15, 40.02, 32.23, 29.77, 28.58, 28.19, 22.54.

***Tert*-butyl *N*⁶-(*tert*-butoxycarbonyl)-*L*-lysylglycinate**

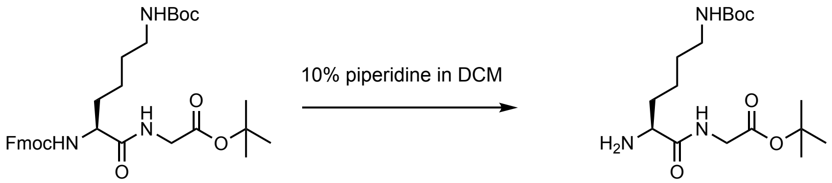

To a 50 mL round-bottom flask, *tert*-butyl *N*²-(((9*H*-fluoren-9-yl)methoxy)carbonyl)-*N*⁶-(*tert*-butoxycarbonyl)-*L*-lysylglycinate (734 mg, 1.26 mmol, 1.0 eq) was mixed with 10% piperidine in DCM (8 mL). The reaction was stirred for 1 h. 10 mL PE:diethyl ether (1:1) was added. The resulting mixture was filtered, and the organic phase was concentrated and purified via silica column (DCM:MeOH = 20:1) to give the title compound as a white sticky solid (296 mg, 65.4% yield). ESI-MS: *m/z* for $C_{17}H_{34}N_3O_5^+$ $[M+H]^+$ calcd 360.2, found 360.0. ^1H NMR (600 MHz, $CDCl_3$) δ 7.72 (s, 1H), 4.59 (s, 1H), 3.98 – 3.88 (m, 2H), 3.42 (dd, *J* = 7.9, 4.5 Hz, 1H), 3.16 – 3.04 (m, 2H), 1.89 – 1.82 (m, 1H), 1.77 (s, 2H), 1.60 – 1.53 (m, 1H), 1.52 – 1.48 (m, 2H), 1.47 (s, 9H), 1.43 (s, 9H), 1.42 – 1.34 (m, 2H). ^{13}C NMR (151 MHz, $CDCl_3$) δ 175.17, 169.32, 156.21, 82.27, 79.23, 55.08, 41.79, 40.31, 34.62, 30.02, 28.57, 28.21, 22.96.

(*S*)-2-(6-(dimethylamino)-3-(dimethyliminio)-3*H*-xanthen-9-yl)-5-((2,2,16,16-tetramethyl-4,11,14-trioxo-3,15-dioxa-5,12-diazaheptadecan-10-yl)carbamoyl)benzoate

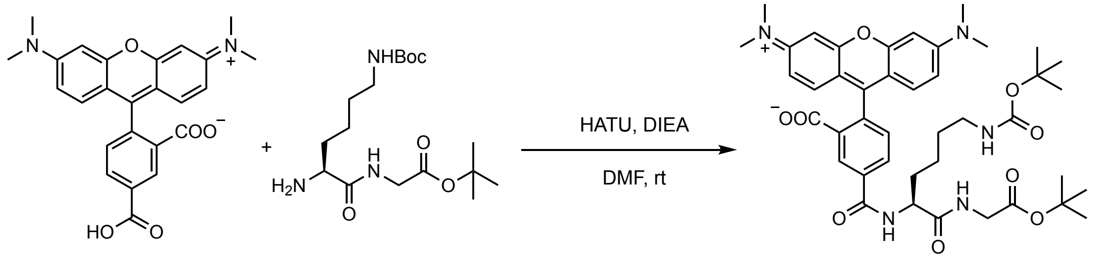

5-TAMRA (50 mg, 0.12 mmol, 1.0 eq) was dissolved in 4 mL anhydrous DMF, then *tert*-butyl N^6-(*tert*-butoxycarbonyl)-*L*-lysylglycinate (50.1 mg, 0.14 mmol, 1.2 eq) and DIPEA (60.7 µL, 0.35 mmol, 3.0 eq) were added. After dissolving all components, HATU (48.6 mg, 0.13 mmol, 1.1 eq) was added and the mixture was stirred for 17 hours at rt. After full conversion of 5-TAMRA, the solvent was removed under reduced pressure and the product was purified by preparative medium pressure liquid chromatography eluting at 40-45 % solvent B to give the crude product which was directly used for the next step. ESI-MS: *m/z* for $C_{42}H_{54}N_5O_9^+$ $[M+H]^+$ calcd 772.4, found 772.4.

KG-TAMRA

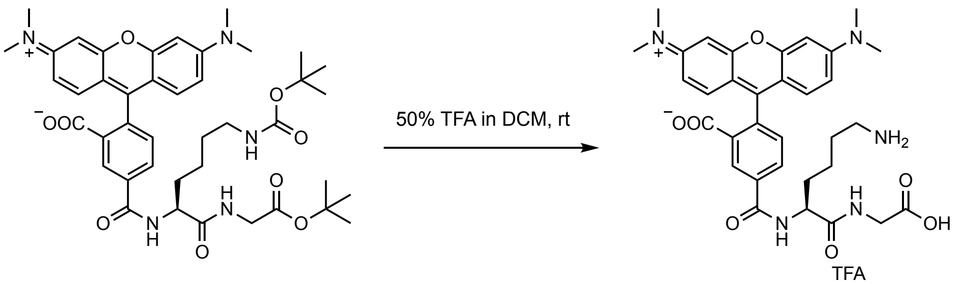

(*S*)-2-(6-(dimethylamino)-3-(dimethyliminio)-3*H*-xanthen-9-yl)-5-((2,2,16,16-tetramethyl-4,11,14-trioxo-3,15-dioxa-5,12-diazaheptadecan-10-yl)carbamoyl)benzoate was dissolved in 1.5 mL DCM followed by addition of 1.5 mL TFA. The reaction mixture was stirred at rt until complete deprotection. After 90 minutes the reaction was diluted with DCM and the solvent was removed under reduced pressure. KG-TAMRA was obtained as a purple powder (64.9 mg, 76.0% yield over two steps) after purification by preparative HPLC and lyophilization with virtually identical characteristics as specified above. ESI-MS: *m/z* for $C_{33}H_{38}N_5O_7^+$ $[M+H]^+$ calcd 616.3, found 616.0. ^1H NMR (700 MHz, D_2O) δ 8.40 (d, *J* = 1.8 Hz, 1H), 8.13 (d, *J* = 7.9 Hz, 1H), 7.67 – 7.58 (m, 1H), 7.23 (d, J = 9.4 Hz, 1H), 7.21 (d, *J* = 9.4 Hz, 1H), 6.90 (dd, *J* = 9.5, 1.9 Hz, 2H), 6.59 (s, 2H), 4.67 (dd, *J* = 8.6, 6.1 Hz, 1H), 4.07 – 3.98 (m, 2H), 3.17 (s, 12H), 3.07 (t, *J* = 7.5 Hz, 2H), 2.11 – 1.96 (m, 2H), 1.84 – 1.74 (m, 2H), 1.69 – 1.57 (m, 2H).

Cloning, Protein Expression and Purification

Human USPL1 catalytic domain constructs (residues 212-514 and 218-502) were cloned from a cDNA template (coding sequence corresponding to UniProt entry Q5W0Q7) into the pOPIN-K vector using the In-Fusion HD Cloning Kit (Takara Clonetech). Human SENP1 (residues 415-644, UniProt: Q9P0U3) and USP21 (residues 196-565, UniProt: Q9UK80) were subcloned into the pOPIN-B vector. Human UCHL3 (residues 1-230 in pOPIN-K, UniProt: P15374)[10], USP36 (residues 81-461 in pOPIN-K, UniProt: Q9P275)[11], USP16 (residues 191-823 in pOPIN-K, UniProt: Q9Y5T5)[11], USP2 (residues 258-605 in pOPIN-B, UniProt: O75604)[11], USP18 (residues 16-372 in pOPIN-B, UniProt: Q9UMW8)[11], and USP7 (residues 1-1102 in pFL, UniProt: Q93009)[12] were expressed and purified as described previously. Site-directed mutagenesis was performed using a QuikChange protocol with Phusion Polymerase (New England BioLabs).

For bacterial expression of USPL1, *Escherichia coli* Rosetta 2(DE3)pLacI cells (Merck) were chemically transformed and grown in 2xTY medium with antibiotics at 37°C. When the OD_{600} reached ~0.8, the culture was cooled down to 18°C for 1 h and induced with 0.5 mM IPTG. Cells were harvested after 12-16 h growth at 18°C by centrifugation at 4000 x *g* for 10 min and purified immediately or stored at -80°C. For purification of USPL1, cell pellets were resuspended in binding buffer (50 mM sodium orthophosphate, 300 mM NaCl, 20 mM imidazole, pH 8.0, 4 mM β-mercaptoethanol, supplemented with DNase I and lysozyme) and lysed using ultrasonication. Cell debris was discarded by centrifugation and the lysate was filtered through a 0.45 µm filter. The clarified lysate was applied to a HisTrap Ni-NTA column (GE Healthcare) and then eluted by elution buffer (50 mM sodium orthophosphate, 300 mM NaCl, 500 mM imidazole, pH 8.0, 4 mM β-mercaptoethanol). Protein-containing fractions were pooled, supplemented with His-3C protease, and dialyzed into binding buffer at 7°C overnight. The sample was then passed through a Ni-NTA column. The flow-through was collected and dialyzed into anion exchange low salt buffer (25 mM Tris, 50 mM NaCl, 4 mM DTT, pH 8.5) at 7°C overnight. The sample was applied to a Resource Q column (GE Healthcare) and purified with gradient elution from 50 to 500 mM NaCl. Pure fractions were collected, concentrated to ~2-3 mL and further purified by size-exclusion chromatography (HiLoad 16/600 Superdex 75pg, GE Healthcare) with SEC buffer (20 mM Tris, 100 mM NaCl, 4 mM DTT, pH 8.0). Fractions were analyzed on SDS-PAGE protein gels (NuPAGE 4-12%, Invitrogen) which were stained by InstantBlue (Abcam). Final protein samples were concentrated using 10K MWCO Amicon Ultra Filters (Merck Millipore), flash frozen in liquid nitrogen and stored at -80°C.

SENP1 was purified using a similar protocol as for USPL1 with the exception that binding and elution buffers were adjusted to pH 7.6. The pooled fractions were digested by His-3C protease and dialyzed into binding buffer (pH 7.6). The sample was passed through a Ni-NTA column and the flow-through was collected, concentrated, and subjected to size-exclusion chromatography (HiLoad 16/600 Superdex 75pg, GE Healthcare) with buffer (20 mM Tris, 100 mM NaCl, 4 mM DTT, pH 7.6). The final protein was concentrated, flash frozen in liquid nitrogen and stored at -80°C. USP21 was purified using the same protocol for USPL1 except for the SEC buffer which was supplemented with 5% glycerol.

Yeast ULP1 (residues 403-621 in pET19, UniProt: Q02724) was a kind gift from the Protein Chemistry Facility (Max Planck Institute of Molecular Physiology Dortmund).

For purification of human SUMO1 (1-97, UniProt: P63165), SUMO2 (1-93, UniProt: P61956), SUMO3 (1-92, UniProt: P55854), and SUMO3 pro-form (1-103), genes were subcloned into the pOPIN-B vector and expressed in Rosetta 2(DE3)pLacI cells as above. The proteins were purified on a Ni-NTA column as above. After His-3C cleavage, the proteins were passed through a Ni-NTA column again. The flow-through was collected, analyzed on a gel, and used without further purification.

To generate C-terminal thioesters (Ub/Ubl-MesNa), human Ub (1-75), Ub (1-76), SUMO1 (1-96), SUMO1 (1-97), SUMO2 (1-92), SUMO2 (1-93), ΔN-SUMO2 (18-92), SUMO3 (1-91), NEDD8 (1-76, UniProt: Q15843), C-terminal Ubl domain of ISG15 (ISG15(CTD), 77-157, C78S, UniProt: P05161) and Fubi (1-74, C57A, UniProt: P62861) were subcloned into the pTXB1 vector and expressed in Rosetta 2(DE3)pLacI cells as described as above. Cells were resuspended in buffer A (20 mM HEPES, 50 mM sodium acetate, 75 mM NaCl, pH 6.5) supplemented with DNase I and lysed using ultrasonication (all buffers were supplemented with 5% glycerol for Fubi purifications). The suspension was cleared by centrifugation and filtered through a 0.45 μm filter. The clarified lysate was loaded onto pre-equilibrated chitin resin (NEB S6651L) and incubated with over-head rotation at 7°C overnight. The resin was washed with 1 L high salt buffer (20 mM HEPES, 50 mM sodium acetate, 500 mM NaCl, pH 6.5) and then washed with 100 mL buffer A. Elution was initiated by addition of 50 mL sodium 2-mercaptoethanesulfonate solution (MesNa, 100 mM in buffer A). After incubation at 7°C for 24-36 h the eluate was collected and concentrated to less than 5 mL. Size-exclusion chromatography (HiLoad 26/600 Superdex 75 pg, GE Healthcare) was used to purify the protein into buffer A. The fractions were analyzed on a gel, concentrated, and stored at –80°C. Protein mass spectrometry revealed full cleavage of the N-terminal methionine for both SUMO2 (1-92) and SUMO3 (1-91) proteins. Fubi-MesNa was collected after eluted from chitin column, concentrated, flash frozen in liquid nitrogen and stored at -80°C for further use.

Selenomethionine (SeMet)-containing protein expression was conducted by the methionine pathway inhibition method in *E. coli* Rosetta 2(DE3) pLacI cells.[13] The pTXB1 plasmid encoding SUMO3 (1-91) was freshly transformed into cells, and a culture in 2xTY medium with antibiotics was inoculated in the morning. After 10 h, a pre-culture in M9 minimal medium was started with a 1:1000 inoculum. On the second day, a large culture in M9 minimal medium was started with a 1:100 inoculum. When the OD_{600} reached 0.6, feed-back inhibition amino acids including 1.0 g each of lysine, threonine and phenylalanine, 0.5 g each of leucine, isoleucine and valine, and 0.5 g of selenomethionine for 10 L of culture were added. After shaking for 15 min, the culture was cooled down to 18°C for 1 h and then induced by 0.5 mM IPTG for 16 h. Protein purification was performed as above. Complete selenomethionine incorporation was validated by protein LC-MS analysis (mass calculated for SeMet-SUMO3-MesNa: 10600.4 Da, mass found: 10601.2 Da).

RanGAP1 (399-587, UniProt: P46060) was cloned from a synthesized gene into pOPIN-B and expressed in Rosetta 2(DE3)pLacI cells. Purification was carried as described above by a Ni-NTA column, cleavage by His-3C protease, reverse Ni-NTA purification and anion exchange chromatography, similar to what was previously

reported[14]. Full length human SAE1/SAE2 (from a pETDuet1 plasmid encoding GST-SAE1 and His6-UBA2), and UBE2I (from a pGEX-6P1 plasmid) were expressed in Rosetta 2(DE3) pLacI cells. Purification of SAE1/SAE2[15] and UBE2I[14] were carried out similarly to what was described previously.

Protein concentrations were determined by absorption at 280 nm unless noted otherwise. Intact protein mass spectrometry was recorded on an Agilent 1260 II Infinity system (Agilent, with Openlab software) and analyzed as reported previously.[10]

Preparation of SUMO3-2Br and ΔN-SUMO2-PA Probes

To a solution of SUMO3(1-91)-MesNa (958.7 µM, 3.5 mL), 2-bromoethylamine hydrobromide (1.6 g in ~5 mL of buffer A) was added and the pH was adjusted by addition of 4 M NaOH to 8.0-8.5 at rt. The reaction was monitored by LC-MS. After 1-2 h, SUMO3(1-91)-MesNa was fully consumed, and the mixture was subjected to size-exclusion chromatography (HiLoad 26/600 Superdex 75 pg, GE Healthcare) in buffer A. The protein-containing fractions were pooled, concentrated, and stored at -80°C by flash freezing for further use. ΔN-SUMO2-PA was generated using a similar procedure by mixing ΔN-SUMO2-MesNa (767.5 µM, 1.5 mL) and propargylamine hydrocholoride (600 mg in ~3 mL of buffer A).

Crystallography

Covalent complexes of USPL1 (218-502) with SUMO3 probes were prepared by mixing USPL1 (1.0 eq, 0.19 mg/mL, after reverse Ni-NTA purification step in Ni-NTA binding buffer) and SUMO3 probes (4.0 eq, 531 µM, fully purified) at rt for 4 h. The reaction mixture was subjected to purification on a Resource Q column as described above. Pure fractions containing the covalent complex were then concentrated, and buffer exchanged into SEC buffer (20 mM Tris, 100 mM NaCl, 4 mM DTT, pH 8.0). Since USPL1 could not be produced with SeMet labeling in high enough yields, crystals for the SAD dataset were prepared from unlabeled USPL1 and SeMet-containing SUMO3-2Br probe (3 Se sites per 378 residues).

Crystallization experiments were carried out at 18°C in 96-well sitting drop vapor diffusion plates (MRC format, Molecular Dimensions). Plates were set up using a Mosquito HTS robot (TTP Labtech) and fine screen plates were prepared with a Dragonfly robot (TTP Labtech). Coarse screening was carried out by mixing 200 nL of protein solution with 200 nL of reservoir. Fine screens were assembled in 600-900 µL drops with ratios as described below. USPL1~SUMO3-2Br (7.0 mg/mL) was crystallized in 100 mM Tris pH 8.8, 19% PEG 6000, 200 mM $CaCl_2$, from mixing protein : reservoir in a 1:2 ratio. USPL1~SeMet-SUMO3-2Br (5.4 mg/mL) was crystallized in 100 mM Tris pH 8.3, 21% PEG 4000, 200 mM $CaCl_2$, from mixing protein : reservoir in a 2:1 ratio. Crystals were collected with the protection of mother liquor containing 25% glycerol and vitrified in liquid nitrogen. USPL1~ΔN-SUMO2-PA (8.6 mg/mL) was crystallized in 100 mM CHES pH 8.9, 34% PEG 600, from mixing protein : reservoir in a 2:1 ratio. Crystals were cryo-protected in 100 mM CHES pH 9.2, 45% PEG 600, and vitrified in liquid nitrogen.

Diffraction data were collected at 100 K at the Swiss light source (SLS, Paul Scherrer Institute, Villingen, Switzerland) on beamlines PX2 and PX3. Images were integrated using either XDS[16] (for SeMet data from four 360° sweeps with 5° increments using the PriGo goniometer) or DIALS[17] (for native datasets), and scaled using Aimless[18]. Owing to the low sequence homology between the USPL1 catalytic domain and other previously crystallized members of the USP family (highest identity: USP7 at 16%), various molecular replacement efforts from the native datasets were not successful. The structure of USPL1~SUMO3-2Br was solved experimentally through the CRANK2[19] pipeline by a SAD protocol as implemented in the CCP4 suite of programs from the SeMet dataset involving substructure detection by SHELXD[20], density modification by Parrot[21] and model building/refinement by Buccaneer/Refmac[22]. The resulting initial model was then used to obtain phases for the native dataset, and the final model was obtained through several rounds of manual building in Coot[23] and refinement by Phenix.Refine[24]. The USPL1~ΔN-SUMO2-PA structure was solved through molecular replacement with Phaser[25] with one copy of the USPL1-SUMO3-2Br complex as search model, and refined with external restraints for USPL1 against the final coordinates of the USPL1-SUMO3-2Br structure due to the high Wilson *B* factor. See Table S1 for final statistics. Protein structures have been deposited with the protein data bank under ID codes 7ZJU and 7ZJV. The one letter amino acid code was used for mutations and alignments, whereas the three letter code was used to point out individual amino acids for improved readability. Protein structure figures were generated with PyMol.

Preparation and Purification of RanGAP1-SUMO2

RanGAP1~SUMO2 wildtype (wt) and mutant conjugates were assembled by the catalysis of SAE1/SAE2 and UBE2I as described previously[26]. In general, 5 mL reactions in transport buffer (20 mM HEPES, 110 mM NaOAc, 2 mM $Mg(OAc)_2$, 1 mM DTT, pH 7.3 using 1 N KOH) containing 3.4 µM RanGAP1, 50 nM SAE1/SAE2, 30 nM UBE2I and 12 µM SUMO2 wt or mutants were initiated by addition of 2.5 mM ATP and incubated at 37°C for 2-3 h. The mixture was subjected to size-exclusion chromatography (HiLoad 26/600 Superdex 75pg, GE Healthcare) with elution into transport buffer. The proteins were flash frozen and store at -80°C for further use.

Gel-based Cleavage Assays

USPL1 (5 nM) and RanGAP1-SUMO2 (5 µM) were mixed at different time points and incubated at 37°C in assay buffer (20 mM HEPES, pH 7.5, 100 mM NaCl, 5 mM DTT, 0.01 mg/mL BSA). The reaction was quenched by adding LDS sample loading buffer and analyzed on NuPAGE gels with Coomassie staining.

USPL1 wt and mutants (500 nM) were mixed with full-length SUMO3 (10 µM) and incubated at 37°C for 2 h in assay buffer (20 mM HEPES, pH 7.5, 100 mM NaCl, 5 mM DTT, 0.01 mg/mL BSA). The reaction was quenched by adding LDS sample loading buffer and analyzed on NuPAGE gel with Coomassie staining.

Thermal Shift Assay

Assays were performed in triplicate in PBS containing 4 mM DTT. 5 µL USPL1 (10 µM) and 25 µL SUMO3 (19.2 µM) probe were mixed and incubated at room temperature for 30 min, then 10x Sypro-Orange (10 µL) was added, and the melting curves were measured and analyzed on a BioRad CFX-Connect qPCR instrument.

Fluorescence Intensity Kinetic assays

Enzymes and substrates were diluted into assay buffer (20 mM HEPES, pH 7.5, 100 mM NaCl, 5 mM DTT, 0.01 mg/mL BSA). Substrates were used at 50 nM of final concentration unless noted otherwise. Assays were carried out in black 384 well low volume non-binding plates (Greiner 784900) with 20 µL total volume. After addition of enzyme, fluorescence (ex/em = 485/535 nm) was recorded on a Tecan Spark plate reader at ambient temperature.

Preparation and Purification of SUMO and Ub Rhodamine Substrates

Reaction conditions were optimized from a previously reported method for Ubiquitin-RhoG[3]. To a solution of SUMO2(1-92)-MesNa (511 µM, ~10 mL in buffer A) were added 10 eq. *N*-hydroxysuccinimide (1 M in buffer A, pH adjusted to 7.5), 10 eq. *sym*-collidine and 5 eq. 2G-Rho (50 mM in DMSO, the final DMSO concentration is below 5%, see above for the synthesis of 2G-Rho), the pH which was measured by a pH meter (Mettler Toledo) was adjusted to 8.0 using 4 N NaOH and then the reaction mixture was heated to 37°C. The reaction was monitored by LC-MS until most of the starting material was converted to product or hydrolyzed. The reaction was then diluted and dialyzed against low salt cation exchange buffer (20 mM MES, pH 6.0, 50 mM NaCl, 1 mM TCEP) overnight. The sample was filtered, the filtrate applied to a Resource S column (GE Healthcare), and the product eluted with a gradient of 50-1000 mM NaCl. The fractions were analyzed on a gel and evaluated by intact protein LC-MS. Pure fractions were pooled and dialyzed into buffer (20 mM MES, pH 6.0, 100 mM NaCl). The final product was filtered and concentrated to ~1 mg/mL. SUMO1-RhoG was prepared and purified using the same method. Ub-RhoG was purified using the buffer with the same components, but the pH was adjusted to 6.5. The concentration was measured based on a calibration curve made from RhoG. The final products were validated by LC-MS. SUMO1-RhoG, calcd 11370.7, found 11369.8; SUMO2-RhoG, calcd 10847.1, found 10847.9; Ub-RhoG, calcd 8934.2, found 8938.1 (Figure S2B).

Preparation of Ub/Ubl-$NHNH_2$

Ub/Ubl-MesNa were treated with hydrazine monohydrate (64% in H_2O, 500 µL added into 100 mL eluate). After 30 min at room temperature, the reaction was concentrated, filtered, and purified by size-exclusion chromatography (HiLoad 16/600 Superdex 75 pg) using buffer (20 mM MES, 50 mM NaCl, pH 6.7). Ub/Ubl-$NHNH_2$ proteins were validated by LC-MS: Ub-$NHNH_2$ calcd 8578.9, found 8582.1; SUMO1-$NHNH_2$ calcd 11015.4, found 11014.6; SUMO2-$NHNH_2$ calcd 10491.8, found 10490.3; NEDD8-$NHNH_2$ calcd 8574.0, found 8573.4;

ISG15(CTD)-$NHNH_2$ calcd 9261.6, found 9261.1; Fubi-$NHNH_2$ cacld 7741.8, found 7741.1 (Figures S3A, S7C and S7D).

Preparation and purification of Ub-KG-TAMRA

To a solution of $Ub^{1-76(wt)}$-$NHNH_2$ (2 mM, 100 µL) at -10°C were added sodium nitrite (1 M, 50 µL) and citric acid (200 mM, 50 µL). After 2 min, KG-TAMRA (10 mM in 1.5 M HEPES pH 8.0, 200 µL) was added and the reaction was incubated at 30°C for 3 min. The reaction was analyzed by LC-MS, diluted to 40 mL, and dialyzed into buffer (20 mM MES, pH 6.0) overnight. The mixture was purified by cation exchange chromatography (Resource S) in the same buffer with gradient elution from 0 to 300 mM NaCl. Protein-containing fractions were pooled and dialyzed into low pH buffer (20 mM NaOAc, pH 4.5). These were further purified by cation exchange chromatography (Resource S) in low pH buffer with gradient elution from 0 to 300 mM NaCl (Figure S1B). Ub-KG-TAMRA was concentrated, flash frozen in liquid nitrogen, stored at -80°C and validated by LC-MS (Figure 1J).

Preparation and purification of SUMO1-KG-TAMRA

To a solution of $SUMO1^{1-97(wt)}$-$NHNH_2$ (1.8 mM, 100 µL) at -10°C were added sodium nitrite (1 M, 50 µL) and citric acid (200 mM, 50 µL). After 2 min, KG-TAMRA (20 mM in 1.5 M HEPES, pH 8.0, 200 µL) was added and the reaction was incubated at 30°C for 3 min. The reaction was analyzed by LC-MS. Nitrosated species were fully reversed by addition of TCEP solution (1 M in 20 mM MES, pH 7.0, 1 mL). The reaction mixture was then passed through a 0.45 µm filter and purified by size-exclusion chromatography (HiLoad 16/600 Superdex 75 pg) with isocratic elution in buffer (20 mM Tris, 50 mM NaCl, 1 mM DTT, pH 8.0). Protein-containing fractions were pooled, diluted with 20 mM Tris buffer (pH 7.0) and purified on a high-resolution anion exchange column (Capto HiRes Q 5/50, Cytiva) with gradient elution from 0 to 500 mM NaCl (Figure S1B). SUMO1-KG-TAMRA-containing fractions were identified by LC-MS (Figure 1J), concentrated, flash frozen in liquid nitrogen, stored at -80°C.

Preparation and purification of SUMO2-KG-TAMRA

To a solution of $SUMO2^{1-93(wt)}$-$NHNH_2$ (7.6 mM, 80 µL) at -10°C were added sodium nitrite (1 M, 40 µL) and citric acid (200 mM, 40 µL). After 2 min, KG-TAMRA (20 mM in 1.5 M HEPES, pH 8.0, 150 µL) was added and the reaction was incubated at 30°C for 3 min. The reaction was analyzed by LC-MS, diluted, and dialyzed into buffer (20 mM MES, pH 6.0) overnight. The mixture was purified by cation exchange chromatography (Resource S) with gradient elution from 0 to 300 mM NaCl. Protein-containing fractions were pooled, dialyzed into buffer (20 mM HEPES, pH 8.0) and purified by size-exclusion chromatography (HiLoad 16/600 Superdex 75 pg) in the same buffer (Figure S1B). SUMO2-KG-TAMRA was concentrated, flash frozen in liquid nitrogen, stored at -80°C and validated by LC-MS (Figure 1J). Remaining traces of dimeric SUMO2-KG-TAMRA species could be completely separated by an additional round of size-exclusion chromatography in 20 mM HEPES, pH 8.0.

Preparation and purification of NEDD8-KG-TAMRA

To a solution of NEDD8$^{1-76(wt)}$-$NHNH_2$ (1.77 mM, 100 µL) at -10°C were added sodium nitrite (1 M, 50 µL) and citric acid (200 mM, 50 µL). After 3 min, KG-TAMRA (5 mM in 1.5 M HEPES, pH 8.0, 200 µL) was added and the reaction was incubated at 30°C for 5 min. The reaction mixture was analyzed by LC-MS, passed through a 0.45 µm filter and purified by size-exclusion chromatography (HiLoad 16/600 Superdex 75 pg) with isocratic elution in buffer (20 mM NaOAc, 50 mM NaCl, pH 5.0). Protein-containing fractions were pooled and further purified via cation exchange chromatography (Resource S) with gradient elution from 50 to 500 mM NaCl (Figure S7A). Fractions were analyzed using LC-MS. The product NEDD8-KG-TAMRA was concentrated, flash frozen in liquid nitrogen, stored at -80°C and validated by LC-MS (Figure 5A).

Preparation and purification of ISG15(CTD)-KG-TAMRA

To a solution of ISG15(CTD)$^{Met+77-157(C78S)}$-$NHNH_2$ (1.77 mM, 150 µL) at -10°C were added sodium nitrite (1 M, 75 µL) and citric acid (200 mM, 75 µL). After 3 min, KG-TAMRA (10 mM in 1.5 M HEPES, pH 8.0, 200 µL) was added and the reaction was incubated at 30°C for 3 min. The reaction mixture was then passed through a 0.45 µm filter and purified by size-exclusion chromatography (HiLoad 16/600 Superdex 75 pg) with isocratic elution in buffer (20 mM Tris, 50 mM NaCl, pH 7.0). Protein-containing fractions were pooled and dialyzed into a high salt buffer (50 mM NaH_2PO_4, 1.5 M $(NH_4)_2SO_4$, pH 7.0) overnight. The sample was further purified using hydrophobic interaction chromatography (HiTrap Capto Phenyl ImpRes, Cytiva) with gradient elution from 1.5 M to 0 M $(NH_4)_2SO_4$ (Figure S7A). Fractions were analyzed using LC-MS. The product ISG15(CTD)-KG-TAMRA was concentrated and buffer-exchanged into low salt buffer (50 mM NaH_2PO_4, pH 7.0), flash frozen in liquid nitrogen, stored at -80°C and validated by LC-MS (Figure 5A).

Preparation and purification of Fubi-KG-TAMRA

To a solution of Fubi$^{1-74(C57A)}$-$NHNH_2$ (100 µL) at -10°C was added sodium nitrite (1 M, 50 µL) and the mixture was incubated for 2 min. Citric acid (200 mM, 50 µL) was then added. After 3 min, KG-TAMRA (20 mM in 1.5 M HEPES, pH 8.0, 200 µL) was added and the reaction was incubated at 30°C for 30 min. The reaction was diluted, and the buffer was exchanged to 50 mM NaCl, 20 mM MES, 5% glycerol, pH 6.5 using size-exclusion chromatography (HiLoad 16/600 Superdex 75 pg). Protein-containing fractions were further purified by high-resolution anion exchange chromatography (Capto HiRes Q 5/50, Cytiva) with gradient elution from 50 to 500 mM NaCl (Figure S7B). Purity of the fractions was assessed by LC-MS. Fubi-KG-TAMRA was concentrated, flash frozen in liquid nitrogen, stored at -80°C and validated by LC-MS (Figure 6B).

Fluorescence polarization assay

Enzymes and substrates were diluted into assay buffer (20 mM HEPES, pH 7.5, 100 mM NaCl, 5 mM DTT, 0.01 mg/mL BSA). Assays were performed in black 384 well low volume non-binding plates (Greiner 784900) with 20 µL total volume. For USP36, USP16, USP7 and USP2 (single concentration with the five different substrates, and all USP16 and USP36 experiments), assays were performed in buffer (20 mM Tris, pH 8.0, 0.01% Triton X-100, 0.1 mg/mL BSA, 1 mM TCEP) in 384-well low volume black round bottom plates (Corning 4514) with 20 µL total volume. For cleavage assays, substrates were used at 100 nM of final concentration unless noted otherwise. For binding assay, KG-TAMRA and Ub-KG-TAMRA were used at 3 nM of final concentration and SUMO1-KG-TAMRA and SUMO2-KG-TAMRA were used at 1 nM of final concentration unless noted otherwise. After addition of enzyme, fluorescence polarization (ex/em = 535/590 nm) was recorded on a Tecan Spark plate reader at ambient temperature.

Circular dichroism spectroscopy

Protein samples were buffer exchanged into 10 mM sodium phosphate pH 7.0 and diluted to 0.1 – 0.5 mg/mL. CD spectra were measured on a Jasco J-815 spectrophotometer at 20 °C with 100 nm/min scan speed. Data were recorded from 190 to 300 nm with a 1 nm pitch and as averages of three technical replicates. Protein concentrations of reference and sample proteins were adjusted by Coomassie-stained SDS PAGE gels and densitometry.

Quantification and Statistical Analysis

Statistical details of the experiments can be found in the figure legends and in the figures. Data are given as mean ± standard deviation (s.d.) or mean ± standard error (s.e.m.) as defined in the legend.

4. NMR spectra

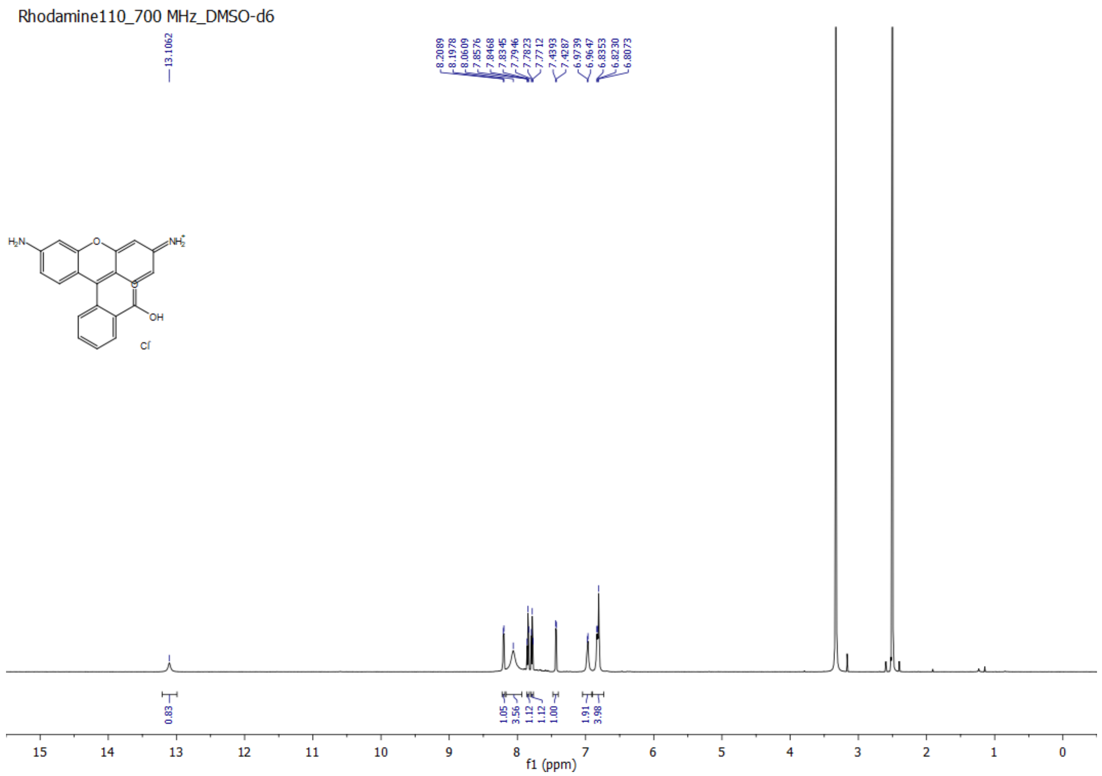

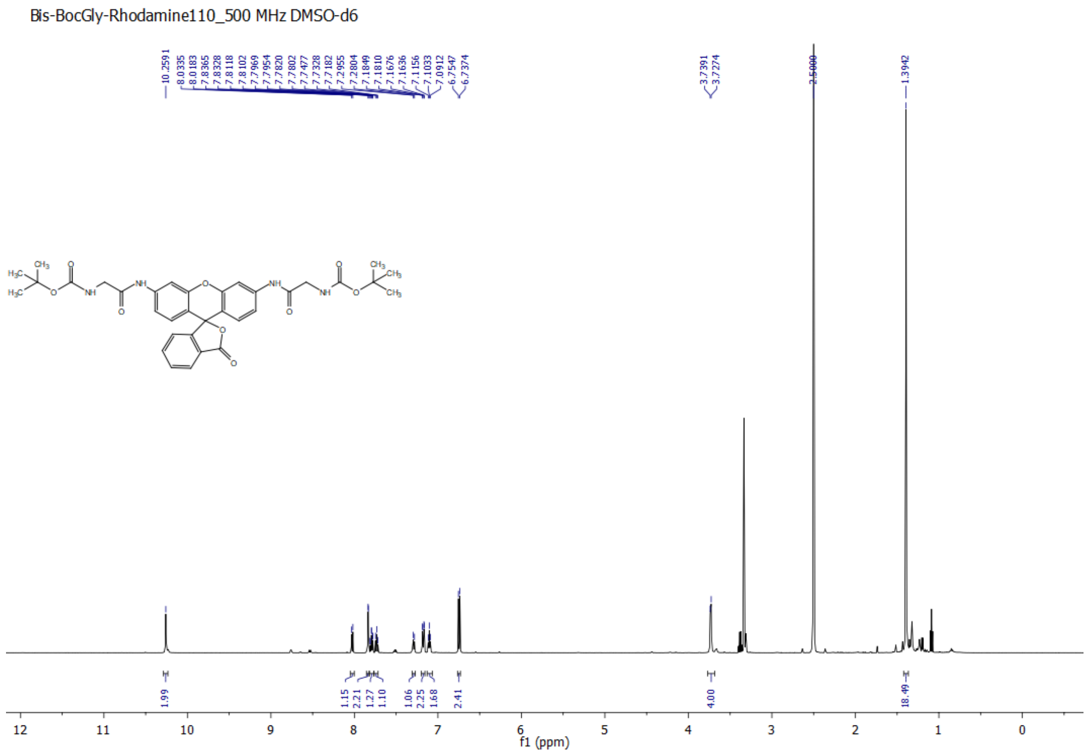

Bis-Gly-Rhodamine110 (2G-Rho)_500 MHz D2O

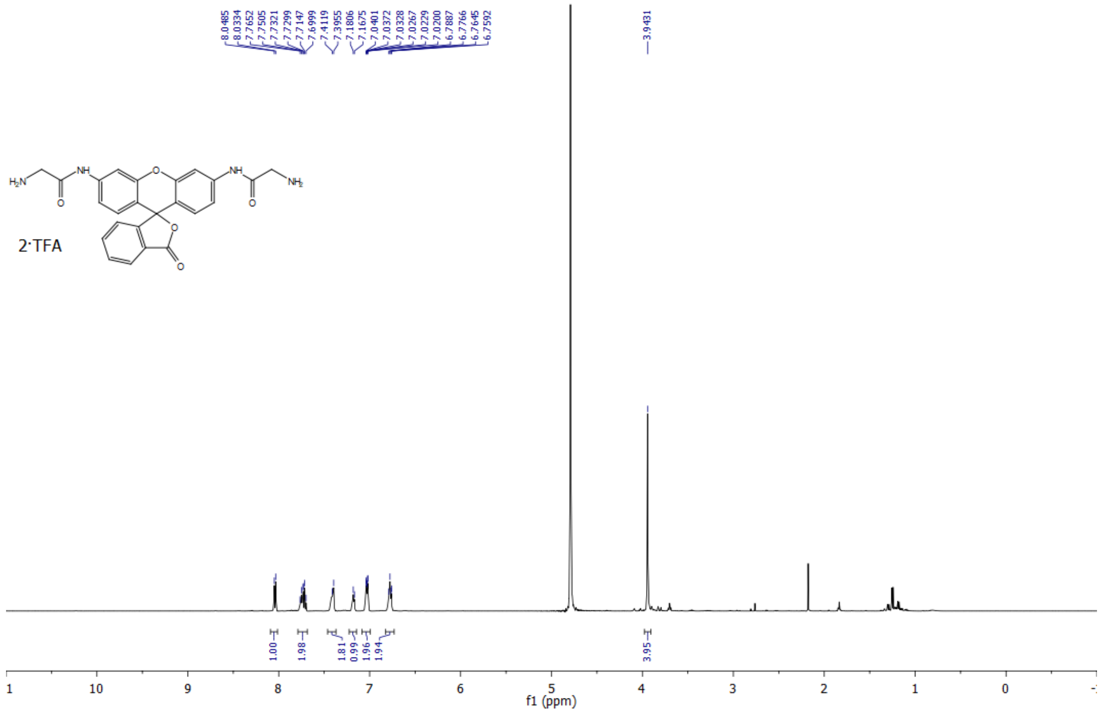

mono Boc-protected Rhodamine110_700 MHz DMSO-d6

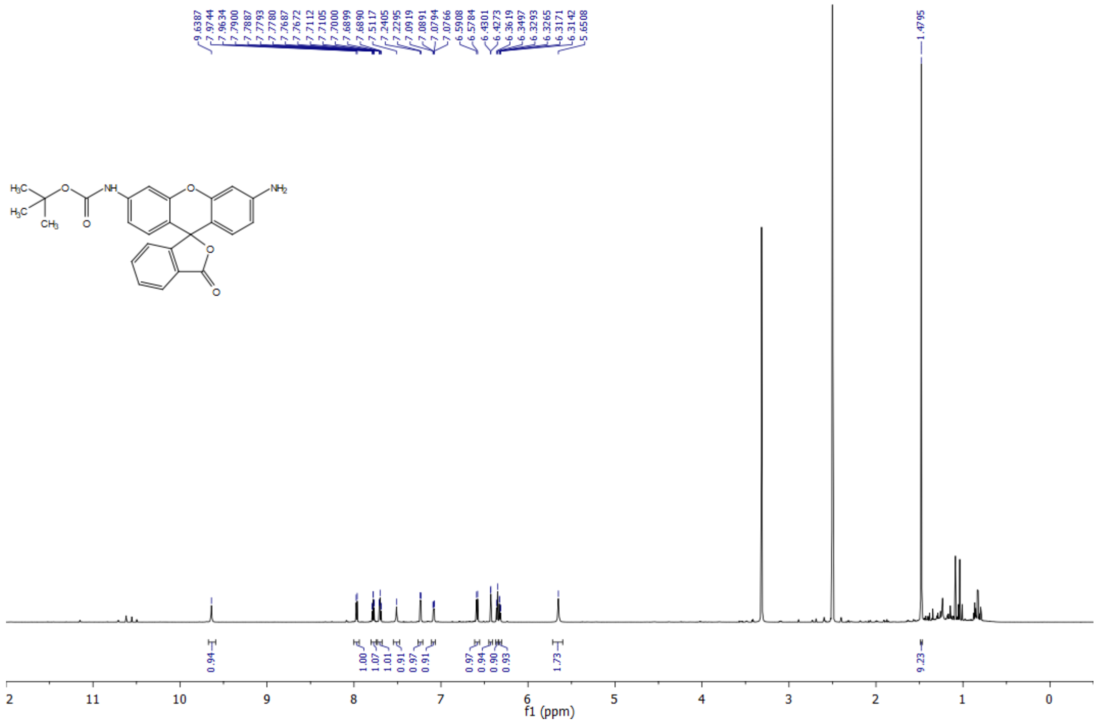

Dual-Boc-protected Gly-Rhodamine110_700 MHz 1H CDCl3

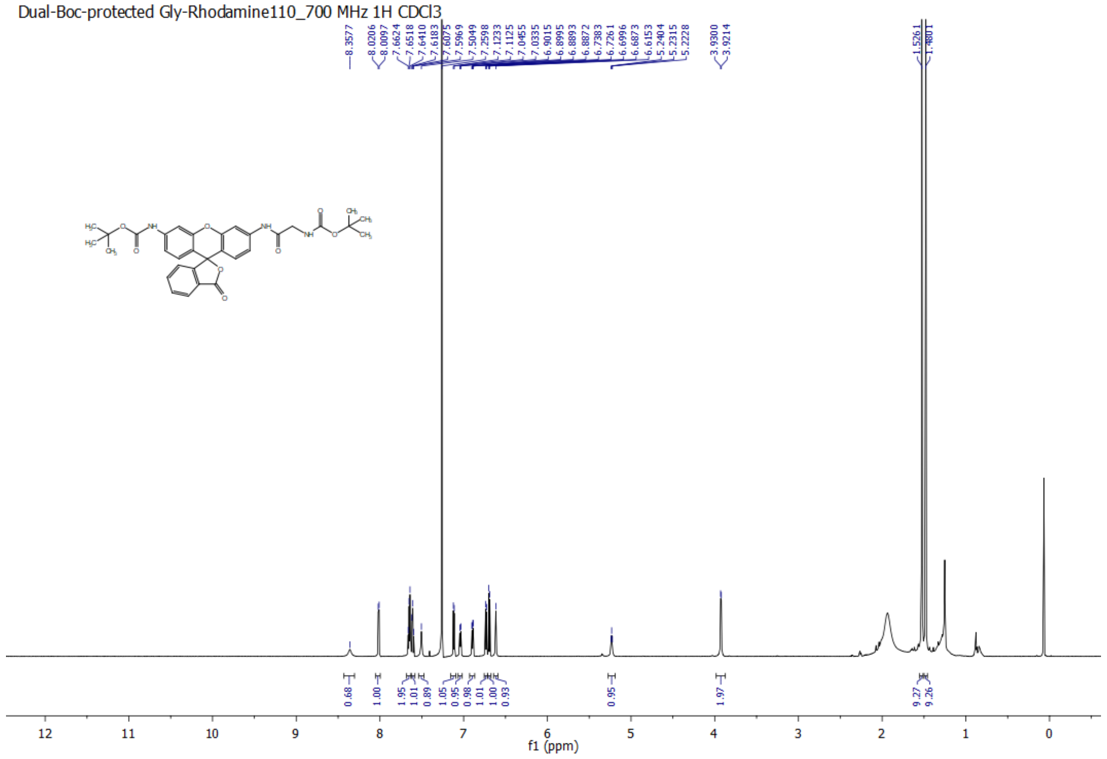

Dual-Boc-protected Gly-Rhodamine110_700 MHz 13C 176 MHz CDCl3

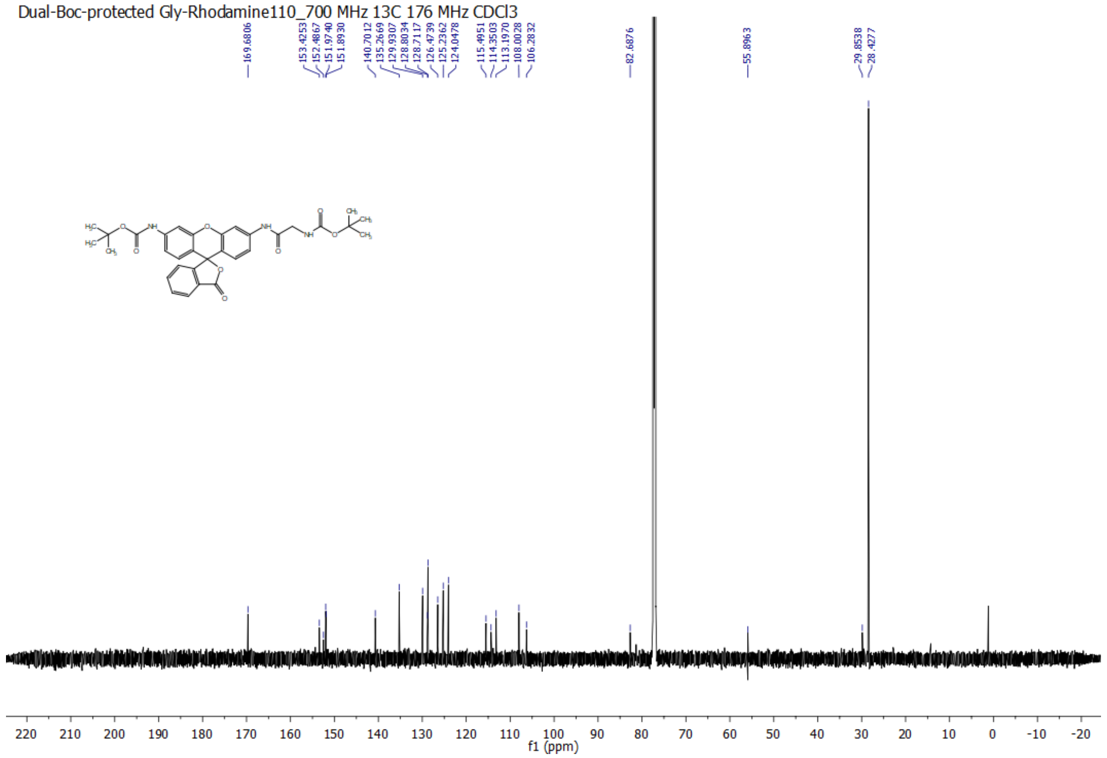

Gly-Rhodamine110 (RhoG) _600 MHz 1H D2O

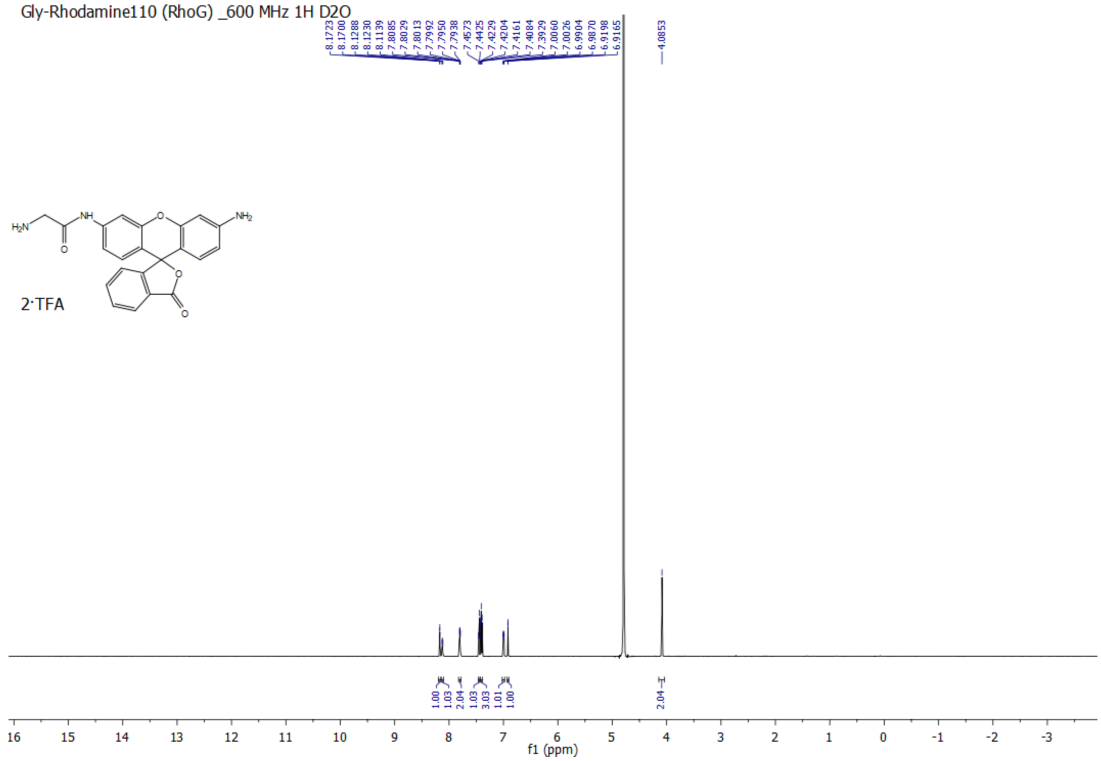

Gly-Rhodamine110 (RhoG) _151 MHz 13C D2O

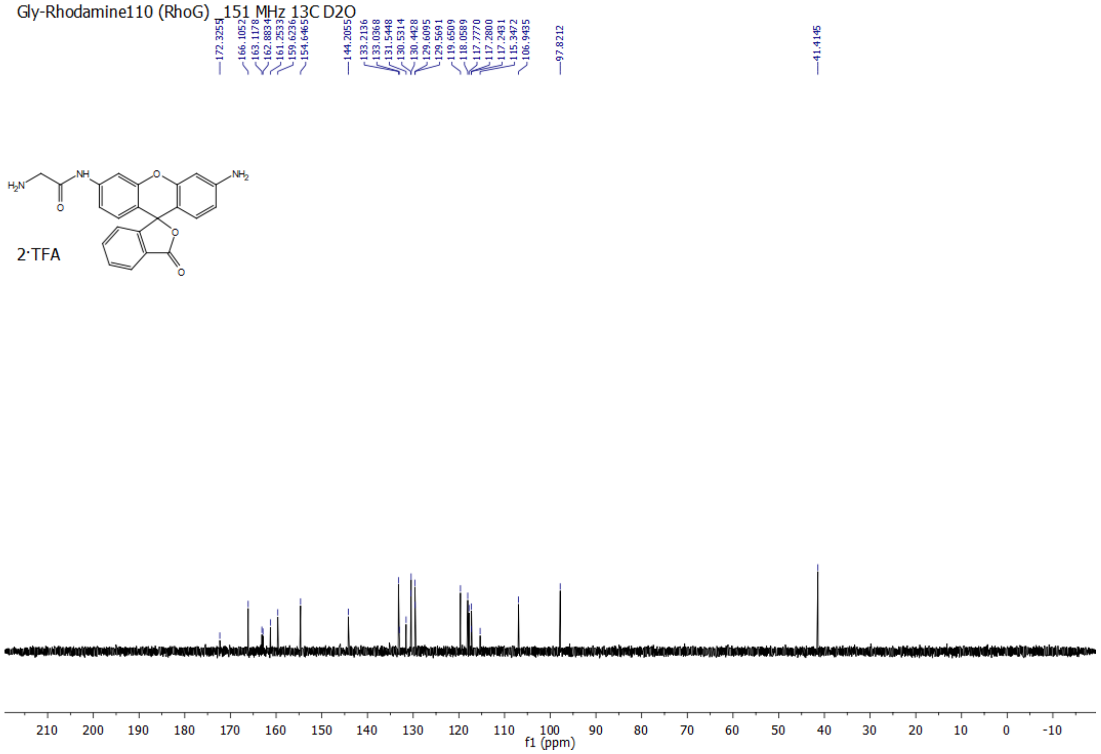

Tert-butyl N2-(((9H-fluoren-9-yl)methoxy)carbonyl)-N6-(tert-butoxycarbonyl)-L-lysylglycinate_600 MHz 1H CDCl3

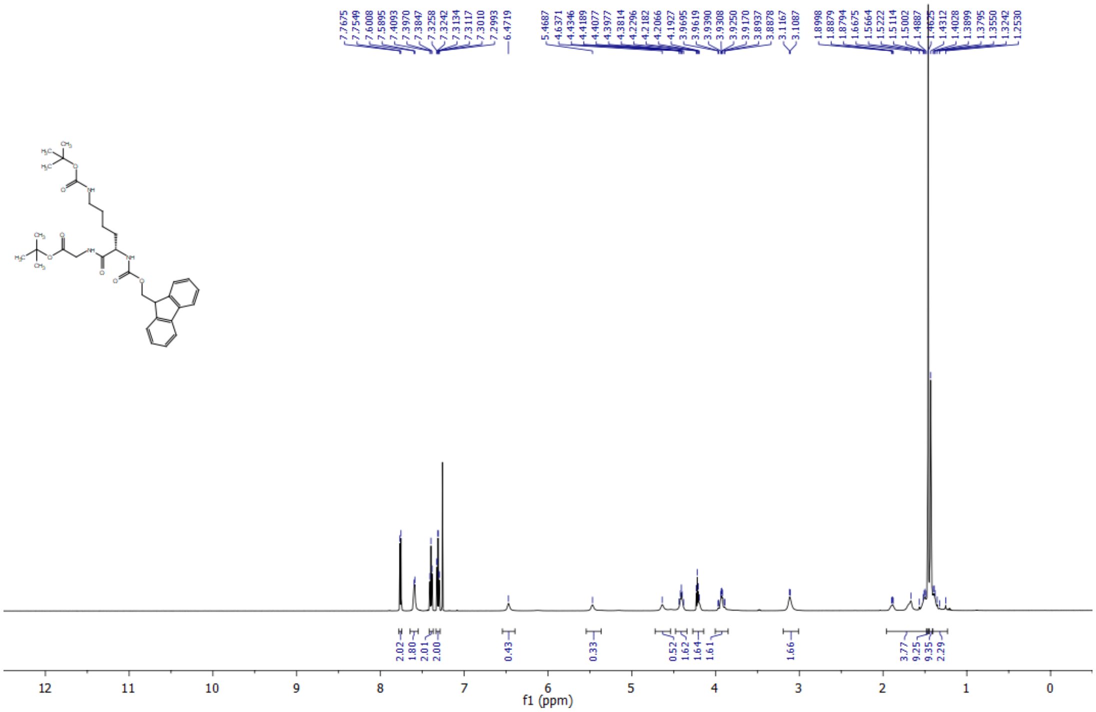

Tert-butyl N2-(((9H-fluoren-9-yl)methoxy)carbonyl)-N6-(tert-butoxycarbonyl)-L-lysylglycinate_151 MHz 13C CDCl3

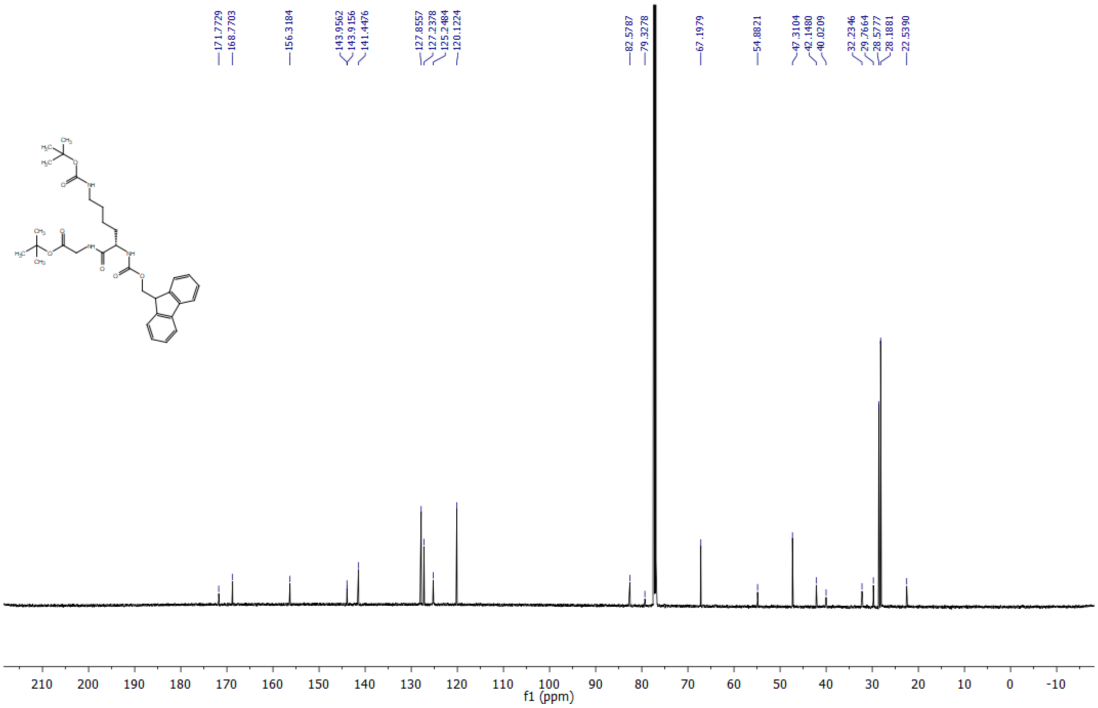

Tert-butyl N6-(tert-butoxycarbonyl)-L-lysylglycinate_600 MHz 1H CDCl3

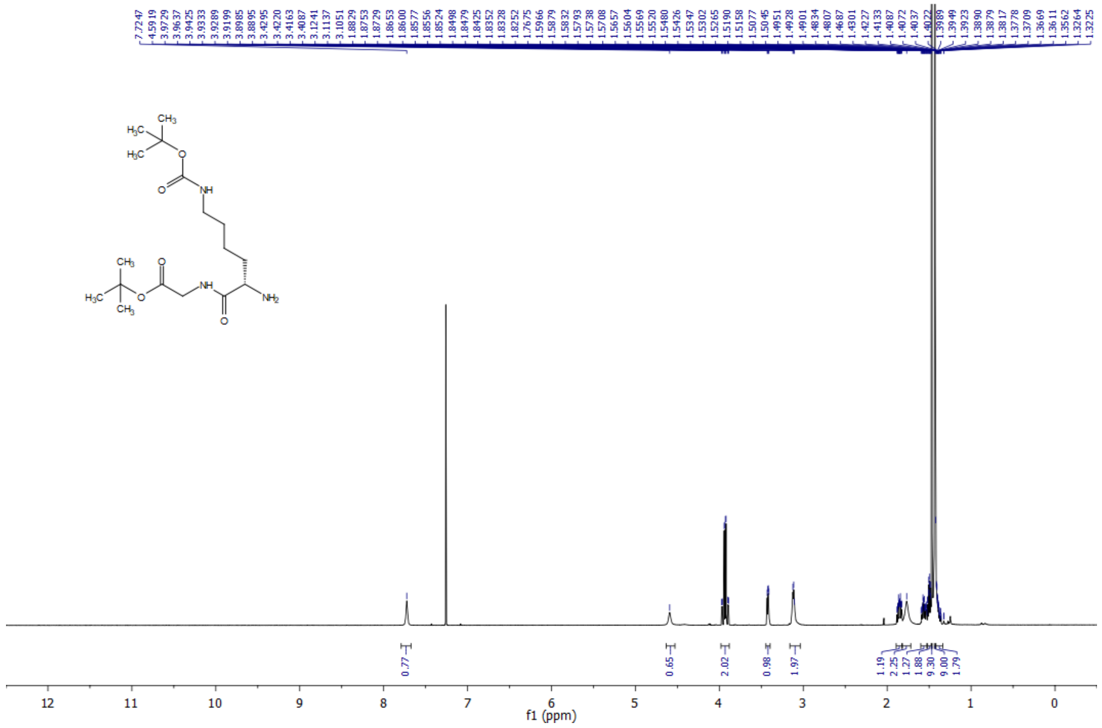

Tert-butyl N6-(tert-butoxycarbonyl)-L-lysylglycinate_151 MHz 13C CDCl3

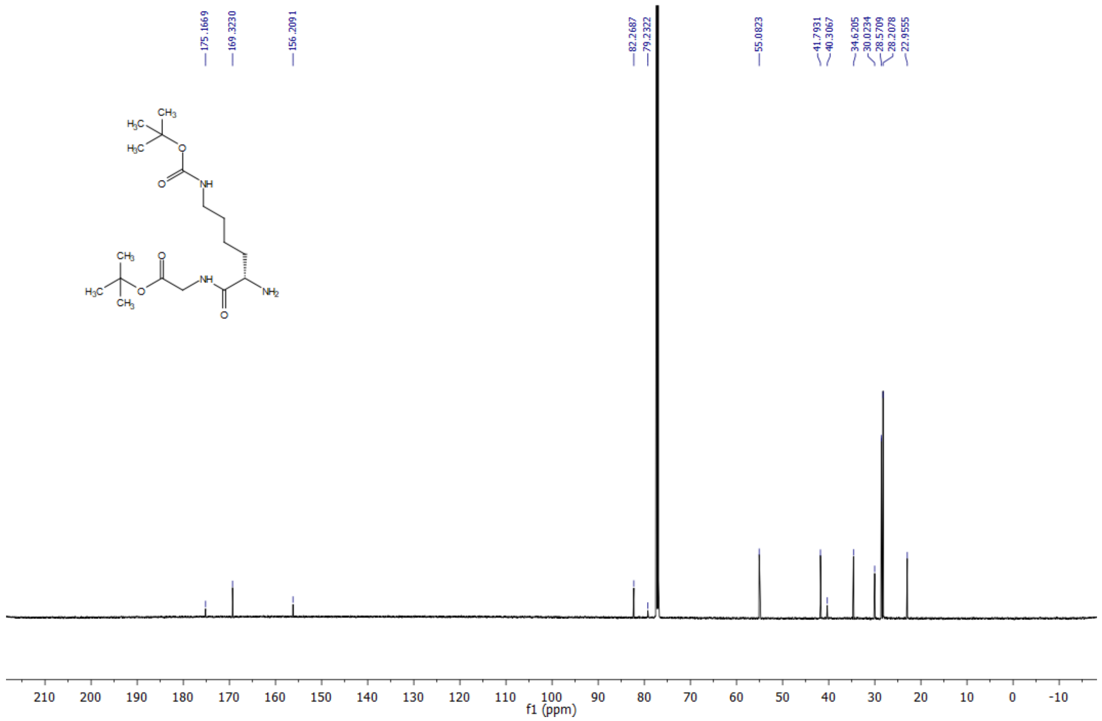

KG-TAMRA_700 MHz 1H D2O

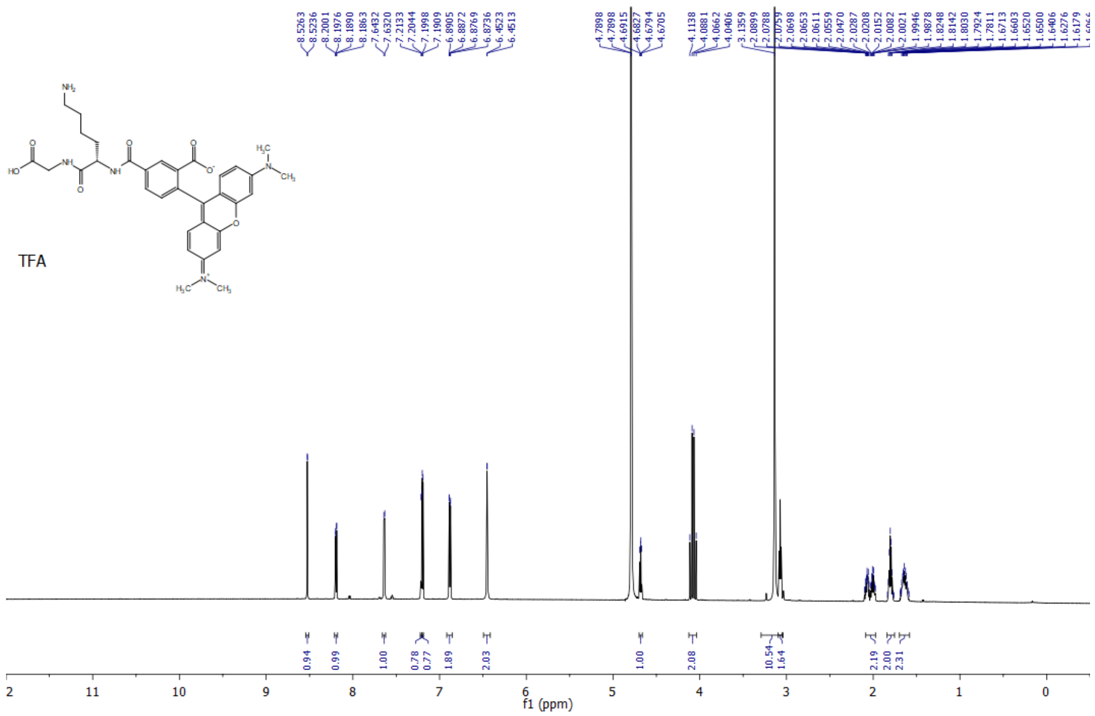

KG-TAMRA_176 MHz 13C D2O

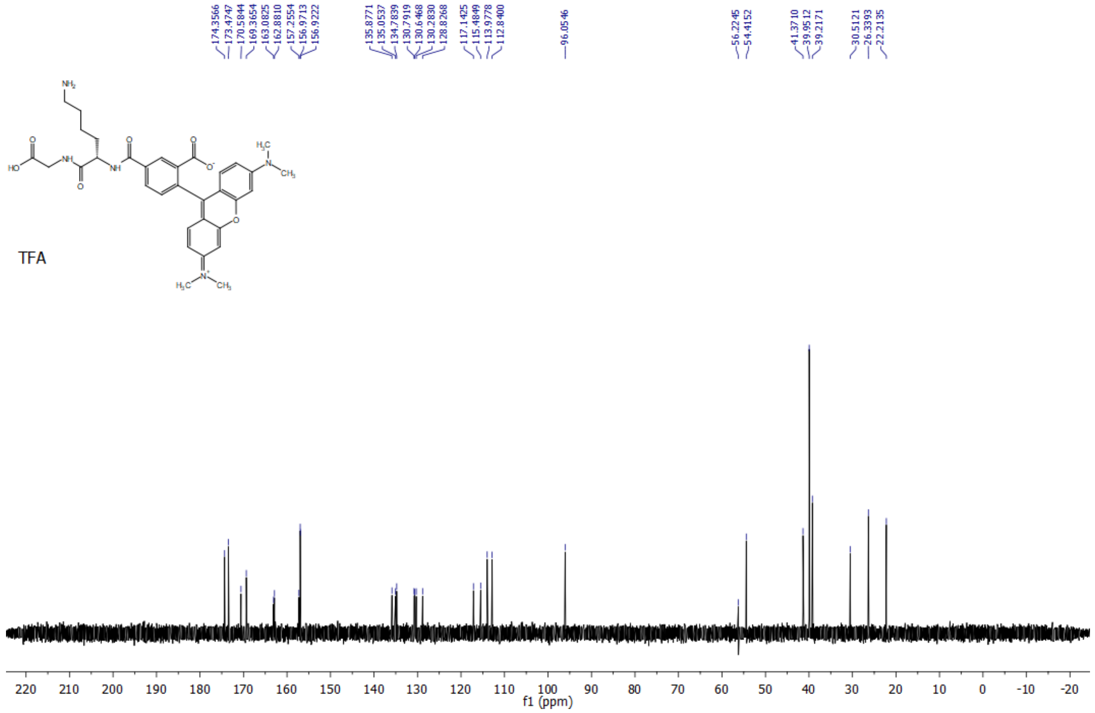

5-TAMRA_DMSO_700 MHz 1H

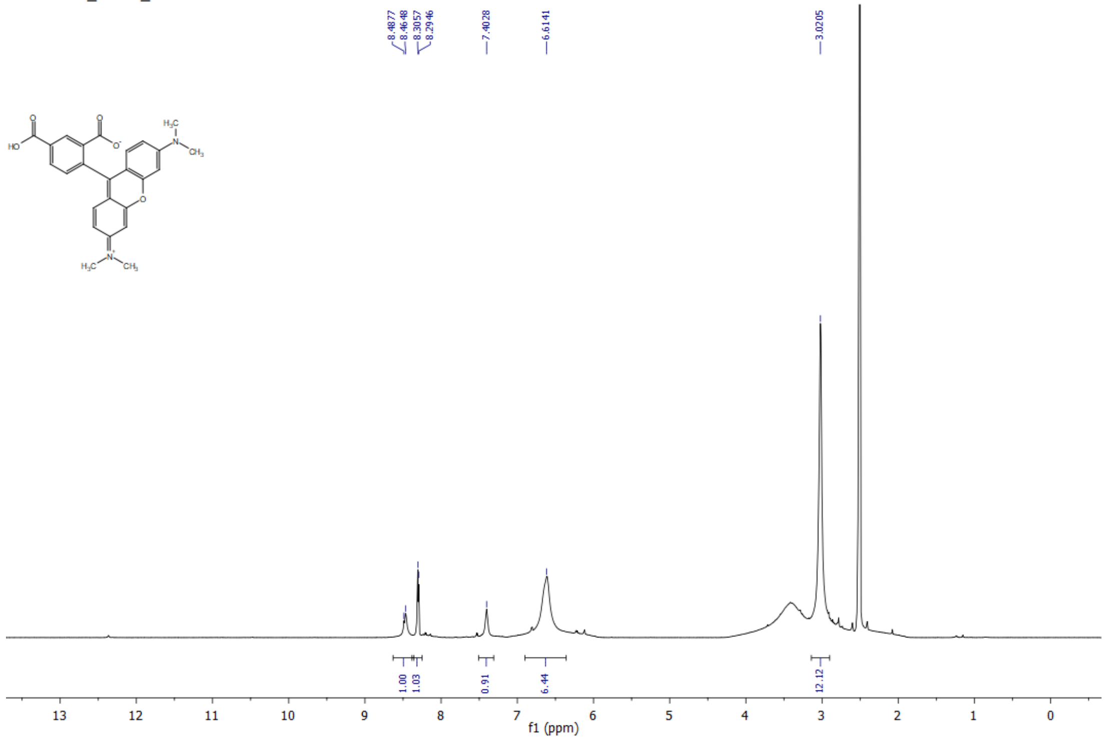

KG-TAMRA_700 MHz 1H D2O from solution synthesis

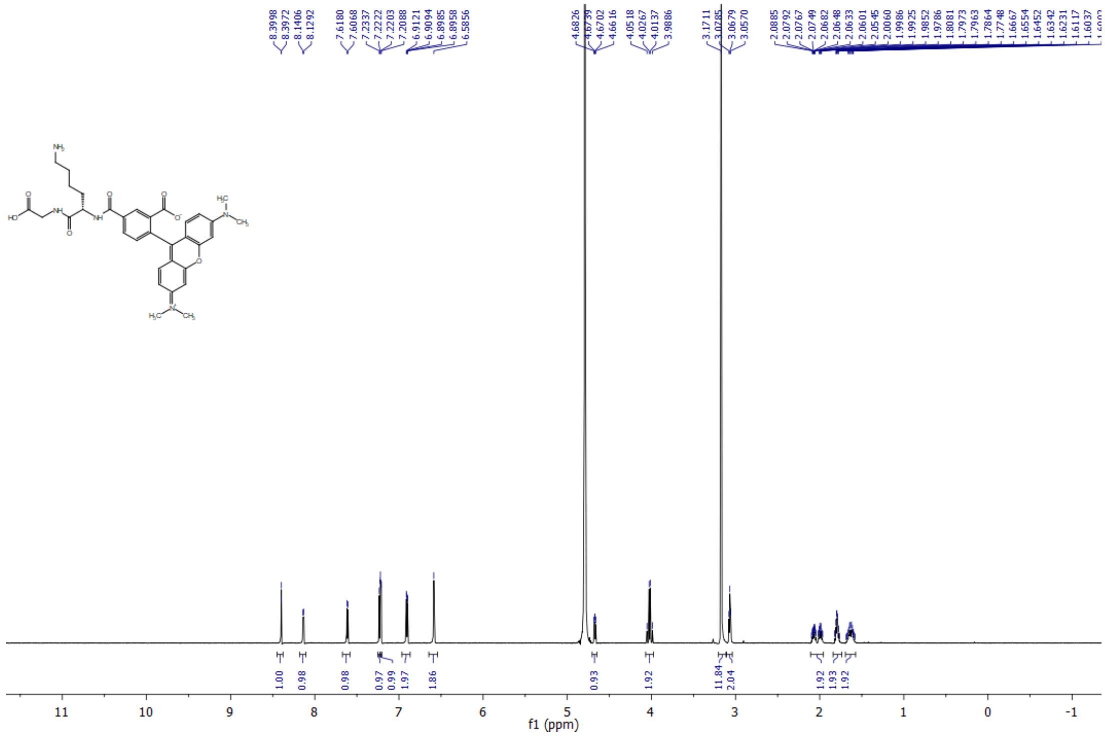

5. Uncropped gels

Figures 1I, 5B and 6A

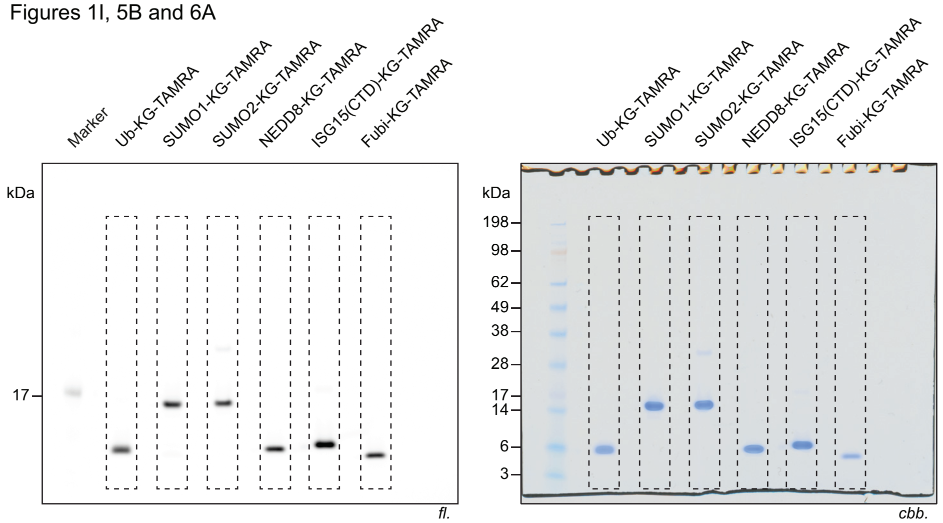

Figure S3B

Repurification of SUMO2-KG-TAMRA

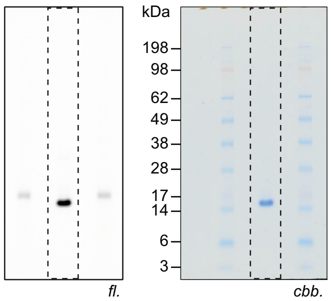

Figure 4C

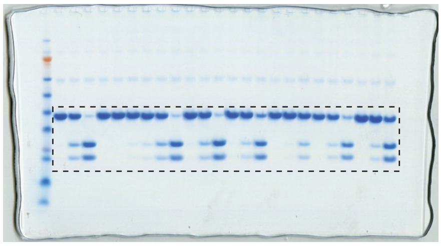

Figure 4D

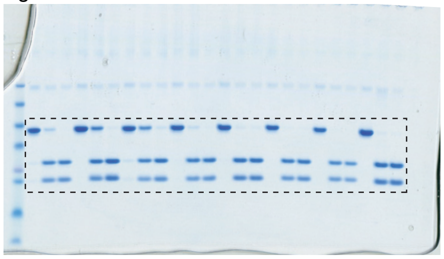

Figure 3B

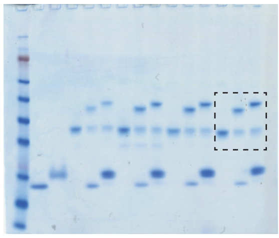

Figure S5J

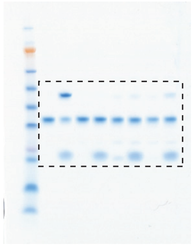

Figure S6C

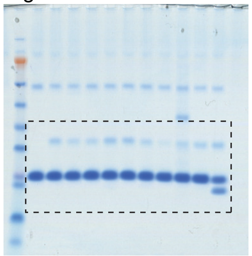

Figure S4D

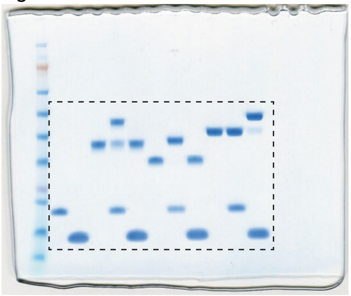

Figure S6G

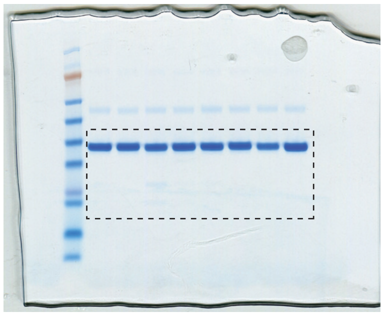

Figure S6H

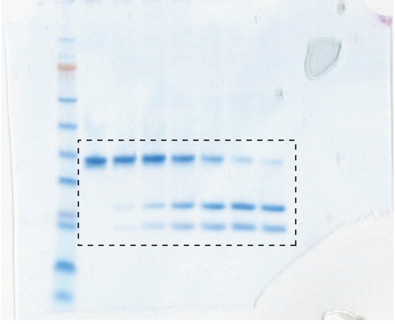

6. Intact protein mass spectra

m/z raw data for Figure S2B

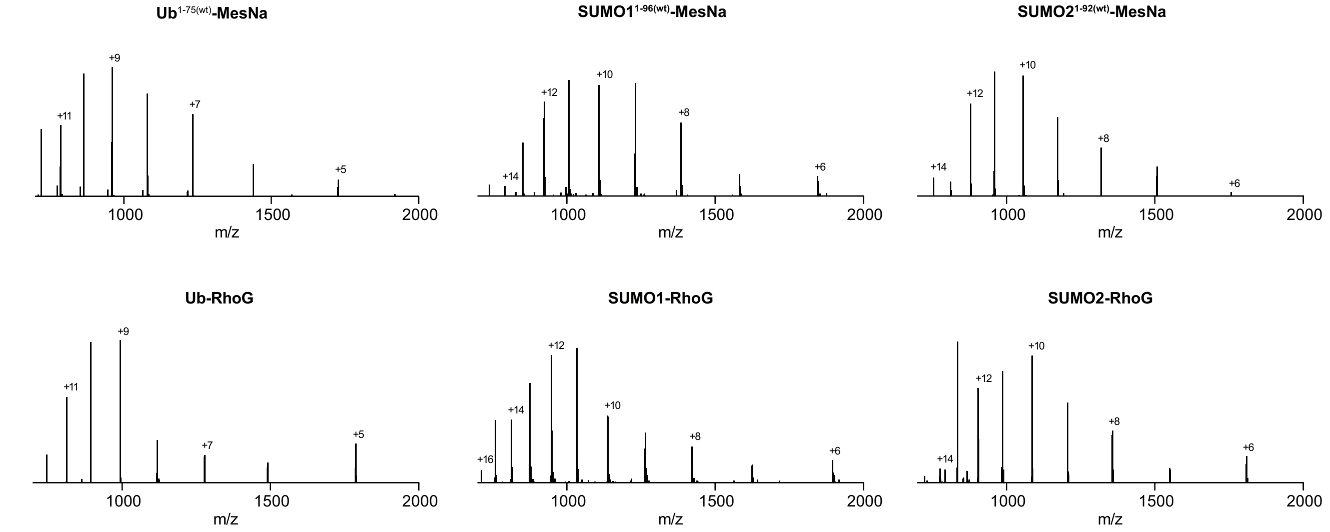

m/z raw data for Figure S3A

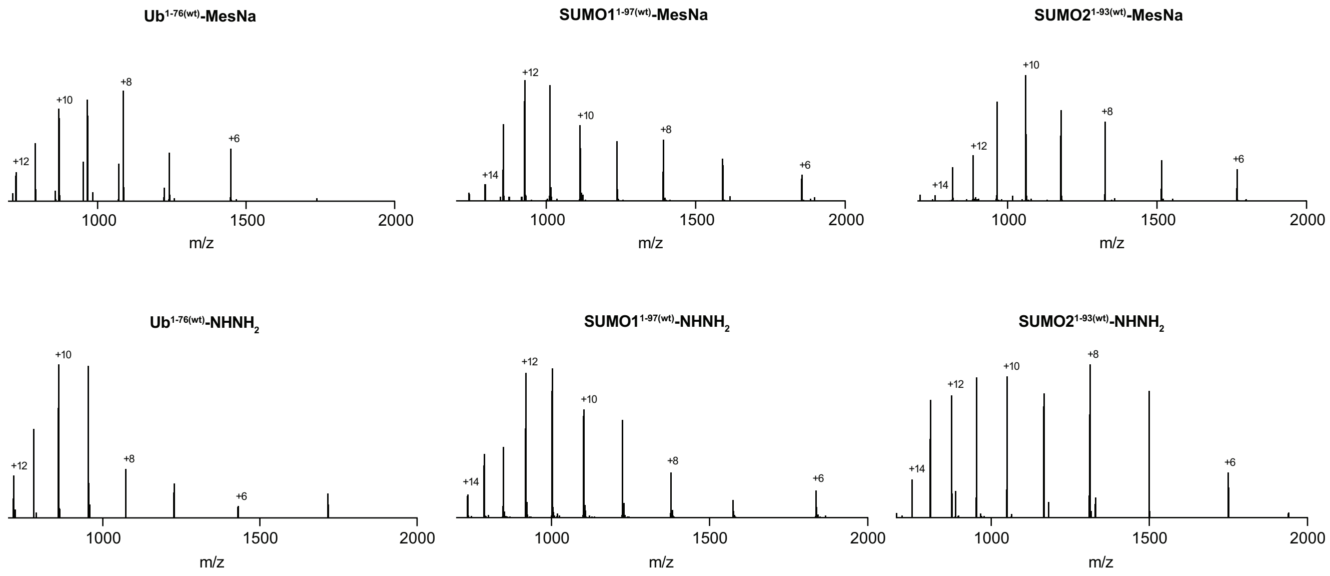

m/z raw data for Figures S7C and 7D

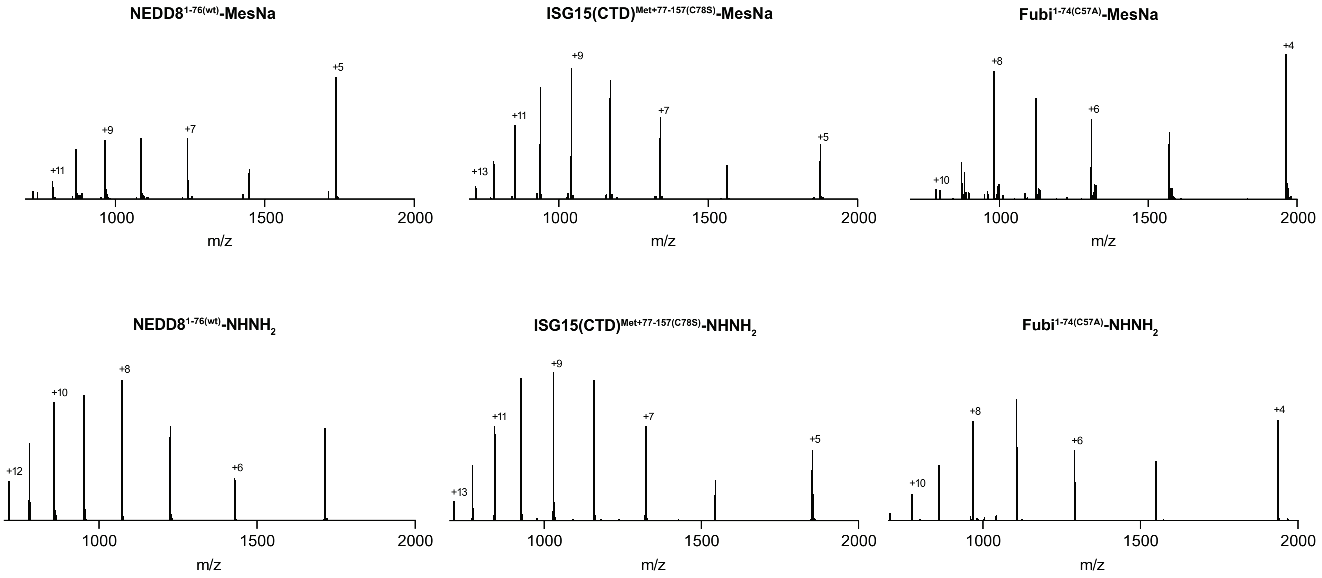

Protein mass data of samples in Figures 1J and 5A with higher injection volume

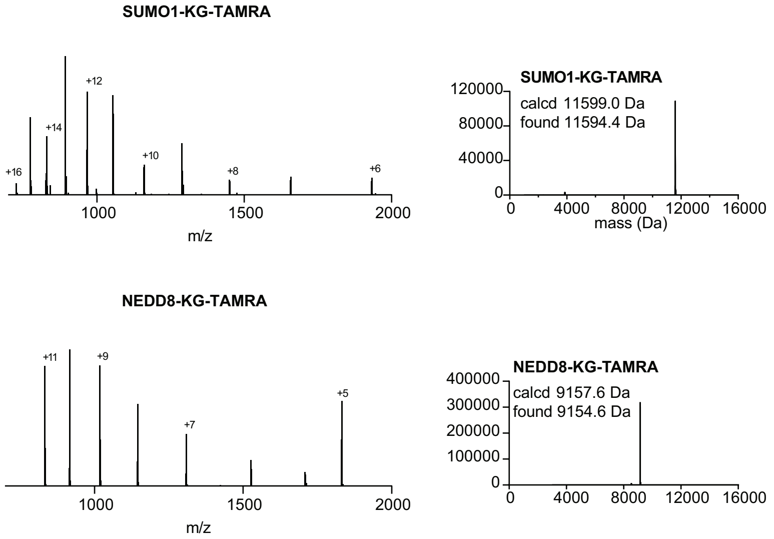

m/z raw data for Figures 1J, 5A and 6B

Ub-KG-TAMRA

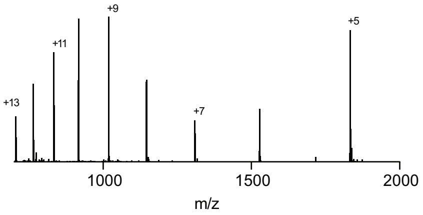

SUMO1-KG-TAMRA

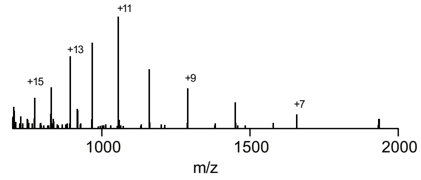

SUMO2-KG-TAMRA

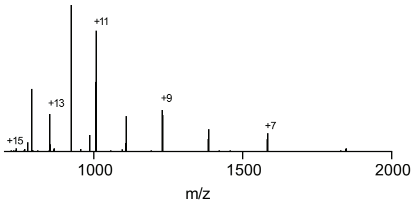

NEDD8-KG-TAMRA

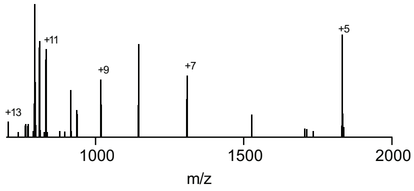

ISG15(CTD)-KG-TAMRA

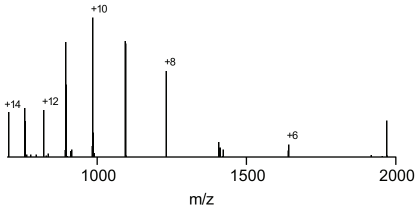

Fubi-KG-TAMRA

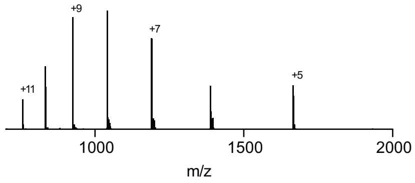

7. Supporting References

(1) Geurink, P. P.; El Oualid, F.; Jonker, A.; Hameed, D. S.; Ovaa, H. A General Chemical Ligation Approach Towards Isopeptide-Linked Ubiquitin and Ubiquitin-Like Assay Reagents. *Chembiochem* **2012**, *13*, 293.

(2) Hassiepen, U.; Eidhoff, U.; Meder, G.; Bulber, J. F.; Hein, A.; Bodendorf, U.; Lorthiois, E.; Martoglio, B. A sensitive fluorescence intensity assay for deubiquitinating proteases using ubiquitin-rhodamine110-glycine as substrate. *Anal Biochem* **2007**, *371*, 201.

(3) Ritorto, M. S.; Ewan, R.; Perez-Oliva, A. B.; Knebel, A.; Buhrlage, S. J.; Wightman, M.; Kelly, S. M.; Wood, N. T.; Virdee, S.; Gray, N. S.; Morrice, N. A.; Alessi, D. R.; Trost, M. Screening of DUB activity and specificity by MALDI-TOF mass spectrometry. *Nat Commun* **2014**, *5*, 4763.

(4) Fan, J.; Ye, Y.; Chu, G.; Zhang, Z.; Fu, Y.; Li, Y. M.; Shi, J. Semisynthesis of Ubiquitin and SUMO-Rhodamine 110-Glycine through Aminolysis of Boc-Protected Thioester Counterparts. *J Org Chem* **2019**, *84*, 14861.

(5) Klemm, T.; Ebert, G.; Calleja, D. J.; Allison, C. C.; Richardson, L. W.; Bernardini, J. P.; Lu, B. G.; Kuchel, N. W.; Grohmann, C.; Shibata, Y.; Gan, Z. Y.; Cooney, J. P.; Doerflinger, M.; Au, A. E.; Blackmore, T. R.; van der Heden van Noort, G. J.; Geurink, P. P.; Ovaa, H.; Newman, J.; Riboldi-Tunnicliffe, A.; Czabotar, P. E.; Mitchell, J. P.; Feltham, R.; Lechtenberg, B. C.; Lowes, K. N.; Dewson, G.; Pellegrini, M.; Lessene, G.; Komander, D. Mechanism and inhibition of the papain-like protease, PLpro, of SARS-CoV-2. *EMBO J* **2020**, *39*, e106275.

(6) Basters, A.; Geurink, P. P.; Rocker, A.; Witting, K. F.; Tadayon, R.; Hess, S.; Semrau, M. S.; Storici, P.; Ovaa, H.; Knobeloch, K. P.; Fritz, G. Structural basis of the specificity of USP18 toward ISG15. *Nat Struct Mol Biol* **2017**, *24*, 270.

(7) Pruneda, J. N.; Durkin, C. H.; Geurink, P. P.; Ovaa, H.; Santhanam, B.; Holden, D. W.; Komander, D. The Molecular Basis for Ubiquitin and Ubiquitin-like Specificities in Bacterial Effector Proteases. *Mol Cell* **2016**, *63*, 261.

(8) Hammler, D.; Marx, A.; Zumbusch, A. Fluorescence - Lifetime - Sensitive Probes for Monitoring ATP Cleavage. *Chem Eur J* **2018**, *24*, 15329.

(9) Kvach, M. V.; Stepanova, I. A.; Prokhorenko, I. A.; Stupak, A. P.; Bolibrukh, D. A.; Korshun, V. A.; Shmanai, V. V. Practical synthesis of isomerically pure 5-and 6-carboxytetramethylrhodamines, useful dyes for DNA probes. *Bioconjugate Chem* **2009**, *20*, 1673.

(10) Grethe, C.; Schmidt, M.; Kipka, G. M.; O'Dea, R.; Gallant, K.; Janning, P.; Gersch, M. Structural basis for specific inhibition of the deubiquitinase UCHL1. *Nat Commun* **2022**, *13*, 5950.

(11) O'Dea, R.; Kazi, N.; Hoffmann-Benito, A.; Zhao, Z.; Recknagel, S.; Wendrich, K.; Janning, P.; Gersch, M. Molecular basis for Ubiquitin/Fubi cross-reactivity in USP16 and USP36 underlying Fubi-S30 cleavage. *Nat Chem Biol* **2023**, https://doi.org/10.1038/s41589-023-01388-1.

(12) Friese, A.; Kapoor, S.; Schneidewind, T.; Vidadala, S. R.; Sardana, J.; Brause, A.; Forster, T.; Bischoff, M.; Wagner, J.; Janning, P.; Ziegler, S.; Waldmann, H. Chemical Genetics Reveals a Role of dCTP Pyrophosphatase 1 in Wnt Signaling. *Angew Chem Int Ed Engl* **2019**, *58*, 13009.

(13) Van Duyne, G. D.; Standaert, R. F.; Karplus, P. A.; Schreiber, S. L.; Clardy, J. Atomic structures of the human immunophilin FKBP-12 complexes with FK506 and rapamycin. *J Mol Biol* **1993**, *229*, 105.

(14) Bernier-Villamor, V.; Sampson, D. A.; Matunis, M. J.; Lima, C. D. Structural basis for E2-mediated SUMO conjugation revealed by a complex between ubiquitin-conjugating enzyme Ubc9 and RanGAP1. *Cell* **2002**, *108*, 345.

(15) Lois, L. M.; Lima, C. D. Structures of the SUMO E1 provide mechanistic insights into SUMO activation and E2 recruitment to E1. *EMBO J* **2005**, *24*, 439.

(16) Kabsch, W. Xds. *Acta Cryst* **2010**, *D66*, 125.
(17) Beilsten-Edmands, J.; Winter, G.; Gildea, R.; Parkhurst, J.; Waterman, D.; Evans, G. Scaling diffraction data in the DIALS software package: algorithms and new approaches for multi-crystal scaling. *Acta Cryst* **2020**, *D76*, 385.
(18) Evans, P. R.; Murshudov, G. N. How good are my data and what is the resolution? *Acta Cryst* **2013**, *D69*, 1204.
(19) Skubak, P.; Arac, D.; Bowler, M. W.; Correia, A. R.; Hoelz, A.; Larsen, S.; Leonard, G. A.; McCarthy, A. A.; McSweeney, S.; Mueller-Dieckmann, C.; Otten, H.; Salzman, G.; Pannu, N. S. A new MR-SAD algorithm for the automatic building of protein models from low-resolution X-ray data and a poor starting model. *IUCrJ* **2018**, *5*, 166.
(20) Sheldrick, G. M. Experimental phasing with SHELXC/D/E: combining chain tracing with density modification. *Acta Cryst* **2010**, *D66*, 479.
(21) Cowtan, K. Recent developments in classical density modification. *Acta Cryst* **2010**, *D66*, 470.
(22) Murshudov, G. N.; Skubak, P.; Lebedev, A. A.; Pannu, N. S.; Steiner, R. A.; Nicholls, R. A.; Winn, M. D.; Long, F.; Vagin, A. A. REFMAC5 for the refinement of macromolecular crystal structures. *Acta Cryst* **2011**, *D67*, 355.
(23) Emsley, P.; Lohkamp, B.; Scott, W. G.; Cowtan, K. Features and development of Coot. *Acta Cryst* **2010**, *D66*, 486.
(24) Afonine, P. V.; Grosse-Kunstleve, R. W.; Echols, N.; Headd, J. J.; Moriarty, N. W.; Mustyakimov, M.; Terwilliger, T. C.; Urzhumtsev, A.; Zwart, P. H.; Adams, P. D. Towards automated crystallographic structure refinement with phenix.refine. *Acta Cryst* **2012**, *D68*, 352.
(25) McCoy, A. J. Solving structures of protein complexes by molecular replacement with Phaser. *Acta Cryst* **2007**, *D63*, 32.
(26) Werner, A.; Flotho, A.; Melchior, F. The RanBP2/RanGAP1(star)SUMO1/Ubc9 Complex Is a Multisubunit SUMO E3 Ligase. *Mol Cell* **2012**, *46*, 287.
